# *In silico* evidence for functional specialization after genome duplication in yeast

**DOI:** 10.1111/j.1567-1364.2008.00451.x

**Published:** 2008-11-03

**Authors:** Ossi Turunen, Ralph Seelke, Jed Macosko

**Affiliations:** 1 Department of Biotechnology and Chemical Technology, Helsinki University of TechnologyEspoo, Finland; 2 Department of Biology and Earth Sciences, University of Wisconsin-SuperiorSuperior, WI, USA; 3 Department of Physics, Wake Forest UniversityWinston-Salem, NC, USA

**Keywords:** gene duplication, yeast genome, protein evolution, sequence analysis, structural analysis

## Abstract

A fairly recent whole-genome duplication (WGD) event in yeast enables the effects of gene duplication and subsequent functional divergence to be characterized. We examined 15 ohnolog pairs (i.e. paralogs from a WGD) out of *c*. 500 *Saccharomyces cerevisiae* ohnolog pairs that have persisted over an estimated 100 million years of evolution. These 15 pairs were chosen for their high levels of asymmetry, i.e. within the pair, one ohnolog had evolved much faster than the other. Sequence comparisons of the 15 pairs revealed that the faster evolving duplicated genes typically appear to have experienced partially – but not fully – relaxed negative selection as evidenced by an average nonsynonymous/synonymous substitution ratio (d*N*/d*S*_avg_=0.44) that is higher than the slow-evolving genes' ratio (d*N*/d*S*_avg_=0.14) but still <1. Increased number of insertions and deletions in the fast-evolving genes also indicated loosened structural constraints. Sequence and structural comparisons indicated that a subset of these pairs had significant differences in their catalytically important residues and active or cofactor-binding sites. A literature survey revealed that several of the fast-evolving genes have gained a specialized function. Our results indicate that subfunctionalization and even neofunctionalization has occurred along with degenerative evolution, in which unneeded functions were destroyed by mutations.

## Introduction

The duplication of genetic elements is nearly a century-old concept. Its mechanism and role in evolution were already widely discussed 50–70 years ago (for reviews, see Stephens, 1951; Taylor & Raes, 2004). Susumu Ohno's classic study in 1970 has inspired more recent interest in gene duplication (Ohno, 1970). Currently, gene duplication is understood as a major force supplying evolution with raw genetic material and driving the molecular innovations necessary for increasing cellular and intercellular complexity. The recent availability of a large number of genome sequences now offers a possibility to look more closely at the nature and fate of duplicated genes.

Recently, a proposed whole-genome duplication (WGD) has been confirmed in yeast (Kellis *et al.*, 2004), which is estimated to have occurred 100 million years ago (MYA) – after the ancestors of *Candida glabrata, Saccharomyces cerevisiae*, and other *Saccharomyces* species branched off from the lines that led to *Saccharomyces kluyveri, Kluyveromyces waltii* (also called *Lachancea waltii*; Kurtzman, 2003), and other yeast variants. In this scheme, an ancestor of the WGD lineages duplicated all of its original genes, and then subsequent generations lost most of the added genetic material. The result in *S. cerevisiae* is a genome with *c*. 5500 genes, in which about 500 duplicated gene pairs originated from the WGD (Dietrich *et al.*, 2004; Kellis *et al.*, 2004). Because these paralogs are all the same age, Ken Wolfe has suggested the term ‘ohnologs’, in honor of Susumu Ohno, to distinguish them from other paralogs that result from small-scale gene duplication events, and in this study we will use this terminology (Wolfe, 2004).

Interestingly, although the ohnologs in *S. cerevisiae* share a common history, they are comprised of two populations, which differ dramatically in the amount of sequence similarity between the paired genes. On the one hand, there is a population of ohnologs that have very similar sequences. On the other, there are many ohnologs that share very little sequence identity (sometimes even to the point where a blastp search would fail to link the two genes) and, most often, this vast difference in sequence is due to only one of the two genes diverging rapidly, as determined by comparison with an outgroup (Conant & Wagner, 2003; Kellis *et al.*, 2004). By studying the duplicated yeast genes, it has been proposed that the asymmetric sequence divergence between duplicates is correlated with asymmetric functional divergence (Langkjaer *et al.*, 2003; Kim & Yi, 2006). An important endeavor, then, is to understand the nature of the differences in this second, maximally asymmetric, population of ohnologs – differences that have occurred under conditions favorable for the evolution of new functions (neofunctionalization) or for the partitioning of old functions (subfunctionalization).

Large-scale molecular evolution trends among duplicated yeast genes have been examined in numerous studies (Lynch & Conery, 2000; Wagner, 2002; Langkjaer *et al.*, 2003; Drummond *et al.*, 2005; He & Zhang, 2005; Hughes & Friedman, 2005; Conant & Wolfe, 2006; Byrne & Wolfe, 2007; Tirosh & Barkai, 2007). Large-scale structural prediction has also been reported for the yeast proteome (Malmstrom *et al.*, 2007). Very recently, Wapinski *et al.* (2007) analyzed how the duplicated genes are distributed between functional gene ontology categories in yeasts and concluded that the duplicated genes rarely diverge with respect to biochemical function, but typically diverge with respect to regulatory control. Adopting a different approach, we have used structural modeling in combination with sequence analysis and information on reported biochemical and cellular functions in order to investigate the evolutionary fate of 15 maximally asymmetric ohnologs. We analyzed possible active site and cofactor-binding residues and found that these residues in the fast diverger have substantially changed in about half of the cases. Drawing from previously published studies of the function and expression of these ohnologs, it is clear that both neofunctionalization and subfunctionalization have occurred between these paired genes. We could detect how the divergence between the duplicates has changed the pattern of protein's subfunctions. As far as we know, this kind of analysis has not been applied in any larger scale study of the evolutionary fate of duplicated genes.

## Materials and methods

*Saccharomyces cerevisiae* gene sequences and general information about the genes were obtained from Saccharomyces Genome Database (http://db.yeastgenome.org/cgi-bin/seqTools). Protein divergence absolute and relative rates for all pairs and their *K. waltii* ortholog and *K. waltii* gene sequences were kindly provided by Dr Kellis (Duplicated Pairs and predicted ORFs documents, respectively). Fifteen gene pairs from the 23 ohnolog pairs with the highest protein divergence rates between ohnologs – as determined by Kellis *et al.* (2004) (divergence rates are shown in Supporting Information, S9, and in the Duplicated Pairs file in Kellis *et al.*: http://www.nature.com/nature/journal/v428/n6983/extref/S9_Trees/Duplicated_Pairs.xls) – were chosen for analysis. The selected duplicated gene pairs are all from the group of 76 gene pairs out of 457 gene pairs in the study of Kellis *et al.* (2004) that showed accelerated protein evolution relative to *K. waltii*.

Sequence alignments were performed using clustal w (default parameters: Blosum scoring matrix, opening gap penalty 10, end gap penalty 10, extending gap penalty 0.05 and separation gap penalty 0.05) coupled to the blast Network Service of Swiss Institute of Bioinformatics [SIB BLAST Network Service (http://tw.expasy.org/tools/blast/)]. The blast searches were carried out primarily with the *K. waltii* protein sequences. The insertions and deletions (indels) were determined relative to the corresponding *K. waltii* gene, and the number of indels and their length distribution is shown in Table 3. Whenever possible, the structural positions of indels were deduced (Table S1A). Prediction of cellular location signal was carried out primarily at the Yeast Protein Localization Server (http://bioinfo.mbb.yale.edu/genome/localize/).

**Table 3:** Size distribution of insertions and deletions (indels) among the 15 duplicated gene pairs

Indel size	1	2	3	4	5	6	7	8	>10	Totals	Amino acids*
S INS	3	–	1	–	–	–	–	–	–	4	6
S DEL	1	1	1	1	–	–	–	–	2	6	40
F INS	19	5	2	–	1	2	1	–	–	30	59
F DEL	6	4	3	1	1	3	1	4	6	29	221
Totals	29	10	7	2	2	5	2	4	8	69	326

The indels were determined in comparison to the corresponding *Kluyveromyces waltii* gene. The N- and C-terminal length variation was excluded.

* Number of amino acids changed by the indels.

S, slow-evolving gene; F, fast-evolving gene; INS, insertion; DEL, deletion.

Nonsynonymous (d*N*) and synonymous (d*S*) substitution rates were estimated for the divergence of the two yeast genes in the duplicated pair from the corresponding *K. waltii* gene (Table S1B). We used the overall d*N*/d*S* ratio of each gene in order to determine whether the fast-evolving genes are, on the whole, protected by selection. In other words, our goal was not to find the specific sites or regions that are under selection – an interesting question in its own right that would require a further study. mega 3.1 (2003) and the modified Nei–Gojobori method with a Jukes–Kantor correction and a transition/transversion ratio of 3 were used for estimating amino acid and nucleotide substitution parameters d*N* and d*S* (Kumar *et al.*, 2004), and SEs were calculated from 500 bootstrap replicates.

The SWISS-MODEL modeling server was used to generate structural models for 15 out of the 30 yeast proteins that were studied (Schwede *et al.*, 2003). In addition to these 15 models, published structures were available for eight of the proteins (see Table S1A). Models were evaluated at a level that did not require the highest possible structural accuracy to tease out subtle effects. Rather, we only examined the effects of more radical amino acid changes.

## Results

The evolutionary patterns of 15 pairs of duplicated *S. cerevisiae* genes (Table 1; see Table 2 for systematic names) were inferred from three lines of evidence: (1) sequence comparisons, with an emphasis on the accumulation of insertions and deletions (indels), (2) estimates of the ratio of nonsynonymous to synonymous substitutions (d*N*/d*S*), and (3) analyses of the amino acid changes in key sites. All three of these approaches utilized the sequence of the outgroup *K. waltii*, which diverged from the line leading to *S. cerevisiae* before the WGD. This outgroup sequence was used to estimate the extent of evolutionary change in either gene of the *S. cerevisiae* ohnolog pairs (Kellis *et al.*, 2004). The protein divergence among the 15 gene pairs is on average 385% between the two yeast genes, 399% between the fast-evolving genes and *K. waltii* genes, and 101% between the slow-evolving genes and the *K. waltii* genes (calculated from the supplemental information of Kellis *et al.*, 2004). Notably, the fast-evolving gene YHL012W shows a remarkably higher degree of divergence from the slow-evolving gene UGP1 (891%) and from the *K. waltii* gene (1206%) relative to any of the other gene pairs (Kellis *et al.*, 2004). Otherwise, all gene pairs follow the same trend wherein the divergence is much higher between the fast-evolving gene and the *K. waltii* gene than between the slow-evolving gene and the *K. waltii* gene. Thus, the ohnolog divergence between the slow- and fast-evolving yeast genes is approximately as high as the ortholog divergence between fast-evolving yeast genes and *K. waltii* genes.

**Table 2:** Predicted and Experimental Locations

Gene 1 (slow evolving)				Gene 2 (fast evolving)			
Name: common/ systematic	Predicted location*	Huh *et al.* location(s)†	Other experimental location(s)‡	Name: common/ systematic	Predicted location*	Huh *et al.* location(s)†	Other experimental location(s)‡
UGP1/YKL035W	Weak nucleus	Cytoplasm	–	YHL012W/YHL012W	Nucleus	–	–
PST2/YDR032C	Endoplasmic reticulum (ER)	Cytoplasm (punctuate composite)	Cytoplasm (punctuate), chromatin	RFS1/YBR052C	No clear prediction	Cytoplasm (punctuate) composite)	Cytoplasm (punctuate), chromatin
MCK1/YNL307C	Mitochondrial	Cytoplasm, nucleus;	Centromere	YGK3/YOL128C	Nucleus	–	–
ACC1/YNR016C	Cytoplasm	Cytoplasm (punctuate composite)	Cytoplasm	HFA1/YMR207C	Mitochondria	Mitochondria	Mitochondria
RNR2/YJL026W	Cytoplasm, nucleus	Cytoplasm, nucleus	Cytoplasm, nucleus	RNR4/YGR180C	Cytoplasm, nucleus	Cytoplasm, nucleus	Cytoplasm, nucleus
CET1/YPL228W	Nucleus	Nucleus	Nucleus	CTL1/YMR180C	Mitochondria, nucleus	–	Cytoplasm, nucleus
VPS21/YOR089C	ER	Cytoplasm, nucleus	Transport vesicles	YPT53/YNL093W	ER	–	Transport vesicles
SEC14/YMR079W	Cytoplasm	Cytoplasm, nucleus	Cytoplasm	SFH1/YKL091C	Nucleus	Cytoplasm, nucleus	Nucleus
SLT2/YHR030C	Nucleus	Cytoplasm, nucleus	Nucleus, bud tip	YKL161C/YKL161C	Nucleus	–	–
GCS1/YDL226C	Cytoplasm	Cytoplasm	–	SPS18/YNL204C	Nucleus	–	–
CDC19/YAL038W	Cytoplasm	Cytoplasm	Cytoplasm	PYK2/YOR347C	Nucleus	Cytoplasm	Cytoplasm, mitochondria
ADH1/YOL086C	Cytoplasm	–	Cytoplasm	ADH5/YBR145W	Nucleus	Cytoplasm, nucleus	cytoplasm, nucleus
GRS1/YBR121C	Cytoplasm	Cytoplasm	Cytoplasm, mitochondria	GRS2/YPR081C	Nucleus	Cytoplasm	Cytoplasm
ERV14/YGL054C	Integral membrane	ER, vacuole	ER–golgi	ERV15/YBR210W	Integral membrane	–	–
FEN1/YCR034W	Integral membrane	ER	ER	ELO1/YJL196C	Integral membrane	–	ER

* Prediction of cellular location signal was done primarily at the Yeast Protein Localization Server (http://bioinfo.mbb.yale.edu/genome/localize/).

† Location of GFP-tagged proteins from Huh *et al.* (2003).

‡ References providing the location are found in the Supporting Information.

**Table 1:** Divergence characteristics of duplicated yeast genes

Gene pair	*K. waltii* gene	Amino acids	pI*	Identity of y2–y1† (%)	Identity with *K. waltii*‡ (%)	Indels§	d*N*/d*S* [SE]¶
*UGP1*	8105	499	7.46		89	1	0.065 [0.010]
*YHL012W*		493	5.08	41	41	8	0.524 [0.066]
*PST2*	23 042	198	5.61		77	0	0.183 [0.040]
*RFS1*		210	5.03	47	54	5	0.206 [0.088]
*MCK1*	22 001	375	8.92		78	0	0.115 [0.021]
*YGK3*		375	7.55	44	42	4	0.461 [0.076]
*ACC1*	6157	2233	6.22		81	3	0.156 [0.011]∥
*HFA1*		2273	8.05	55	55	11	0.685 [0.041]∥
*RNR2*	15 007	399	5.01		83	0	0.119 [0.022]
*RNR4*		345	4.96	55	56	3	0.497 [0.065]
*CET1*	24 238	549	5.36		57	0#	0.163 [0.030]
*CTL1*		320	10.48	21	21#	9#	1.299 [0.222]
*VPS21*	2978	210	5.12		78	2	0.112 [0.039]
*YPT53*		220	4.99	64	57	4	0.291 [0.065]
*SEC14*	7837	304	5.26		84	0	0.082 [0.022]**
*SFH1*		310	7.95	64	64	1	0.254 [0.048]**
*SLT2*	5576	484	5.07		76	*c*. 2††	0.092 [0.021]
*YKL161C*		433	6.27	53	57	*c*. 2††	0.348 [0.048]
*GCS1*	4569	352	5.78		62	2	0.238 [0.041]
*SPS18*		300	8.15	32	27	*c*. 6	0.830 [0.124]
*CDC19*	6945	500	7.66		86	0	0.258 [0.039]
*PYK2*		506	6.90	71	70	0	0.168 [0.023]
*ADH1*	23 198	346	6.66		86	1	0.217 [0.038]
*ADH5*		351	6.34	76	74	0	0.185 [0.029]
*GRS1*	3922	667	5.88		82	0	0.127 [0.016]
*GRS2*		618	7.50	59	56	4	0.329 [0.037]
*ERV14*	1862	138	6.93		84	0	0.097 [0.030]
*ERV15*		142	8.04	62	63	0	0.224 [0.067]
*FEN1*	13 644	347	10.35		78	0	0.132 [0.025]
*ELO1*		310	10.2	59	58	2	0.345 [0.049]

Numerical parameters are shown to measure the divergence of slow (upper gene) and fast (lower gene) evolving genes from the corresponding *Kluyveromyces waltii* gene used as an outgroup. Sequence identity, number of indels and d*N*/d*S* ratio are calculated from the comparison with the singleton *K. waltii* gene. Lower in the gene pair is the fast-evolving gene.

* Theoretical pI.

† y1, slow-evolving yeast gene; y2, fast-evolving yeast gene.

‡ Identity with the corresponding single *K. waltii* gene.

§ Number of intragenic insertions and deletions when compared to *K. waltii* gene.

¶ Modified Nei–Gojobori method with Jukes–Kantor correction (transition/transversion ratio was 3) was used to estimate the pairwise distances to the gene (analysis using mega 3.1). SE: standard error.

∥ The differing d*N*/d*S* ratios are mainly due to differences outside the BC and CT domains (see Table S1B).

# Concern the overlapping regions between *CET1, CTL1* and *K. waltii* gene (whereas the pI value concerns the full protein of *CET1*). In calculating the d*N*/d*S* ratio, the regions corresponding to the 54 N-terminal amino acids in *CTL1* were excluded.

** The corresponding *K. waltii* gene (no: 7837) is apparently missing 25% of its sequence from the N-terminus; the reported sequence analysis concerns only the region present in *K. waltii* gene.

†† The alignments are unclear in the C-terminal region in which the indels occur.

### Partially relaxed selection

When a gene is not under selective pressure, it is free to undergo mutations in a random manner (Kimura, 1983). Under these circumstances, sequence changes that result in nonsynonymous amino acid substitutions (d*N*) would be expected to occur approximately as frequently as those that produce synonymous amino acid substitutions (d*S*) (i.e. the d*N*/d*S* ratio should be *c*. 1). If a gene provides a fitness advantage, then some of the nonsynonymous substitutions would result in a reduction in function, and would thus be selected against. Thus, a d*N*/d*S* ratio <1 is an indication that the gene is undergoing purifying selection. A d*N*/d*S* ratio that is greater than unity has been traditionally seen as an indication that the gene may have evolved a new function that has a selective advantage, although more developed statistical methods are now used to detect positive selection (Yang & Bielawski, 2000).

The d*N*/d*S* ratios indicate that purifying selection is strong in the slow-evolving genes, whereas it is more relaxed but not fully missing in the fast-evolving genes (Table 1; see Table S1B for calculation of d*N*/d*S* ratio). Thus, the protein structure and function may tolerate a higher number of amino acid changes in the fast-evolving genes. However, because the d*N*/d*S* ratios were <1 in the fast-evolving genes, it indicates that some purifying selection still remains in effect, probably preventing pseudogenization and preserving some functionality.

There are two exceptions to these general trends. First, the fast-evolving genes, SPS18 and CTL1, have d*N*/d*S* ratios that approach or exceed unity: 0.8 and 1.3, respectively. These high d*N*/d*S* ratios are correlated to high amino acid divergence as shown in Fig. S1. SPS18 and CTL1 also display conservation in key active sites (all five zinc finger residues in SPS18 and 14 out of 15 catalytically important sites in CTL1 are conserved); yet both genes diverge greatly in areas outside these regions (see S7 and S11). The second exception is in the gene pairs CDC19/PYK2 and ADH1/ADH5; the slow-evolving gene has a higher d*N*/d*S* ratio than the fast-evolving gene. In both cases, the origin of higher d*N*/d*S* ratios in the two slow-evolving genes is that the synonymous substitution rates (d*S*) are markedly lower than they are in the other genes in our study (0.4 and 0.5, respectively, vs. an average of 1.4±0.26 for the other genes, Table S1B).

With the exception of CDC19 and ADH1, a linear correlation (*P*<0.00005) was observed between the d*N*/d*S* ratio and the amino acid substitution rate (d*N*) for the 15 gene pairs (Fig. S1). In this correlation, a higher amino acid substitution rate implies a higher d*N*/d*S* ratio. This may indicate that the higher amino acid substitution rates are caused by more relaxed selection constraints. Positive selection may also play some role, although its detection would require further study. A similarly strong correlation, either positive or negative, was not observed between d*N*/d*S* and d*S*.

### Insertions and deletions

Insertions and deletions (indels) significantly affect the structure of genomes and genes. Not surprisingly, protein structural cores are less tolerant to indels than loops (Taylor & Raes, 2004). In this study, for instance, indels accumulate mostly in predicted (or observed) turn/loop regions (Table S1A). In general, insertions and deletions do not always occur symmetrically. For example, in a study of human pseudogenes, it was observed that deletions are 2.9 times more common than insertions (Zhang & Gerstein, 2003), and in rats there is a 70% excess of deletions over insertions in coding sequences (Taylor & Raes, 2004). By contrast, insertions were found to occur more frequently than deletions in the *cis*-regulatory modules of *Drosophila* (Sinha & Siggia, 2005; Kim & Sinha, 2007).

In this study, sequence comparisons showed that the fast-evolving genes have accumulated nearly equal numbers of total insertions (30) and deletions (29), but six times more total indels than their slower evolving partners (59 vs. 10; see Table 3). Two-thirds of these 69 combined intragenic (i.e. excluding terminal length variation) indels were only one to three amino acids long. However of the 23 longer indels, eight were extensive deletions, removing 10–50 amino acids. In fact, all 14 indels longer than seven amino acids were deletions. Consequently, although intragenic deletions and insertions occurred equally often, deletions removed threefold more amino acid residues than insertions added (Table 3). In addition, five fast-evolving genes have long (*c*. 30 amino acids or longer) terminal deletions when compared with both slow-evolving genes and the *K. waltii* genes (RNR4, CTL1, YKL161C, SPS18, and ELO1). Only HFA1 has a similarly long insertion (75 amino acids), which is located at the protein N-terminus. In other cases, the length variation at the protein termini is <10 amino acids, except that one *K. waltii* gene is 43 amino acids longer and one is *c*. 70 amino acids shorter than the corresponding *S. cerevisiae* genes (PST2/RFS1 and SEC14/SFH1, respectively).

The combined effect of indels and terminal deletions (or insertions) is that the fast-evolvers are on average 5% shorter than the slow-evolvers. However, only seven out of 15 of the fast-evolving genes are shorter than their ohnologous partners (Table 1). But because the shortened fast-evolvers are on average 18% (±6% s.e.) shorter than their partners, while the lengthened fast-evolvers are only 1.8% longer (±0.6% s.e.), the average length of the fast-evolvers is still shorter than the slow-evolvers.

The higher accumulation rate of long indels in the fast-evolving genes may be an indication that they have experienced weaker purifying selection. Conceivably, these indels can be the agents of adaptive changes, but it is also possible that they disrupt enzymatic functions and interactions with small ligands, cofactors and other macromolecules. Disruption of function is even more likely in instances of extreme length reduction, such as in the case of the fast-evolver, *CTL1*, which is reduced in length by 42% relative to the slow-evolving *CET1*. Indeed, as shown below, the extent of length reduction is correlated to losses in protein function.

### Divergence and reduction in functional patterns

In order to determine the differences between fast- and slow-evolving gene products at the functional level, we analyzed their known active sites and cofactor-binding sites by sequence comparison and structural modeling. Structural analysis was only possible when there was enough sequence identity to previously crystallized homologues or when crystal structures were determined for the yeast proteins themselves. This analysis also required functional information from the literature about the active site or sites, or it required that a cofactor, a substrate, or a substrate analog be visible in the crystal structure. In addition, we analyzed changes outside the active sites that could cause functional differences between the fast- and slow-evolving genes. For example, in some genes a large shift in pI may indicate a possible functional change, because the electrostatic interactions with substrates and binding partners could be radically altered.

Table 4 summarizes the results of this functional analysis (the literature information used in this analysis is reported in detail in the Supporting Information). A general trend is that the fast-evolving ohnologs have retained at least one key function and have lost other functions due to mutations. The sequence analysis and modeling studies showed that known or putative binding sites and active sites in the fast-evolving genes differ from those in the corresponding *K. waltii* genes to a greater extent than those in the slow-evolving genes. In other words, the fast-evolving genes have accumulated changes that are likely to significantly affect the functional properties or to completely inactivate a function. This pattern was observed in most of the gene pairs that were analyzed.

**Table 4:** Divergence of the active sites and binding sites in the duplicated gene pairs

Gene pair	Gene function (***f***)	***f***^change*^	Comments (for literature references see the main text and the Supporting information)
*UGP1YHL012W*	UDP-glucose pyrophosphorylase Unknown function	A B A BC?	The potential active site and the glucose-1-phosphate and PPi-binding sites of *YHL012W* contain numerous changes when compared with *UGP1*, the *K. waltii* gene, and the gene family. For example, there is an arginine in place of a potentially catalytic lysine.
*PST2RFS1*	Both flavodoxin-fold proteins with chromatin association	A B A′ B′	A partially modeled flavin mononucleotide (FMN)-binding pocket is conserved in *P. aeruginosa* wrba (1zwl), *K. waltii* 23042, and *PST2* (1 out of nine sites differing). In *RFS1*, four sites out of nine differ. In particular, two potential hydrogen bonds to FMN are missing in *RFS1* (positions G124 and I126). Despite this difference, *PST2* and *RFS1* appear to have overlapping, partially redundant functions.
*MCK1YGK3*	GSK-3 homolog GSK-3 homolog, diverged substrate specificity?	A B A^*^B	*MCK1* is a *GSK-3* homolog Ser/Thr/Tyr kinase. An ADP-binding pocket is well conserved in *YGK3*, whereas a potential substrate-binding surface largely differs from the gene family. A sulfate-binding site is destroyed in *YGK3*. A tyrosine, which is often phosphorylated in this gene family, is also found in *YGK3*.
*ACC1HFA1*	Both acetyl-CoA-carboxylases	A B′ A B′	In comparison with *K. waltii* 6157 and *ACC1*, three out of 16 positions lining the acetyl-CoA-binding pocket have changed in *HFA1*. Another major feature is that *ACC1* is cytoplasmic and *HFA1* mitochondrial gene: a mitochondrial localization signal occurs in *HFA1* but not in ACC1.
*RNR2RNR4*	Ribonucleotide reductase Stabilizing component for ribonucleotide reductase	A′B′ A′B^*^C?	*RNR4* stabilizes *RNR2*'s catalytic diiron center in the RNR2/RNR4 heterodimer. The corresponding diiron is inactivated in *RNR4* since three residues needed for iron coordination are changed. The dimer surface is conserved in *RNR4*, though some adaptive changes could exist. The heterodimer is dominant over the homodimer.
*CET1CTL1*	RNA triphosphatase RNA degradation and processing?	A B A B C?	*CET1* is an RNA triphosphatase and functions in mRNA cap formation. Only 1 out of 15 catalytically important sites are different in *CTL1*. The *CEG1* protein-binding motif (WAQKW) identified in *CET1* is completely missing in *CTL1. CTL1* is severely truncated relative to *CET1. CTL1* might function in RNA degradation or processing.
*VPS21YPT53*	Both *Ypt/Rab* family GTP-binding proteins.	A B A B^*^	The GTP-binding and the protein family sequence features are largely conserved in *YPT53*. The effector-binding loop has experienced divergence in *YPT53*. Mutagenesis of yeast cells has indicated that *YPT53* has a specialized function.
*SEC14SFH1*	PI/PC transfer protein Weak phospholipid transfer?	A B A B^*^C?	SEC14 is a phosphatidylinositol/phosphatidylcholine transfer protein. Functional sites are conserved in *SFH1. SFH1* has lost the ability to function in phospholipid transfer, but still the phospholipid-binding site is conserved indicating a new phospholipid function.
*SLT2YKL161C*	MAP Kinase Kinase with altered substrate specificity?	A B A′B^*^	*SLT2* is a MAPK kinase. Catalytically essential residues and key threonine required for activation are lost in *YKL161C*. The transcription factor, *Rlm1*, is activated by both proteins. *YKL161C* has a new function related to response to oxidative stress. *YKL161C* has conserved ATP-binding and docking sites.
*GCS1SPS18*	ARF-GAP protein Unknown function in sporulation	A B A′B^*^C?	*GCS1* is an *ARF-GAP* protein that functions in vesicular transport. The zinc finger domain is apparently intact in *SPS18* whereas the ARF protein-binding motif is changed. *GCS1* mediates the resumption of cell proliferation from stationary phase. *SPS18* with unknown function is expressed during sporulation.
*CDC19PYK2*	Both pyruvate kinases	A B A B′	*CDC19* is a pyruvate kinase. The crystal structure of *CDC19* has been determined complexed with the allosteric regulator fructose-1,6-biphosphate (FBP) and substrate analog (1a3w). *PYK2* shows functional differences, for example insensitivity to FBP. However, the FBP-binding pocket is conserved.
*ADH1ADH5*	Both alcohol dehydrogenases	A B A′B′	*ADH5* can function as alcohol dehydrogenase, although there are some functional differences.
*GRS1GRS2*	Glycyl tRNA synthetase Defective glycyl tRNA synthetase?	A B A^*^B′ C?	GRS1 is a glycyl-tRNA-synthetase. Unlike *GRS1*, isolated *GRS2* is not stable. Also, *GRS2* has a sequence property that probably affects 3′-end formation. There is also a large deletion near the putative active site of *GRS2*. Experimentally, *GRS2* cannot substitute for *GRS1*.
*ERV14ERV15*	Cargo receptor cycling between ER and Golgi Similar function to *ERV14* in sporulation	A B A′B′	*ERV14* functions in budding and sporulation; *ERV15* has overlapping function only in sporulation. There is one amino acid difference in *ERV15* in a site important for COPII interaction and there are two unique cysteines close to this site, which could form a disulfide bridge affecting COPII binding. Functional tests revealed functional reduction in *ERV15*.
*FEN1ELO1*	Long chain fatty acid elongase Short chain elongase	A′B A′B^*^	*FEN1* is a fatty acid elongase. *FEN1* elongates palmitoyl-CoA (C16) and stearoyl-CoA (C18) to C22 fatty acids. *ELO1* extends C12-C16 fatty acyl-CoAs to C16-C18 fatty acids.

*f^change^: A and B represent gene functions. Markings (′, ^*^ and −) represent degree of change (e.g. A′, minor change in function A; A^*^, major change in function A; and B, deleted function B). C? represents a possible new function. The distinction between major and minor change is not always clear; some minor changes may prove to be major upon further investigation, and vice versa. See Fig. 4 for more details.

**Fig. 1:**
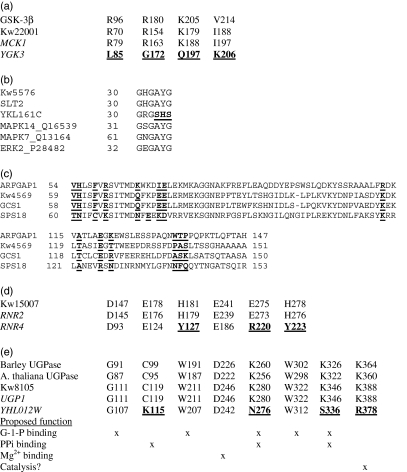
Examples of functional sites, in which the fast-evolving yeast protein has diverged significantly. (a) Sulfate binding site in the mouse GSK-3β and yeast proteins. (b) Phosphate anchor motif GXGXXG in MAP kinases. (c) Binding site (bold and underlined) of *Rattus norvegicus* ARFGAP1 for ADP ribosylation factor ARF1 and the corresponding sites in yeast proteins (*Rattus* sites are from crystal structure; Goldberg, 1999). (d) Conserved iron ligand binding site in diiron center of RNR proteins. (e) Key residues reported to be important for UDP-glucose pyrophosphorylase activity (Geisler *et al.*, 2004). Differing sites in fast-evolving genes are shown in bold and underlined (a–b and d–e).

**Fig. 2:**
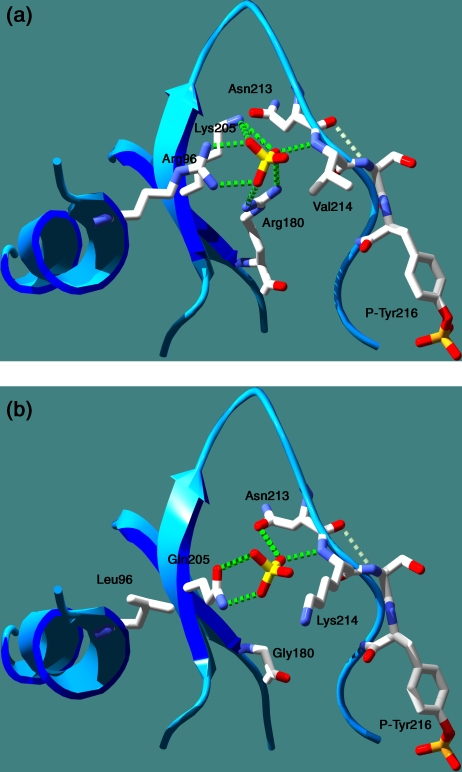
Sulfate-binding site. (a) The sulfate-binding site is shown for the mouse GSK-3β (1gng). Dotted green lines show hydrogen bonding to sulfate. (b) The residues corresponding to the GSK3 sulfate-binding site in YGK3 (see Fig. 1a) were introduced into the 1gng structure in Swiss-PdbViewer (1gng numbering). The side chains of Gln at position 205 (Gln197 in YGK3) and Asn-213 (Asn205 in YGK3) were rotated at some degree to form hydrogen bonds to the sulfate oxygens. Phosphorylated tyrosine (Tyr216 in GSK-3β) is also shown. Pictures were created using Swiss-PdbViewer.

The two glycogen synthase kinase-3 (GSK-3) homologues, MCK1 and YGK3, demonstrate this phenomenon of functional divergence. In the fast-evolving YGK3, a GSK-3-like ADP-binding surface appears to be conserved as does the tyrosine that is phosphorylated, whereas the surface analogous to the binding site for a 39 residue peptide from the C terminus of a protein called FRAT1 does not appear to be conserved (Tables S4A and S4B). This peptide, termed ‘FRATtide’, is known to be bound by GSK-3, and thus the corresponding binding surface in yeast *MCK1* may have a corresponding function (Bax *et al.*, 2001) (see also S4). Moreover, while *MCK1* has an intact sulfate-binding site, like GSK-3, this site is most likely destroyed in *YGK3* (Fig. 1). The sulfate ion functions as a binding site for phosphoserine in the substrates (Bax *et al.*, 2001). In the sulfate ions that have a functional role in proteins, usually every sulfate oxygen is coordinated by two or three hydrogen bonds, and on average an oxyanion (sulfate or phosphate) is held by 7(±3) hydrogen bonds, of which 5(±3) bonds are to protein and the rest are to water molecules (Chakrabarti, 1993; Chertanova & Pascard, 1996). The network of seven hydrogen bonds to protein is seen in the mouse GSK-3β sulfate (see Fig. 2a), whereas the same site in *YGK3* could form four potential hydrogen bonds from three amino acids (see Fig. 2b). In the sulfate-binding site of *GSK-3*β, the mutation of Arg96 to Ala already severely impaired its ability to phosphorylate primed (phosphorylated on serine) substrates (Frame *et al.*, 2001). The presence of all three positively charged amino acids in the sulfate-binding site thus appears to be necessary for the function. Therefore, YGK3 apparently is not able to bind correctly (if at all) the sulfate ion with two negative charges. Moreover, an additional lysine side chain partly covering the sulfate-binding site would probably physically interfere with the functionality in YGK3 (see Fig. 2b). To sum up, *MCK1* is very similar to GSK-3 while *YGK3* appears to have retained some kinase activity but may have its substrate specificity and other functional properties altered. While MCK1 is involved in the control of chromosome segregation and in the regulation of entry into meiosis and other cellular events (Neigeborn & Mitchell, 1991; Shero & Hieter, 1991; Lim *et al.*, 1993; Brazill *et al.*, 1997; Rayner *et al.*, 2002), the role of YGK3 is unclear. Deletion of YGK3 did not show any phenotype effects (Frame *et al.*, 2001).

**Fig. 3:**
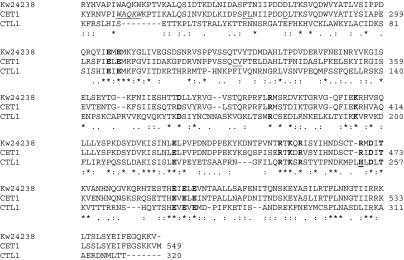
Example of a highly conserved active site in a highly diverged protein. CTL1 is an extremely truncated version of yeast RNA triphosphatase (CET1), which displays only 21% identity in the remaining region. Out of 15 catalytically important residues (shown in bold), only one, histidine (bold and underlined) is different in CTL1 indicating a strong purifying selection in these positions (Lehman *et al.*, 2001). Sites important for dimerization in CET1 are shown underlined (Lehman *et al.*, 2001). Binding site for CEG1 protein (WAQKW) in CET1 is shown in italics and underlined (Ho *et al.*, 1999).

A further example of this limited functional preservation phenomenon is seen in the *CET1*–*CTL1* ohnolog pair (see Fig. 3). *CET1* is a divalent cation-dependent RNA triphosphatase that catalyzes the first step in mRNA cap formation. Bisaillon & Shuman (2001) reported 15 sites that are important for the catalytic activity of *CET1*. Because the fast-evolving ohnolog, *CTL1*, shares all but one of these 15 catalytically important residues, one might expect it to have the same cap formation function. However, *CTL1* has experienced significant changes relative to *CET1*, including an extensive N-terminal deletion (*c*. 210 amino acids), and *CTL1* is only *c*. 21% identical to *CET1* in the remaining region. Moreover, this deletion includes the RNA guanyltransferase (CEG1)-binding motif, WAQKW, which has been identified in *CET1* (Ho *et al.*, 1999). CEG1 interacts with *CET1* and cleaves the β–γ phosphoanhydride bond of 5′-triphosphate RNA to yield a diphosphate end that is then capped with GMP by CEG1. Because *CTL1* has diverged extensively from *CET1*, the high conservation of catalytically important residues is a sign of strong purifying selection in the sites needed for its key function. This also indicates that CTL1 is not becoming a pseudogene. The high d*N*/d*S* ratio of 1.3 may be related mainly to the very rapid and relaxed protein evolution CTL1 is experiencing in regions other than the active site, although the positive selection is not ruled out either. It seems probable that *CTL1* has acquired a specialized function that differs from *CET1*. Indeed, it has been proposed that *CTL1* could have a role in RNA degradation or in processing non-mRNA (Rodriguez *et al.*, 1999). A differing role is also supported by the different locations these proteins have. While *CET1* is located in the nucleus, *CTL1* is found throughout the cell (Rodriguez *et al.*, 1999).

*SLT2*, the slow-evolving ohnologous gene in the *SLT2*/*YKL161C* pair, retains the original function. *YKL161C*, on the other hand, appears to represent a gene that has experienced neofunctionalization after the WGD. *SLT2* is a mitogen-activated protein (MAP) kinase, which has two major targets: one is a transcription factor that activates genes involved in cell wall regulation, while the other set of targets regulates the G1 to S transition (Martin-Yken *et al.*, 2003). *YKL161C* shows significant sequence homology to *SLT2* through its N-terminal 362 amino acids (75% positives or identities). On the other hand, the C-terminal 71 amino acids of *YKL161C* show no similarity to C-terminal 122 amino acids of *SLT2*.

Interestingly, *YKL161C* differs from *SLT2* in its kinase activity and yet overlaps with *SLT2* in other functions, such as in its interaction partners. The key change in kinase activity is the result of point mutations that effectively remove *YKL161C* from the category of known MAP kinases (the divergence in the phosphate anchor motif is shown in Fig. 1), whereas ATP-binding and docking sites are quite conserved in *YKL161C* (see Fig. S10). All MAP kinases are activated by phosphorylation at key threonine and tyrosine residues separated by a single amino acid (Widmann *et al.*, 1999). The position of this TXY motif in *SLT2* is at 190 (Thr) and 192 (Tyr), respectively. In *YKL161C*, the threonine is replaced by a positively charged lysine (Vandenbol *et al.*, 1994). However, Watanabe *et al.* (1997) found that *YKL161C* functions to activate a key transcription factor, Rlm1, which is also activated by *SLT2*. They also showed by site-directed mutagenesis that the tyrosine found in the TXY motif (KXY in *YKL161C*) is critical to its ability to activate *Rlm1*. More recently, *YKL161C* has been found as one of the genes activated by continuous oxidative stress, and its loss results in hypersensitivity to oxidative stress (Belli *et al.*, 2004). Thus, *YKL161C* shows overlap with *SLT2* in substrates. However, it is clearly activated by kinases other than those that activate *SLT2*, and it has a new function in response to oxidative stress that *SLT2* does not have.

The *GCS1* and *SPS18* ohnolog pair is another example of partial retention of function. *GCS1* is a yeast ADP-ribosylation factor GTPase-activating protein (ARFGAP) that functions in the endoplasmic reticulum (ER)–Golgi vesicular transport system (Poon *et al.*, 1996, 1999). ADP-ribosylation factors (ARFs) are members of the Ras superfamily of GTP-binding proteins. The intrinsic GTPase activity of ARFs is low, but it can be activated by ARFGAPs. The zinc finger region that is required for this activation appears to be intact in both the slow-evolving *GCS1* and the fast-evolving *SPS18*, because, in the structural models, the four cysteines of the zinc finger region are located in the correct positions for both *GCS1* and *SPS18* (see S11 and Fig. S11). However, residues corresponding to the ARF-binding sites of rat ARFGAP1 that are well conserved in yeast *GCS1* are completely different in *SPS18* (Fig. 1). Therefore, the fast-evolving *SPS18* has probably retained the original activity of the zinc finger region, but it does not interact with the same ARF protein (if at all) that is activated by *GCS1*.

*VPS21* and *YPT53* belong to the *Ypt*/*Rab* family of membrane-associated GTPases. They are required for transport during endocytosis and for correct sorting of vacuolar hydrolases (Singer-Kruger *et al.*, 1994; Esters *et al.*, 2000). Although *YPT53* has conserved most of the features in *VPS21*, mutagenesis in yeast indicated that *YPT53* has a specialized role in the cell (Singer-Kruger *et al.*, 1994). This is further supported by the fact that a loop in *VPS21* that is important for effector binding differs greatly in *YPT53* (see Fig. S8).

A transmembrane protein, *ERV14*, functions as a cargo receptor cycling between the ER and the Golgi. In the *ERV14*/*ERV15* pair, the *ERV14* protein has retained a larger set of functions; it functions both in budding and in sporulation, whereas *ERV15* functions only in sporulation (Powers & Barlowe, 1998; Nakanishi *et al.*, 2007). The two proteins appear to have partly overlapping functions (Nakanishi *et al.*, 2007), indicating that they may have slightly differing functions (specialization) in sporulation. These data indicate that *ERV15* has a reduced functionality when compared with *ERV14*. A potential protein interaction site has undergone changes in *ERV15* (see Table 4 and S15)

The duplicated pair *FEN1* and *ELO1* may represent a situation in which both proteins have specialized to function with a subset of substrates (Rossler *et al.*, 2003). *FEN1* synthesizes longer fatty acids and *ELO1* synthesizes shorter fatty acids. It appears that both proteins have retained the full original function, and only the substrate specificity has changed, possibly in both proteins. This may increase the total efficiency of fatty acid synthesis. *FEN1* has seven predicted transmembrane domains, and *ELO1* has at least five (maybe even seven) transmembrane domains (see Fig. S16). The retaining of the original function in *ELO1* as fatty acid elongase is probably reflected in the retaining of pI despite significant sequence divergence (see Table 1). The changes in the substrate specificity could have been caused by changes in the fatty acid-binding surface.

### Minor changes

Some gene pairs showed only minor sequence divergence in the functional sites. Even in these cases, the overall protein functions had diverged between the slow- and fast-evolving genes.

*ACC1* and *HFA1* are enzymes involved in the fatty acid synthesis and contain biotin carboxylase (BC) and carboxyltransferase (CT) domains. The major form of divergence is in the localization; *ACC1* is cytoplasmic and *HFA1* is a mitochondrial enzyme. The BT and CT domains in the fast-evolving gene, *HFA1*, are well conserved, although some minor differences occur (Table S5 and Fig. S5A), and the theoretical pI of *HFA1*-CT domain (pI 8.7) is considerably different from the pI of *ACC1*-CT (pI 5.45) (the same does not hold true for the BC domains). According to the d*N*/d*S* ratio, the sequence outside these domains is experiencing a more relaxed divergence in *HFA1* (Table S1B), indicating that the mitochondrial function requires a lower number of conserved protein features than what is required for the cytoplasmic function or the question is about adaptive changes. Importantly, *HFA1* protein missing the signal sequence (targeting the mitochondria) can compensate the deletion of *ACC1* (Hoja *et al.*, 2004).

The pyruvate kinase genes *CDC19* and *PYK2* function in the glycolytic pathway of sugar metabolism (Pearce *et al.*, 2001; Portela *et al.*, 2002). *CDC19* is tightly regulated and activated by fructose-1,6-bisphosphate (FBP). *PYK2* transcription is repressed by glucose and it is active without FBP (Boles *et al.*, 1997; Portela *et al.*, 2002). There are minor differences in the FBP-binding site, active site, and dimerization site between *PYK2* and *CDC19*. It is not yet clear how the observed differences in these sites are involved in the functional divergence.

Alcohol dehydrogenase is required for the reduction of acetaldehyde to ethanol, which is the last step in the glycolytic pathway. Yeast has several alcohol dehydrogenase genes: *ADH1, ADH2, ADH3*, and *ADH5* form a highly similar group of genes (Feldmann *et al.*, 1994; Leskovac *et al.*, 2002). *ADH1* and *ADH5* form the ohnolog pair derived from WGD. *ADH1* is the major enzyme functioning as alcohol dehydrogenase. Mutation tests indicate that *ADH5* protein is also able to produce ethanol in yeast (Dickinson *et al.*, 2003; Smith *et al.*, 2004). A new role of *ADH5* is indicated by the finding that its expression is increased in the *S. cerevisiae* mutant able to grow anaerobically on xylose (Sonderegger *et al.*, 2004). However, NAD-, zinc-, and substrate-binding sites appear to be fully or largely conserved (Table S13 and Fig. S13).

*PST2* and *RFS1* are flavodoxin-fold proteins and have a overlapping, partially redundant function in DNA repair (Valencia-Burton *et al.*, 2006). There are conflicting results about the localization (see S3). *PST2* and *RFS1* have been localized to the cytoplasm (Huh *et al.*, 2003), but there is also a report about association with chromatin (Valencia-Burton *et al.*, 2006). The divergence of functions may be reflected in the differences in the flavin mononucleotide (FMN)-binding pocket (Table S3), in which *RFS1* has lost two potential hydrogen bonds binding to FMN (see Supporting Information and Table S3), and also reflected in differing localization predictions (Table 2).

### Divergence in localization

In some cases, new localization patterns have evolved in the duplicated genes (Table 2). For example, *ACC1* has lost its mitochondrial localization signal, whereas *HFA1* retained this signal, which is located upstream from the first methionine (Hoja *et al.*, 2004), and localizes the protein to the mitochondria. *HFA1* appears to have a non-AUG translation signal and thus its expression level is low (Hoja *et al.*, 2004). The yeasts that have only one gene (e.g. *K. waltii*), presumably express the cytoplasmic and mitochondrial proteins from a single gene by starting the protein expression at two different sites. In *Kluyveromyces lactis* acetyl-CoA-carboxylase gene, the upstream sequence before the first methionine, when translated to protein also contains a putative mitochondrial-targeting signal (see S5). In *S. cerevisiae*, the WGD event allowed specialization of the genes to mitochondrial and cytoplasmic forms.

Novel localization patterns could be predicted from sequence information (Table 2). We used this approach to analyze how often the localization pattern differs for the fast-evolving protein. Some examples are discussed here. For example, a nuclear localization signal (although weak) was predicted for the fast-evolving *SFH1* gene using the Yeast Protein Localization Server. *SFH1* is localized to the nucleus (Huh *et al.*, 2003), although a cytoplasmic localization has also been observed (Huh *et al.*, 2003). A cytoplasmic localization was predicted and observed for its slowly evolving partner, *SEC14* (Schnabl *et al.*, 2003), although a nuclear localization has also been observed (Huh *et al.*, 2003). Despite some uncertainty in the localization, the differing localization predictions tend to indicate differing roles.

The divergence in localization appears to be evident in *CET1* and *CTL1. CET1* is known to be localized to the nucleus (Itoh *et al.*, 1987). The nuclear localization was also predicted from the amino acid sequence. On the other hand, the much shorter ohnolog, *CTL1*, is expressed both in the nucleus and in the cytoplasm (Rodriguez *et al.*, 1999), and weak nuclear and mitochondrial localization signals were predicted for this protein (see also S7). *GCS1* is predicted to be cytoplasmic, which is in line with the finding that *GCS1* functions in the ER–Golgi vesicular transport system (Poon *et al.*, 1996, 1999). The ohnolog pair of *GCS1*, which is *SPS18*, is predicted to be nuclear protein (no experimental localization data), which indicates a fully different function, especially because SPS18 has experienced functional changes.

Predictions were not always correct. For example, a mitochondrial location was predicted for *MCK1*. Because *MCK1* has a role for example in chromosome segregation and regulation of other nuclear events (Neigeborn & Mitchell, 1991; Shero & Hieter, 1991; Lim *et al.*, 1993; Brazill *et al.*, 1997; Rayner *et al.*, 2002), it appears that the mitochondrial localization is not a correct prediction. Huh *et al.* (2003) reported both cytoplasmic and nuclear localization for *MCK1*. Predicted localization for *GRS1* is cytoplasmic; the protein is localized both to the cytoplasm and to the mitochondria (Turner *et al.*, 2000). Predicted localization for *GRS2* is nuclear, which could indicate the potential of an evolving functional divergence, although the protein appears to be cytoplasmic (Turner *et al.*, 2000). There are also other differing predictions (Table 2). Although caution is needed in interpreting the localization predictions, the fact that different localization predictions are made for the fast- and slow-evolving genes indicates that there is much potential in evolving divergence in the actual localizations. Thus, change in localization could be an adaptation acquired quite easily towards attaining a divergent functional role.

### Fully new functions?

An extraordinary case of functional specialization is found in *RNR2* and *RNR4. RNR2* and *RNR4* correspond to the R2 subunit of eukaryotic class I ribonucleotide reductases (RNR). An RNR is formed of R1 and R2 subunits: R1 contains substrate and allosteric effector-binding sites and R2 contains a catalytically essential diirontyrosyl radical cofactor. The active form of R2 is usually a homodimer, whereas in yeast the heterodimer of *RNR2* and *RNR4* is the predominant form Sommerhalter *et al.* (2004). Structural differences between the heterodimers and typical homodimers in *S. cerevisiae* are reported by Sommerhalter *et al.* (2004). It was found that the *RNR4* protein lacks six out of the 16 residues that are conserved in most R2 proteins (Voegtli *et al.*, 2001) including three residues involved in coordinating iron (Fig. 1). Consequently, *RNR4* cannot accommodate a diiron center. However, *RNR4* is required to activate *RNR2*, which includes stabilization of the diiron center in *RNR2*. It appears that the yeast RNR has evolved to function optimally with only one catalytically essential diirontyrosyl radical cofactor per dimer (Sommerhalter *et al.*, 2004). At the same time, *RNR4* has experienced numerous amino acid changes, some of them probably being adaptive (better heterodimer formation) and some of them having inactivated other functions (diiron center). *RNR2* may also have suffered functionally from mutations, because the heterodimer with *RNR4* is needed for the optimal activity. *RNR2* and *RNR4* appear to represent both subfunctionalization and neofunctionalization.

The fast-evolving *YHL012W* represents a case in which the putative active site has experienced such extensive changes that it is likely that activity is fully abolished or completely different from the UDP-glucose pyrophosphorylase activity, which remains in the slowly evolving *UGP1* (see Table S2B). The function of *YHL012W* is unknown. The key residues important for UDP-glucose pyrophosphorylase activity have been identified in barley (Geisler *et al.*, 2004). These sites are conserved in *UGP1* and the corresponding *K. waltii* gene (Fig. 1). On the other hand, *YHL012W* contains several differing positions (four out of eight), indicating that its function is largely changed or its active site is not functional. Interestingly, the d*N*/d*S* ratio (0.52) indicates that a weak purifying selection may still be in effect with the *YHL012W* gene.

In the *SEC14*/*SFH1* duplicated gene pair, *SFH1* is not able to control phosphatidylcholine degradation, which is the function of *SEC14* (Schnabl *et al.*, 2003). In fact, *SFH1* is neither a phosphatidylinositol nor a phosphatidylcholine transfer protein *in vitro* (Li *et al.*, 2000). When overexpressed, it complements the *SEC14*-related functions only to a very limited degree (Griac *et al.*, 2006). Another reason for the weak growth complementation of *SEC14* deficiency could be that *SFH1* is localized predominantly to the nucleus and *SEC14* is predominantly a cytosolic protein.

Despite all these differences, *SFH1* conserves all recognized critical structural motifs of *SEC14* (Sha *et al.*, 1998). We also found only conservation in the functionally important sites. A difference in localization prediction was observed (Table 2). In addition to this divergence in localization, the high sequence divergence between *SFH1* and *SEC14* (64% identity) allows the accumulation of minor changes in many sites that, together, appear to affect the functionality of *SFH1* profoundly. Thus, based on the analysis of functionally important residues, it appears that much is conserved in *SFH1*; yet, due to the vast changes in other residues *SFH1* may have evolved a new functional role such as one that involves the binding of phospholipids.

We cannot rule out the possibility that some of the fast-evolving genes would be on the way to becoming pseudogenes. For example, the *GRS2* protein, which forms an ohnolog pair with *GRS1*, has been reported to be expressed in low amounts and to not be stable when purified (Turner *et al.*, 2000). A loss of functional properties can be seen in the *GRS2* sequence (see S14). But even in this case, the d*N*/d*S* ratio (0.33) indicates that *GRS2* could be experiencing some purifying selection, and thus may have a specialized role in the yeast cell. Indeed, because vast majority of the 5000 duplicated genes have been lost in *S. cerevisiae*, it is likely that all those (or most) that are left (*c*. 500) have survived because they have a specialized role or because a higher gene dosage favors their survival. More information is needed to estimate how often a completely novel function has been acquired.

It appears that *RNR4* and possibly also *YHL012W* have adopted a role in yeast that is not dependent on the primary activity of the ancestral protein – the activity that is still seen in the slowly evolving duplicate. For example, a protein–protein interaction without any enzymatic activity could create a novel specialized role for a duplicated gene, as is the case for *RNR4* in its obligate heterodimer with *RNR2*. A need for such a role for a duplicated gene could have arisen from a harmful mutation in another protein, whose effect was then mitigated by a compensating protein–protein interaction.

## Discussion

By examining 15 of the most asymmetric ohnologs from the recently enumerated set of *c*. 500 yeast gene duplicates (Kellis *et al.*, 2004), we have uncovered several qualitative trends concerning the evolution of duplicated genes. Although our sample size (30) is small and an exhaustive, comprehensive approach would involve defining the structure–function relationships in most of *c*. 500 ohnologs, our study reveals some interesting trends, whose significance arises from the fact that these 15 gene pairs comprise the fastest-diverging subset. The picture that emerges is one in which selection pressure is partially relaxed and evolution speed is increased for the fast-evolving partner in each ohnolog. This allows functional divergence of the fast-evolving partner. Typically, its functional divergence includes the acquisition of a novel role in the cell, which occurs often in concert with – and most likely as a consequence of – a reduction in its number of subfunctions. Its newly acquired role in the cell tends to occur in a more limited range of cellular importance when compared with the slow-evolving partner. Moreover, its novel role is mostly based on a retained ancestral function or subfunction, whose regulation, specific protein activity, or protein localization has been modified; although it is possible in a few cases that the ancestral function itself is not even retained. Finally, we must consider the possibility that the slowly evolving partners could themselves have experienced a minor reduction in their number of subfunctions or, conversely, that some fast-evolving genes have not experienced any major reduction in their functional pattern even while their cellular roles have slightly changed. Indeed, we might expect that these more subtle alternatives are a common mode of divergence in the whole group of *c*. 500 ohnologs.

**Fig. 4:**
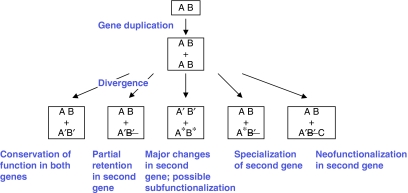
Schematic presentation of possible divergence modes. The ancestral protein with subfunctions A and B was duplicated in the WGD, and this figure shows schematically how the functional divergence has led to many types of changes in the diverging gene pair. Reduction of functions is common among the fast-evolving genes in the group of 15 gene pairs. Fast-evolving genes have also adopted new roles in the yeast cell. A′B′ minor changes in the subfunctions; A^*^, B^*^, novel functional properties (e.g. changes in location, interaction with substrate, or protein–protein interactions); (with strikethrough), A′B′ deletion of subfunctions; and C, completely new (sub)function.

In principle, there could be a situation in which two functions of an ancestral gene are split evenly between the two ohnologous genes. However, the major trend, based on the functions that could be identified in our study, is that one gene retains the original, or nearly original, set of subfunctions while the other gene displays a reduced number of subfunctions. Essentially, the distribution of the original set of subfunctions between the genes is asymmetric. It could be that among the *c*. 500 ohnologs, this strong functional specialization occurs only in the fastest-diverging genes, such as in those that we examined. However, it has been proposed that catalytically inactive enzyme-homologues occur widely and are involved in regulatory processes (Pils & Schultz, 2004). It is possible to see such a development occuring in yeast among the fast-evolving genes. Altogether, already a set of 15 duplicated gene pairs reveals a quite wide variation in the functional patterns of how new adapted protein roles may appear (see Fig. 4, which schematically shows the variety of divergence patterns observed in yeast). Often the deletion of the fast-evolving gene is slightly harmful, which could mean that adaptation of the new role has increased the fitness or alternatively compensated a harmful mutation in some other protein. An adaptational role is indicated for example for fast-evolving genes PYK2 and ADH5 that are expressed in anaerobic growth when xylose is the growth substrate, which is not normally utilized by yeast in the absence of oxygen (Sonderegger *et al.*, 2004). In a recent study, Conant & Wolfe (2007) proposed that fixation of a WGD was favorable for the increased glucose metabolism. Adaptational innovations among the duplicated genes might also be useful in searching the sequence space for finding biotechnologically relevant enzyme variants (Leisola & Turunen, 2007).

Based on our results and the known functional information on many ohnolog gene pairs, there appears to be a trend that the complexity of the genes (amount of functions in one gene) is slowly decreasing due to gene duplication and subsequent divergence. Functional reduction of the fast-evolving genes in the duplicated gene pairs is also seen in the finding that they have less protein–protein interactions (Langkjaer *et al.*, 2003; Kim & Yi, 2006). A large functional modification and evolution of a novel function or a new role in the cell appears to go through degeneration, in which a limited functional role keeps the gene alive in the initial stages, thereby allowing an increased evolution rate. Further studies are required to determine how often this kind of evolutionary mode occurs among duplicated yeast genes. It is possible that only a very small fraction of gene duplicates experiences a significant functional divergence (Lynch & Conery, 2000; Wapinski *et al.*, 2007). More functional information about the corresponding *K. waltii* proteins is also needed in order to evaluate more precisely how much the slowly evolving *S. cerevisiae* proteins have diverged from *K. waltii* after the WGD event. Relaxation of functional constraints and subfunctionalization after WGD is a larger phenomenon, for example, as reported for pseudotetraploid frog *Xenopus laevis* in a study comparing over 2000 gene triplets in *X. laevis* and *Xenopus tropicalis* (Hellsten *et al.*, 2007). Consequently, we expect that examination of the divergence at the individual protein level in large quantities will gradually reveal a much wider diversity in the protein functional divergence patterns than currently known.

## Statement

Reuse of this article is permitted in accordance with the Creative Commons Deed, Attribution 2.5, which does not permit commercial exploitation.

## Supporting Information

**Appendix S1.** Supplemental data for duplicated *Saccharomyces cerevisiae* gene pairs.

## References

[b1] Bax B, Carter PS, Lewis C (2001). The structure of phosphorylated GSK-3beta complexed with a peptide, FRATtide, that inhibits beta-catenin phosphorylation. Structure.

[b2] Belli G, Molina MM, Garcia-Martinez J, Perez-Ortin JE, Herrero E (2004). *Saccharomyces cerevisiae* glutaredoxin 5-deficient cells subjected to continuous oxidizing conditions are affected in the expression of specific sets of genes. J Biol Chem.

[b3] Bisaillon M, Shuman S (2001). Structure-function analysis of the active site tunnel of yeast RNA triphosphatase. J Biol Chem.

[b4] Boles E, Schulte F, Miosga T (1997). Characterization of a glucose-repressed pyruvate kinase (Pyk2p) in *Saccharomyces cerevisiae* that is catalytically insensitive to fructose-1,6-bisphosphate. J Bacteriol.

[b5] Brazill DT, Thorner J, Martin GS (1997). Mck1, a member of the glycogen synthase kinase 3 family of protein kinases, is a negative regulator of pyruvate kinase in the yeast *Saccharomyces cerevisiae*. J Bacteriol.

[b6] Byrne KP, Wolfe KH (2007). Consistent patterns of rate asymmetry and gene loss indicate widespread neofunctionalization of yeast genes after whole-genome duplication. Genetics.

[b7] Chakrabarti P (1993). Anion binding sites in protein structures. J Mol Biol.

[b8] Chertanova L, Pascard C (1996). Statistical analysis of noncovalent interactions of anion groups in crystal structures. I. Hydrogen bonding of sulfate anions. Acta Crystallographica Section B.

[b9] Conant GC, Wagner A (2003). Asymmetric sequence divergence of duplicate genes. Genome Res.

[b10] Conant GC, Wolfe KH (2006). Functional partitioning of yeast co-expression networks after genome duplication. PLoS Biol.

[b11] Conant GC, Wolfe KH (2007). Increased glycolytic flux as an outcome of whole-genome duplication in yeast. Mol Syst Biol.

[b12] Dickinson JR, Salgado LE, Hewlins MJ (2003). The catabolism of amino acids to long chain and complex alcohols in *Saccharomyces cerevisiae*. J Biol Chem.

[b13] Dietrich FS, Voegeli S, Brachat S (2004). The *Ashbya gossypii* genome as a tool for mapping the ancient *Saccharomyces cerevisiae* genome. Science.

[b14] Drummond DA, Bloom JD, Adami C, Wilke CO, Arnold FH (2005). Why highly expressed proteins evolve slowly. Proc Natl Acad Sci USA.

[b15] Esters H, Alexandrov K, Constantinescu AT, Goody RS, Scheidig AJ (2000). High-resolution crystal structure of *S. cerevisiae* Ypt51(DeltaC15)-GppNHp, a small GTP-binding protein involved in regulation of endocytosis. J Mol Biol.

[b16] Feldmann H, Aigle M, Aljinovic G (1994). Complete DNA sequence of yeast chromosome II. EMBO J.

[b17] Frame S, Cohen P, Biondi RM (2001). A common phosphate binding site explains the unique substrate specificity of GSK3 and its inactivation by phosphorylation. Mol Cell.

[b18] Geisler M, Wilczynska M, Karpinski S, Kleczkowski LA (2004). Toward a blueprint for UDP-glucose pyrophosphorylase structure/function properties: homology-modeling analyses. Plant Mol Biol.

[b19] Goldberg J (1999). Structural and functional analysis of the ARF1-ARFGAP complex reveals a role for coatomer in GTP hydrolysis. Cell.

[b20] Griac P, Holic R, Tahotna D (2006). Phosphatidylinositol-transfer protein and its homologues in yeast. Biochem Soc Trans.

[b21] He X, Zhang J (2005). Rapid subfunctionalization accompanied by prolonged and substantial neofunctionalization in duplicate gene evolution. Genetics.

[b22] Hellsten U, Khokha MK, Grammer TC, Harland RM, Richardson P, Rokhsar DS (2007). Accelerated gene evolution and subfunctionalization in the pseudotetraploid frog *Xenopus laevis*. BMC Biol.

[b23] Ho CK, Lehman K, Shuman S (1999). An essential surface motif (WAQKW) of yeast RNA triphosphatase mediates formation of the mRNA capping enzyme complex with RNA guanylyltransferase. Nucleic Acids Res.

[b24] Hoja U, Marthol S, Hofmann J (2004). HFA1 encoding an organelle-specific acetyl-CoA carboxylase controls mitochondrial fatty acid synthesis in *Saccharomyces cerevisiae*. J Biol Chem.

[b25] Hughes AL, Friedman R (2005). Variation in the pattern of synonymous and nonsynonymous difference between two fungal genomes. Mol Biol Evol.

[b26] Huh WK, Falvo JV, Gerke LC, Carroll AS, Howson RW, Weissman JS, O'Shea EK (2003). Global analysis of protein localization in budding yeast. Nature.

[b27] Itoh N, Yamada H, Kaziro Y, Mizumoto K (1987). Messenger RNA guanylyltransferase from *Saccharomyces cerevisiae*. Large scale purification, subunit functions, and subcellular localization. J Biol Chem.

[b28] Kellis M, Birren BW, Lander ES (2004). Proof and evolutionary analysis of ancient genome duplication in the yeast *Saccharomyces cerevisiae*. Nature.

[b29] Kim J, Sinha S (2007). Indelign: a probabilistic framework for annotation of insertions and deletions in a multiple alignment. Bioinformatics.

[b30] Kim S-H, Yi SV (2006). Correlated Asymmetry of Sequence and Functional Divergence Between Duplicate Proteins of *Saccharomyces cerevisiae* [Article]. Mol Biol Evol.

[b31] Kimura M (1983). The Neutral Theory of Molecular Evolution.

[b32] Kumar S, Tamura K, Nei M (2004). MEGA3: integrated software for molecular evolutionary genetics analysis and sequence alignment. Brief Bioinform.

[b33] Kurtzman CP (2003). Phylogenetic circumscription of *Saccharomyces, Kluyveromyces* and other members of the *Saccharomycetaceae*, and the proposal of the new genera *Lachancea, Nakaseomyces, Naumovia, Vanderwaltozyma* and *Zygotorulaspora*. FEMS Yeast Res.

[b34] Langkjaer RB, Cliften PF, Johnston M, Piskur J (2003). Yeast genome duplication was followed by asynchronous differentiation of duplicated genes. Nature.

[b35] Lehman K, Ho CK, Shuman S (2001). Importance of homodimerization for the *in vivo* function of yeast RNA triphosphatase. J Biol Chem.

[b36] Leisola M, Turunen O (2007). Protein engineering: opportunities and challenges. Appl Microbiol Biot.

[b37] Leskovac V, Trivic S, Pericin D (2002). The three zinc-containing alcohol dehydrogenases from baker's yeast, *Saccharomyces cerevisiae*. FEMS Yeast Res.

[b38] Li X, Routt SM, Xie Z (2000). Identification of a novel family of nonclassic yeast phosphatidylinositol transfer proteins whose function modulates phospholipase D activity and Sec14p-independent cell growth. Mol Biol Cell.

[b39] Lim MY, Dailey D, Martin GS, Thorner J (1993). Yeast MCK1 protein kinase autophosphorylates at tyrosine and serine but phosphorylates exogenous substrates at serine and threonine. J Biol Chem.

[b40] Lynch M, Conery JS (2000). The evolutionary fate and consequences of duplicate genes. Science.

[b41] Malmstrom L, Riffle M, Strauss CE, Chivian D, Davis TN, Bonneau R, Baker D (2007). Superfamily assignments for the yeast proteome through integration of structure prediction with the gene ontology. PLoS Biol.

[b42] Martin-Yken H, Dagkessamanskaia A, Basmaji F, Lagorce A, Francois J (2003). The interaction of Slt2 MAP kinase with Knr4 is necessary for signalling through the cell wall integrity pathway in *Saccharomyces cerevisiae*. Mol Microbiol.

[b43] Nakanishi H, Suda Y, Neiman AM (2007). Erv14 family cargo receptors are necessary for ER exit during sporulation in *Saccharomyces cerevisiae*. J Cell Sci.

[b44] Neigeborn L, Mitchell AP (1991). The yeast MCK1 gene encodes a protein kinase homolog that activates early meiotic gene expression. Genes Dev.

[b45] Ohno S (1970). Evolution by Gene Duplication.

[b46] Pearce AK, Crimmins K, Groussac E (2001). Pyruvate kinase (Pyk1) levels influence both the rate and direction of carbon flux in yeast under fermentative conditions. Microbiology.

[b47] Pils B, Schultz J (2004). Inactive enzyme-homologues find new function in regulatory processes. J Mol Biol.

[b48] Poon PP, Wang X, Rotman M (1996). *Saccharomyces cerevisiae* Gcs1 is an ADP-ribosylation factor GTPase-activating protein. Proc Natl Acad Sci USA.

[b49] Poon PP, Cassel D, Spang A, Rotman M, Pick E, Singer RA, Johnston GC (1999). Retrograde transport from the yeast Golgi is mediated by two ARF GAP proteins with overlapping function. EMBO J.

[b50] Portela P, Howell S, Moreno S, Rossi S (2002). *In vivo* and *in vitro* phosphorylation of two isoforms of yeast pyruvate kinase by protein kinase A. J Biol Chem.

[b51] Powers J, Barlowe C (1998). Transport of axl2p depends on erv14p, an ER-vesicle protein related to the *Drosophila cornichon* gene product. J Cell Biol.

[b52] Rayner TF, Gray JV, Thorner JW (2002). Direct and novel regulation of cAMP-dependent protein kinase by Mck1p, a yeast glycogen synthase kinase-3. J Biol Chem.

[b53] Rodriguez CR, Takagi T, Cho EJ, Buratowski S (1999). A *Saccharomyces cerevisiae* RNA 5′-triphosphatase related to mRNA capping enzyme. Nucleic Acids Res.

[b54] Rossler H, Rieck C, Delong T, Hoja U, Schweizer E (2003). Functional differentiation and selective inactivation of multiple *Saccharomyces cerevisiae* genes involved in very-long-chain fatty acid synthesis. Mol Genet Genomics.

[b55] Schnabl M, Oskolkova OV, Holic R (2003). Subcellular localization of yeast Sec14 homologues and their involvement in regulation of phospholipid turnover. Eur J Biochem.

[b56] Schwede T, Kopp J, Guex N, Peitsch MC (2003). SWISS-MODEL: an automated protein homology-modeling server. Nucleic Acids Res.

[b57] Sha B, Phillips SE, Bankaitis VA, Luo M (1998). Crystal structure of the *Saccharomyces cerevisiae* phosphatidylinositol-transfer protein. Nature.

[b58] Shero JH, Hieter P (1991). A suppressor of a centromere DNA mutation encodes a putative protein kinase (MCK1). Genes Dev.

[b59] Singer-Kruger B, Stenmark H, Dusterhoft A, Philippsen P, Yoo JS, Gallwitz D, Zerial M (1994). Role of three rab5-like GTPases, Ypt51p, Ypt52p, and Ypt53p, in the endocytic and vacuolar protein sorting pathways of yeast. J Cell Biol.

[b60] Sinha S, Siggia ED (2005). Sequence turnover and tandem repeats in *cis*-regulatory modules in drosophila. Mol Biol Evol.

[b61] Smith MG, Des Etages SG, Snyder M (2004). Microbial synergy via an ethanol-triggered pathway. Mol Cell Biol.

[b62] Sommerhalter M, Voegtli WC, Perlstein DL, Ge J, Stubbe J, Rosenzweig AC (2004). Structures of the yeast ribonucleotide reductase Rnr2 and Rnr4 homodimers. Biochemistry.

[b63] Sonderegger M, Jeppsson M, Hahn-Hagerdal B, Sauer U (2004). Molecular basis for anaerobic growth of *Saccharomyces cerevisiae* on xylose, investigated by global gene expression and metabolic flux analysis. Appl Environ Microb.

[b64] Stephens SG (1951). Possible significance of duplication in evolution. Adv Genet.

[b65] Taylor JS, Raes J (2004). Duplication and divergence: the evolution of new genes and old ideas. Annu Rev Genet.

[b66] Tirosh I, Barkai N (2007). Comparative analysis indicates regulatory neofunctionalization of yeast duplicates. Genome Biol.

[b67] Turner RJ, Lovato M, Schimmel P (2000). One of two genes encoding glycyl-tRNA synthetase in *Saccharomyces cerevisiae* provides mitochondrial and cytoplasmic functions. J Biol Chem.

[b68] Valencia-Burton M, Oki M, Johnson J, Seier TA, Kamakaka R, Haber JE (2006). Different mating-type-regulated genes affect the DNA repair defects of *Saccharomyces* RAD51, RAD52 and RAD55 mutants. Genetics.

[b69] Vandenbol M, Bolle PA, Dion C, Portetelle D, Hilger F (1994). Sequencing and analysis of a 20.5 kb DNA segment located on the left arm of yeast chromosome XI. Yeast.

[b70] Voegtli WC, Ge J, Perlstein DL, Stubbe J, Rosenzweig AC (2001). Structure of the yeast ribonucleotide reductase Y2Y4 heterodimer. Proc Natl Acad Sci USA.

[b71] Wagner A (2002). Estimating coarse gene network structure from large-scale gene perturbation data. Genome Res.

[b72] Wapinski I, Pfeffer A, Friedman N, Regev A (2007). Natural history and evolutionary principles of gene duplication in fungi. Nature.

[b73] Watanabe N, Madaule P, Reid T (1997). p140mDia, a mammalian homolog of *Drosophila diaphanous*, is a target protein for Rho small GTPase and is a ligand for profilin. EMBO J.

[b74] Widmann C, Gibson S, Jarpe MB, Johnson GL (1999). Mitogen-activated protein kinase: conservation of a three-kinase module from yeast to human. Physiol Rev.

[b75] Wolfe K (2004). Evolutionary genomics: yeasts accelerate beyond BLAST. Curr Biol.

[b76] Yang Z, Bielawski JP (2000). Statistical methods for detecting molecular adaptation. Trends Ecol Evol.

[b77] Zhang Z, Gerstein M (2003). Patterns of nucleotide substitution, insertion and deletion in the human genome inferred from pseudogenes. Nucleic Acids Res.

