# Supplementary material for: In silico evidence for functional specialization after genome duplication in yeast
Source: FEMS Yeast Res. 2008 Nov 3;9(1):16–31. doi: 10.1111/j.1567-1364.2008.00451.x (PMC2704937; doi:10.1111/j.1567-1364.2008.00451.x)
Supplement: Supplementary file 1 [file fyr0009-0016-SD1.doc]

**SUPPLEMENTAL DATA FOR DUPLICATED *SACCHAROMYCES CEREVISIAE* GENE PAIRS**

Ossi Turunen^1^, Ralph Seelke^2^ and Jed Macosko^3^

1) Helsinki University of Technology, Laboratory of Bioprocess Engineering, P.O. Box 6100, 02015 TKK, Finland

2) Department of Biology and Earth Sciences, University of Wisconsin-Superior, Superior, WI 54880-4500, USA

3) Department of Physics, Wake Forest University, Winston-Salem, NC 27109, USA

**Information sources for yeast genes**

Yeast duplicated gene pairs data was obtained from:

<http://www.broad.mit.edu/seq/YeastDuplication/S9_Trees/Duplicated_Pairs.xls>

Basic information about yeast genes was found at *Saccharomyces* Genome Database (SGD): <http://db.yeastgenome.org/cgi-bin/seqTools>

Yeast Protein Localization Server:

<http://bioinfo.mbb.yale.edu/genome/localize/>

**S1. Supplemental data for structural and amino acid substitution analysis of the duplicated genes**

**Table S1A. Structural positions of indels in the duplicated yeast genes.** Indels in comparison to the corresponding *K. waltii* protein were analyzed by comparing to structural information obtained from both proteins or in some cases from one protein in the yeast gene pair. The indels outside the known or modeled structures are not included in the table. Loops include turns.

Gene pair *K. waltii* Insertions Deletions Structural position Structure of

gene studied studied  yeast protein

*UGP1* 8105 1 - loop-helix border model

*YHL012W* 4 3 4 loops, 1 short strand deleted, model

1 helix deleted, 1 unclear

*PST2* 23042 - - model

*RFS1*  4 - 3 loops, 1 bent region of helix model

*MCK1* 22001 - - model

*YGK3*  3 1 2 loops, 2 strands no model

(based on MCK1 model)

*ACC1 BC* 6157 - - 1w93

*HFA1 BC* - 1 loop model

*ACC1 CT* 6157 - - 1od2

*HFA1 CT* 2 1 2 loops, 1 end of strand model

*RNR2* 15007 - - 1smq

*RNR4*  - 2 1 loop, 1 helix in RNR2 that is 1sms

a loop in RNR4

*CET1* 24238 - - 1d8h

*CTL1*  4 5 4 loops, 2 strands, 1 loop-helix, no model

2 unstructured (probably loops)

(based on 1d8h)

*VPS21* 2978 - - 1ek0

*YPT53*  1 - loop model

*SEC14* 7837 - - 1aua

*SFH1*  1 - short helix in long loop model

*SLT2* 5576 - - model

*YKL161C* - - no model

*GCS1* 4569 - - model

*SPS18*  1 - loop model

*CDC19* 6945 - - 1a3w

*PYK2*  - - model

*ADH1* 23198 - 1 loop model

*ADH5*  - - model

*GRS1* 3922 no model

*GRS2* no model

*ERV14* 1862 1 sequence in ER side no model

*ERV15* 1 sequence in ER side no model

*FEN1* 13644 no model

*ELO1* 2 1 no model

___________________________________________________________

**Table S1B. Nonsynonymous (dN) and synonymous substitution rates**

**and dN/dS ratios with standard error.** The dN and dS values were

calculated by the MEGA package. dN and dS values are shown for the

divergence of yeast genes from *K. waltii* gene (underlined). The values

for biotin carboxylase (BC) and carboxyl transferase (CT) domains of

ACC1 and HFA1 are shown also in the table.

___________________________________________________________

dN dS dN/dS

8105

*UGP1* 0.080 +0.010 1.240 +0.129 0.065 +0.010

*YHLO12W* 0.717 +0.050 1.368 +0.142 0.524 +0.066

23042

*PST2* 0.223 +0.033 1.216 +0.195 0.183 +0.040

*RFS1* 0.452 +0.052 2.193 +0.899 0.206 +0.088

22001

*MCK1* 0.178 +0.019 1.547 +0.235 0.115 +0.021

*YGK3* 0.684 +0.053 1.483 +0.214 0.461 +0.076

6157

*ACC1* 0.150 +0.008 0.964 +0.045 0.156 +0.011

*HFA1* 0.829 +0.027 1.211 +0.060 0.685 +0.041

*BC_ACC1* 0.085 +0.011 0.931 +0.088 0.091 +0.015

*BC_HFA1* 0.194 +0.016 1.997 +0.473 0.097 +0.024

706 amino acids region of CT

*CT_ACC1* 0.153 +0.013 0.871 +0.067 0.176 +0.020

*CT_HFA1* 0.308 +0.020 1.343 +0.114 0.229 +0.025

497 amino acids region of CT

*CT_ACC1* 0.148 +0.015 0.852 +0.078 0.174 +0.024

*CT_HFA1* 0.243 +0.021 1.440 +0.176 0.168 +0.025

15007

*RNR2* 0.128 +0.018 1.080 +0.124 0.119 +0.022

*RNR4* 0.404 +0.035 0.812 +0.079 0.497 +0.065

24238

*CET1*  0.206 +0.024 1.265 +0.185 0.163 +0.030*

*CTL1*  1.404 +0.176 1.081 +0.125 1.299 +0,222*

2978

*VPS21*  0.197 +0.030 1.754 +0.544 0.112 +0.039

*YPT53*  0.403 +0.045 1.386 +0.270 0.291 +0.065

7837

*SEC14*  0.129 +0.021 1.577 +0.330 0.082 +0.022

*SFH1*  0.294 +0.033 1.159 +0.175 0.254 +0.048

5576

*SLT2*  0.165 +0.018 1.803 +0.368 0.092 +0.021

*YKL161C* 0.476 +0.035 1.366 +0.158 0.348 +0.048

4569

*GCS1*  0.344 +0.035 1.448 +0.203 0.238 +0.041

*SPS18*  0.922 +0.084 1.111 +0.131 0.830 +0.124

6945

*CDC19* 0.092 +0.011 0.357 +0.034 0.258 +0.039

*PYK2*  0.234 +0.019 1.394 +0.154 0.168 +0.023

23198

*ADH1*  0.109 +0.015 0.502 +0.053 0.217 +0.038

*ADH5*  0.202 +0.022 1.094 +0.124 0.185 +0.029

3922

*GRS1*  0.132 +0.012 1.036 +0.084 0.127 +0.016

*GRS2* 0.409 +0.026 1.243 +0.114 0.329 +0.037

1862

*ERV14* 0.131 +0.027 1.314 +0.292 0.100 +0.030

*ERV15* 0.335 +0.046 1.453 +0.407 0.231 +0.072

13644

*FEN1*  0.193 +0.023 1.465 +0.216 0.132 +0.025

*ELO1*  0.399 +0.036 1.158 +0.129 0.345 +0.049

___________________________________________________________

* The region of N-terminal 54 amino acids in CTL1 and corresponding regions in other

proteins were excluded due to unclear alignment.

___________________________________________________________

**Fig. S1. Correlation between dN/dS ratio and dN.** The *CDC19* and *ADH1* values (shown in pink) are not included in the trend line since they deviate from other genes by slower synonymous substitution rate (dS), which makes the dN/dS ratio higher (see Table S1B). The R^2^ value for this trendline is 0.949 and p < 0.0005. The graph (not shown) for slow evolving genes (*CDC19* and *ADH1* excluded) had p = 0.0001 and the graph for fast evolving genes had p < 0.00005.

**S2. Supplemental data for UDP-glucose pyrophosphorylase (UGPase) genes *UGP1* and *YHL012W***

*UGP1* (*YKL035W* by systematic name) is a UDP-glucose pyrophosphorylase (UGPase) that catalyses the formation of UDP-glucose from UTP and glucose-1-phosphate. The enzyme also catalyses the reverse reaction, i.e. the pyrophosphorolysis of UDP-glucose. Information about the putative active site of yeast UGPase was obtained from the modeling study of the barley enzyme [1]. No function is known for the duplicated homologue *YHL012W* (systematic name).

**S2.1. Modeling**

*1Z90* (crystal structure for the putative *Arabidopsis thaliana* UGPase) was the template SWISS-MODEL used for modeling *UGP1* (54% identity with the 1z90 sequence) and *YHL012W* (35% identity with the 1z90 sequence). *UGP1* amino acids 1-499 and *YHL012W* amino acids 90-486 were modeled.

**________________________________________________________________________**

**Table S2A. Key residues important for activity of UGPases**

Barley UGPase G91 C99 W191 D226 K260 W302 K326 K364

*1z90* G87 C95 W187 D222 K256 W298 K322 K360

Kw8105 G111 C119 W211 D246 K280 W322 K346 K388

*UGP1*  G111 C119 W211 D246 K280 W322 K346 K388

*YHL012W* G107 **K**115 W207 D242 **N**276 W312 **S**336 **R**378

**Proposed function**

G-1-P binding x x x x x

PPi binding x x x

Mg^2+^ binding x

Catalysis? x

Functional data from Geisler et al. [1] for key binding site / active site residues of modeled barley UDP-glucose pyrophosphorylase (UGPase). In 1z90 and the models of *UGP1* and *YHL012W*, the side chains of the residues C95, W187 and W298 (in 1z90 numbering) are oriented away from the deep central groove whereas the other side chains line the groove.

**Table S2B. Selected differences in the deep central groove**

**around the putative active site residues of UGPases.**

*1z90* H192 D254 E271 I272 I318 E333 K401

*Kw8105* H216 D278 E295 V296 I342 E361 K428

*UGP1*  H216 D278 E295 V296 I342 E361 K428

*YHL012W* T212 V274 Y291 Y292 H332 K351 A419

**S2.2. Cellular localization**

A weak nuclear localization signal was detected for *UGP1* by Yeast Protein Localization Server. Nuclear localization prediction was stronger for *YHL012W*. Huh et al. report cytoplasmic location for *UGP1*, but no location for *YHL012W* [6].

**S2.3. Comments on *UGP1* and *YHL012W***

The identity between *UGP1* and *YHL012W* is 41%, and the difference includes numerous radical amino acid substitutions (e.g., see Table S2B). The key active site residues are conserved in other yeasts in general. The differences in *YHL012W* in the putative functionally important sites (Table S2A) are likely to influence significantly the PPi/glucose-1-phosphate binding and the enzymatic activity (probably detrimentally). The mutation of K329 to Gln in the potato tuber UGPase, corresponding to K326 of barley UGPase increased strongly the Km for PPi and glucose-1-phosphate [2]. *YHL012W* has serine at this site (S336), which is likely to affect the PPi/glucose-1-phosphate binding.

Evaluation of the meaning of putative active site differences is limited because the substrate was not modeled into the active site.

**S2.4. Conclusions**

It is likely that major differences exist in the catalytic activity or efficiency between *YKL035W* and *YHL012W*. It appears that the residues in the putative active site groove are diverging quite freely in *YHL012W*. *YHL012W* has accumulated differences in sites that are typically conserved in this gene family. In fact, *YKL035W* is a protein with highest relative divergence from *K. waltii* among duplicated yeast genes [3, Supplemental information S9, Duplicated Pairs]. This suggests that *YHL012W* has retained only a limited amount, if any, of the original activity.

**S3. Supplemental data for *PST2* and *RFS1* that show similarity**

**to trp repressor binding protein wrba**

*PST2* (*YDR032C* by systematic name) is a flavodoxin-fold protein. Its ohnolog partner is *RFS1* (*YBR052C* by systematic name). At the sequence level, a different gene, *YCP4* (*YCR004C* by systematic name), is a closer homologue (67% identity) to *PST2* than *RFS1* is (47% identity), but only *PST2* and *RFS1*are derived from the same whole genome duplication [3]. The role of *YCP4* is unclear [4].

The *PST2* -deletion and *RFS1*-deletion studies indicated that *PST2* and *RFS1*affect overlapping, partially redundant functions. Deletion of *RFS1*had a very similar phenotype to *PST2* -deletion, and furthermore, the *PST2* - *RFS1*-deletion double mutant showed a greater degree of suppression of the function of rad55∆ (deletion) than either single mutant [4]. Deletion of *YCP4* had no effect on rad55∆ (deletion) sensitivity.

**S3.1. Modeling**

Modeling by Swissmodel was based on *Pseudomonas aeruginosa* wrba structures *1zwl*, *1zwk* and *2a5l* (33% sequence identity with *PST2*). Flavin mononucleotide is bound in*1zwl*. Partially modeled binding pockets of *PST2* and *RFS1*are missing some residues from the aminoterminal region corresponding to R13, H14, G15, A16 and T17 of *1zwl*. These residues are facing the phosphate group in FMN.

**Table S3. Residues lining the partially modeled flavin mononucleotide (FMN)-**

**binding pocket in the crystal structure (*1zwl*) and the corresponding sites in yeast**

**proteins.**

*1zwl* P78 T79 R80 F81 T115 A116 S117 G120 G121

Kw23042 P120 T121 R122 F123 T157 G158 S159 G160 G161

*PST2*  P77 T78 R79 F80 T114 G115 T116 G118 G119

*YCR004C* P78 T79 R80 F81 T115 S116 S117 G120 G121

*RFS1*  P86 T87 K88 F89 G124 A125 I126 G130 D131

**S3.2. Examination of mutations in Swiss-PdbViewer**

The differing residues in *RFS1*(see Table S3) were introduced into the *1zwl* structure in Swiss-PdbViewer, and the following four effects were observed:

- The aliphatic part of the side chain of K88 in *RFS1*interacts with FMN in a way similar to that of side chain R80 in *1zwl.*

- T115G destroys a hydrogen bonding to FMN O2.

- S117I destroys a hydrogen bonding to FMN O2.

- G121D: Asp side chain is possibly too far to form a hydrogen bond to FMN.

**S3.3. Cellular localization**

There are conflicting reports for the location of *PST2* and *RFS1*. In earlier studies, the green fluorescent protein (GFP)-fusion protein of *PST2* localized to the cytoplasm in a punctuate pattern [5-7]. *PST2* is predicted from its sequence to be located at ER.

However, in a new study by Valencia-Burton et al. [4], the *PST2* -myc protein was associated in a nonrandom fashion with chromatin. They also found that flavodoxin fold proteins have a role in DNA repair or other DNA-related functions. In addition, two-hybrid analysis showed that *PST2* has interaction with the nuclear proteins *Ku80* and *Xsr2* [8]. Thus, these results indicate that *PST2* could function in the nucleus [4].

*YBR052C* was localized as green fluorescent protein (GFP)-fusion protein to the cytoplasm in a punctuate pattern [4, 6] Since the deletion of *RFS1*causes a phenotype similar to that of a *PST2* deletion, and since *RFS1*, like *PST2*, was reported to be chromatin-associated [4], it could also be localized to the nucleus. However, there is no clear sequence-based prediction for the localization of *RFS1*.

**S3.4. Conclusions**

There are likely to be some differences in the binding or effect of FMN in *RFS1*when compared to *PST2*.

**S4. Supplemental data for *MCK1* and *YGK3***

*MCK1* (*YNL307C* by systematic name) and *YGK3* (*YOL128C* by systematic name) are glycogen synthase kinase-3 (GSK-3) homologues. *MCK1* is involved in control of chromosome segregation and regulation of entry into meiosis ([9-11]; for review see [12]). *MCK1* down-regulates pyruvate kinase [13] that involves inhibition of a cAMP-dependent protein kinase [14]. *MCK1* also has a role in regulating the G2 to M transition in the cell cycle. Yeast *MCK1* protein kinase like GSK-3 shows a dual role, it autophosphorylates at tyrosine and serine but phosphorylates exogenous substrates at serine and threonine [10]. In addition to *MCK1*, there are three other GSK-3 homologues in yeast (*YGK3, RIM11 and MRK1*), but none of those three can supplement the role of *MCK1* [12].

Deletion of *YGK3* does not have any distinct phenotype. Nonetheless, *YGK3* can enhance some of the phenotypes of *MCK1* deletion [12]. The role of *YGK3* is rather redundant than additive, and the role of *MCK1* is the most prominent among all four paralogs in yeast [12]. Sequence comparison indicates that all residues important for kinase activity are conserved in *MCK1*, *RIM11* and *MRK1*, but not in *YGK3* [12].

Glycogen synthase kinase-3 is a ubiquitous serine/threonine/tyrosine kinase that phosphorylates and inactivates glycogen synthase. Thus, glycogen synthase is its substrate. In vitro studies of a 39 residue peptide from the C terminus of FRAT1, termed FRATtide, have shown that this peptide binds GSK-3 and can prevent Axin binding. Consequently, FRATtide inhibits the phosphorylation of Axin and β-catenin, but it does not inhibit GSK-3 activity toward peptides derived from *eIF2B* or glycogen synthase [15]. However, FRATtide binding does not prevent binding to glycogen synthase.

**S4.1. Modeling**

Model of *MCK1* amino acid region 61-349 was generated by SwissModel with a number of templates having 40-50% identity with *MCK1*, including 1j1c, 1j1b and 1gng (which are structures of human Glycogen Synthase Kinase-3
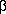
), and *1q5k*, *1o9u*, *1r0e*, etc. SWISS_MODEL was not able to create a model for *YGK3*. *YGK3* has 37% identity with *1r0e*, *1q5k*, *1j1b* and other glycogen synthase kinases.

­­­­­­­­_______________________________________________________________________

**Table S4A. Residues lining the ADP-binding pocket of GSK-3 homologues.**

­­­­­­­­_______________________________________________________________________

­­­­­­­­­­­­­­­

*MCK1* sites from the modeled structure (superposition with 1j1c):

*1j1c* A83 K85 V110 L132 Y134 V135 P136 T138 D181 N186

Kw22001 A57 K59 V84 M106 C108 I109 P110 T112 D155 N160

*MCK1*  A66 K68 V93 M115 C117 L118 P119 T121 D164 N169

*YGK3*  A72 K74 V99 M124 Y126 I127 P128 T130 D173 N178

*1j1c*  L188 C199 D200 S203

Kw22001 L162 C173 D174 S177

*MCK1* L171 C182 D183 S186

*YGK3*  L180 C191 D192 S195

*MCK1* sites from the alignment:

*1j1c* I62 G63 N64 G65 S66 F67 V70

Kw22001 I32 G33 H34 G35 A36 F37 V40

*MCK1* I41 G42 R43 G44 A45 F46 V49

*YGK3* I47 G48 H49 G50 S51 F52 V55

All Kw22001 and *YGK3* sites are from the sequence alignment.

**S4.2. Comments on ADP-binding pocket**

Y134 (*1jlc*) and C117 (*MCK1*) side chains are approximately equally far from the adenine rings. V135 (*1j1c*) and L118 (*MCK1*) have no close contact to ADP (side chain points away from ADP). N64 (1jic) side chain also points away from ADP, and thus, R43 (*MCK1*) and H49 (*YGK3*) are likely to do the same. S66 is 4.9Å away from the phosphate group of ADP in *1j1c*. Ala at this position is not likely to have any major effect (Kw22001 and *MCK1*, except *YGK3* has Ser at this site). There were no clashes with ADP in the partial *MCK1* model when superimposed with *1j1c*. Thus, *YGK3* does not seem to have any major differences in the ADP-binding pocket.

**S4.3. Phosphotyrosine and sulfate-binding site**

The mouse glycogen synthase kinase-3 beta (GSK-3β) structure (1gng) contains sulfate liganded to side chains R96, R180 and K205 and main chain nitrogen of V214. In GSK-3, Tyr-216 is phosphorylated in the active site. All sequences in the Table S4A contain the corresponding tyrosine. The sulfate-binding site near this tyrosine is conserved in the *K. waltii* gene and in *MCK1*, but in *YGK3* this binding site is apparently destroyed. In addition, 1gng has Val-214 between the sulfate and Tyr-216, but *YGK3* has Lys in this position, which, on the basis of 1gng structure, could block the correct functioning of the sulfate ion. The sulfate ion at this site is thought to bind phosphoserine in the substrates. In the sulfate-binding site of *GSK-3β*, a R96A mutation severely impaired its ability to phosphorylate primed (phosphorylated on serine) substrates. This mutant was also resistant to inhibition by phosphorylation on Ser9 [17].

**S4.4. FRATtide-binding surface**

1gng is the structure of phosphorylated *GSK-3β* complexed with a peptide, a “FRATtide”, which inhibits beta-catenin phosphorylation [15]. The FRATtide-binding surface of 1gng was compared to the corresponding regions in MCK1 model and the sequence of *YGK-3* (Table S4B). The *MCK1* model did not have the full region corresponding to the FRATtide-binding surface. It was not possible to model certain MCK1 residues (e.g. residues colored green in Table S4C) by using 1gng as template in SWISS-MODEL due to an insertion in *MCK1* of amino acids residues 276-280, which occurs in the C-terminal side of the key tyrosine at position 271. The same insertion occurs in Kw22001, whereas *YGK3* has a deletion of two amino acids relative to 1gng. Due to these insertions and deletions, the sequence identity is very low between the four sequences for these regions. However, it is quite probable that these indels affect the substrate binding specificity.

When the differences in *MCK1* and *YGK3* to 1gng protein were introduced to the 1gng structure in Swiss-PdbViewer, it was observed that both proteins appear to have differences in the interaction surface corresponding to FRATtide-binding surface of *GSK-3β*, but in *YGK3* (see Tables S4C and S4D). Half of the FRATtide-binding site residues in the modeled *MCK1* area were the same in *MCK1* and 1gng (Table S4B). Inspection of the *MCK1* model did not reveal any major obstacles for the binding of FRATtide-like peptide. Most of the *YGK3* sites were different from those of 1gng and *MCK1* (Table S4C), some of them appear to have quite drastic effects (Table S4D). This indicates that the *YGK3* surface that corresponds to the FRATtide-binding surface in 1gng has either lost its function or is specialized to recognize a significantly different substrate. A weakness in this analysis is that the binding interactions of the actual (if exists) substrate of *MCK1* are not known. As a consequence, it is not possibly to know exactly how the differences in *YGK3* affect the interactions.

________________________________________________________________________

**Table S4B. One of the substrate (FRATtide)-binding surfaces in 1gng.** The positions differing in GSK-3β (1gng) from those of *KW22001* and *MCK1* are shown in blue. The positions differing in *YGK3* are shown in red. In green are shown 1gng positions in the FRATtide-binding region not modeled in *MCK1*.

1gng Y216 I228 F229 G230 S261 G262 V263 L266 V267

*Kw22001* Y190 I202 V203 G204 E235 P236 L237 L240 R241

*MCK1*  Y199 I211 I212 G213 E244 P245 L246 L249 R250

*YGK3*  Y208 L220 L221 N222 S253 A254 N255 L258 E259

1gng I270 T275 P276 I281 Y288 E290 F291 K292 F293

*Kw22001* S244 P249 P250 L255 Y262

*MCK1*  A253 P258 P259 I264 Y271

*YGK3*  A262 R267 F268 I273 Q280

1gng P294 I296

________________________________________________________________________

________________________________________________________________________

**Table S4C**. **Possible effect of differences between GSK-3β and *MCK1* to the potential substrate-binding site in *MCK1*.** When the amino acid residues differing in *MCK1* from the GSK-3β (1gng) sequence (see Table S4B) were introduced in Swiss-Pdb Viewer to the corresponding positions of GSK-3, the following observations were made for these mutations.

________________________________________________________________________

F229I Probably retains the hydrophobic interaction with L212 of FRATtide.

G262P Forms hydrophobic interaction to P199 of FRATtide.

V263L Possibly stronger hydrophobic interaction with L203 of FRATtide is

formed, whereas the unfavorable interaction between hydrophobic amino acid (Val) and charged Arg is shifted to a differing position (longer side chain in Leu).

V267R An Arg side chain is introduced near to R219 of FRATtide. However,

there are three carboxylic acids at 3-8 Å distance near to position 267 in GSK-3β (1gng) that could neutralize the charges of both arginines.

1270A Increases a hydrophobic cavity between GSK-3 and FRATtide.

T275P Increases hydrophobicity in the same cavity, in which I270 is located.

________________________________________________________________________

**S4.5. Cellular localization**

Mitochondrial localization is predicted by Yeast Protein Localization Server for *MCK1*, whereas nuclear localization is predicted for *YGK3*. Huh et al. report both cytoplasmic and nuclear localization for MCK1 [6]. Nuclear role is supported by the findings that *MCK1* has a role in control of mitotic chromosome segregation and in regulating entry into meiosis and interacts with centromere binding proteins [16]. No experimental localization data was available for *YGK3*.

____________________________________________________________________________

**Table S4D**. **Possible effect of differences between GSK-3β and *YGK3* to the potential substrate-binding site in *YGK3*.** When the amino acid residues differing in *YGK3* from the *GSK-3β* (1gng) sequence (see Table S4C) were introduced in Swiss-PdbViewer to the corresponding positions of *GSK-3*, the following observations were made for these mutations.

______________________________________________________________________________

I228L Probably no major effect on the interactions between GSK-3β and FRATtide

F229L May weaken the hydrophobic interaction with L212 and I213 in FRATtide.

G230N May not have any significant effect on the interactions with FRATtide

G262A Strengthens hydrophobic interaction with L203 of FRATtide.

V263N A potential H-bond formed from Asn side chain to R219 NE1 of FRATtide

V267E Disturbs hydrophobic interactions between GSK-3β and FRATtide

I270A Reduces hydrophobic interaction between GSK-3β and FRATtide

T275R Arg side chain clashes with hydrophobic I213 and V217 side chains

P276F Rotamer proposed by Swiss-Pdb Viewer made clashes with residues of *GSK-3β*

and FRATtide; torsion of Phe at 276 allowed a position to be found in which

hydrophobic interaction between GSK-3β and FRATtide could occur.

Y288Q Y288 forms a potential hydrogen bond to main chain N1 of L212 in FRATtide, but it is possible that a hydrogen bond is formed from Gln to the main chain of FRATtide. The hydrophobic effect of the aromatic ring in Tyr may also influence the interaction with FRATtide. This is lost in Y288Q mutation. Thus, Gln in this position of *YGK3* might cause very small weakening of the interaction.

______________________________________________________________________________

**S4.6. Conclusions**

While there are no essential differences in the ADP-binding pockets studied in these proteins (Table S4A), the *K. waltii* protein 22001, the *MCK1* protein and the *1j1c/1gng* structures differ significantly from *YGK3* in their potential substrate-binding region (a FRATtide-binding site in *GSK-3β*). The substrate binding activity of *YGK3* seems to have been compromised due to mutations, which implies that it is less regulated by FRAT1 homologs than *MCK1*. This suggests the intriguing possibility that YGK3 is regulated by a completely different substrate.

ADP-binding pocket of *YGK3* is clearly under purifying selection relative to the region corresponding to the FRATtide-binding surface and to the sulfate-binding site. The existence of purifying selection, although limited, indicates that YGK3 is functional. In particular, the fact that its ADP-binding site is conserved suggests that its function is under cellular regulation.

**S5. Supplemental data for acetyl-CoA carboxylase genes *ACC1* and *HFA1***

*ACC1* (*YNR016C* by systematic name) is biotin containing enzyme that catalyzes the carboxylation of acetyl-CoA to form malonyl-CoA and is involved in the cytoplasmic fatty acid synthesis. The duplicate gene *HFA1* (*YMR207C* by other name) codes the corresponding mitochondrial enzyme [18]. HFA1 contains upstream from the first aminoterminal methionine a mitochondrial targeting signal and protease cleavage site. *ACC1* does not have this extension. *HFA1* appears to have a non-AUG translation signal, and thus, its expression level is low [18].

**S5.1. Modeling**

The crystal structure has been determined for the carboxyltransferase (CT) domain of yeast *ACC1* (*1od2*) [19] and for the biotin carboxylase (BC) domain (*1w93*) [20]. The CT domain of *HFA1* was modeled in SWISS-MODEL by using *1od2* and *1uyt* as the templates for the modeling. Acetyl-CoA is liganded to ACC1 in *1od2* structure. The superimposed structures were used to analyze the Acetyl-CoA-binding pocket in *HFA1*.

**Table S5. Residues lining the Acetyl-CoA-binding pocket in one subunit of the dimer**

Kw6157 K1589 I1590 S1592 S1622 I1626 R1728 V1730 G1731 I1732 Y1735

ACC1 (1od2) K1592 I1593 S1595 S1625 I1629 R1731 V1733 G1734 I1735 Y1738

HFA1 N1637 I1638 S1640 S1670 L1674 R1776 V1778 G1779 I1780 Y1783

Kw6157 I1752 L1753 T1754 G1755 P1757 A1758

ACC1 (1od2) I1755 L1756 T1757 G1758 P1760 A1761

HFA1 I1800 L1801 T1802 G1803 S1805 A1806

**S5.2. Comments on differences between *HFA1* and *ACC1*** **in the Acetyl-CoA-binding pocket**

The amino acid residues of *K. waltii* 6157 in the Acetyl-CoA-binding pocket are exactly the same as in *ACC1* (Table S5), and the same can be said for homologs in other yeasts, e.g. Q6CL34_KLULA (*Kluyveromyces* *lactis*) and Q5AAM4_CANAL (*Candida* *albicans*). As shown in Table S5, there are two amino acids that differ in *HFA1*, but, based on the modeled structure, these two differences may not have a significant impact on the interactions with Acetyl-CoA. For example, Leu-1674 in *HFA1* may form only a slightly weaker hydrophobic contact with the planar part of adenine moiety relative to Ile-1629 in *ACC1*. This weakened interaction stems from the fact that Leu-1674 cannot form the conformation adopted by Ile-1629, in which side chain carbons Cγ1 and Cδ1 are in distance of 3.4 and 3.9 Å, respectively, from the apolar planar edge of the acetyl-CoA adenine moiety. Still, this difference is minor and may not have any major effect to the catalytic activity, especially since the two catalytically important arginines (R-1954 and R-1731 in *ACC1*) are also present in *HFA1* [19]. Indeed, functional tests showed that *HFA1* expression in the cytoplasm restores the cellular enzyme activity when *ACC1* is defective [18].

**S5.3. Biotin carboxylase domain**

Amino acid sites binding ATP have been identified for the *E. coli* biotin carboxylase subunit and conserved BC domain sites have been reported [21]. These sites are highly conserved in BC domain of *HFA1* (Fig. S5A).

**S5.4. Mitochondrial targeting signal**

Huh et al. report punctate cytoplasmic localization pattern for *ACC1* and mitochondrial localization for *HFA1* [6]. While *ACC1* is predicted by yeast localization server to be cytoplasmic protein, *HFA1* contains a mitochondrial targeting signal in a region upstream from the first ATG codon. Functional tests showed that this region is needed for the mitochondrial function of *HFA1*. It seems that a non-ATG initiation signal is used to express HFA1 protein that is transported to mitochondria. *ACC1* protein is missing the mitochondrial targeting signal [18].

*Kluyveromyces lactis* gene for acetyl-CoA-carboxylase (Q6CL34_KLULA protein; gene databank accession number CR382126, locus tag KLLA0F06072g) contains upstream from the first ATG a sequence, which can be translated into protein with length of 85 amino acids (Table S5B). This sequence may contain a mitochondrial targeting signal, as was predicted by WoLFPSORT (<http://wolfpsort.seq.cbrc.jp/>) and TargetP (<http://www.cbs.dtu.dk/services/TargetP/>). The upstream sequence of *K. lactis* gene and *HFA1* contain also a predicted signal sequence. The identity between the *HFA1* and *K. lactis* upstream sequences is 21% when 6 gaps (1-3 amino acids in length) were allowed. Fig. S5B shows the alignment without gaps.

These results support the possibility that yeasts have, in general, only one acetyl-CoA-carboxylase gene that codes for both cytoplasmic and mitochondrial enzymes. In these cases, translation of the cytoplasmic protein would begin at the canonical initiation signal while the mitochondrial protein would start at a non-ATG initiation signal upstream from the first ATG and would be expressed at low levels.

It is likely that the genomic duplication in *S. cerevisiae* led to a situation in which one of the duplicate genes could loose the mitochondrial targeting signal, since the other gene copy retained the signal. The result was that there occurred specialization (subfunctionalization) of the duplicate gene copies. The presence of upstream mitochondrial localization signal in other yeasts remains to be studied.

**Fig. S5A. Important amino acid positions in biotin carboxylase domain.** The information about important sequence positions is from [21]. Conserved positions in the biotin carboxylase enzymes are shown in blue and the differing positions of HFA1 are shown in red. The sites corresponding to residues in the BC subunit of *E. coli* ACC interacting with ATP are indicated by dots.

Kw6157 GGHTVISK**VLIAN**N**G**IA**A**V**K**EI**R**SVRKWAYETFGNERAVQFVAMATPEDLEANAEYLRM**A**

ACC1 GGHTVISKILIANNGIAAVKEIRSVRKWAYETFGDDRTVQFVAMATPEDLEANAEYIRMA

HFA1 GGHTVISKILIANNGIAAVKEMRSIRKWAYETFNDEKIIQFVVMATPDDLHANSEYIRMA

********:************:**:********.::: :***.****:**.**:**:***

Kw6157 **D**QYVE**V**PGGTNNNN**Y**ANVDL**IV**ELAERADVD**AV**WA**G**W**G**HA**SE**NPLLPERLAASPRKVI**FI**

ACC1 DQYIEVPGGTNNNNYANVDLIVDIAERADVDAVWAGWGHASENPLLPEKLSQSKRKVIFI

HFA1 DQYVQVPGGTNNNNYANIDLI**L**DVAEQTDVDAVWAGWGHASENPCLPELLASSQRKILFI

***::************:***:::**::**************** *** *: * **::**

●

Kw6157 **GP**PGNA**M**RS**LG**D**K**ISSTIVAQHAKVPC**I**PWSGTGVDQVHLDEENGLVSVTDDIYQKGCCD

ACC1 GPPGNAMRSLGDKISSTIVAQSAKVPCIPWSGTGVDTVHVDEKTGLVSVDDDIYQKGCCT

HFA1 GPPGRAMRSLGDKISSTIVAQSAKIPCIPWSGSHIDTIHIDNKTNFVSVPDDVYVRGCCS

****.**************** **:*******: :* :*:*::..:*** **:* :***

●●● ●●●

Kw6157 SPEDGLAKAKKI**GFPVM**V**KA**SE**GGGGKGIR**KVEREQ**D**FIPLYKQAAN**E**IPGSPIFIM**K**LA

ACC1 SPEDGLQKAKRIGFPVMIKASEGGGGKGIRQVEREEDFIALYHQAANEIPGSPIFIMKLA

HFA1 SPEDALEKAKLIGFPVMIKASEGGGGKGIRRVDNEDDFIALYRQAVNETPGSPMFVMKVV

****.* *** ******:************:*:.*:***.**:**.** ****:*:**:.

● ● ● ● ●

Kw6157 GNA**RHLEVQL**LA**D**QY**GT**NISLFG**RDCSVQRRHQKIIE**E**AP**VTIAKPDTFTEMERSAVRLG

ACC1 GRARHLEVQLLADQYGTNISLFGRDCSVQRRHQKIIEEAPVTIAKAETFHEMEKAAVRLG

HFA1 TDARHLEVQLLADQYGTNITLFGRDCS**I**QRRHQKIIEEAPVTITKPETFQRMERAAIRLG

*****************:*******:***************:*.:** .**::*:***

● ● ● ●

Kw6157 KL**VGY**VS**AGT**V**EYL**YSHDDDKFY**FLE**L**N**P**RLQVE**HPT**TE**M**V**SGVN**L**PAAQ**L**Q**I**AMGIP**M**H

ACC1 KLVGYVSAGTVEYLYSHDDGKFYFLELNPRLQVEHPTTEMVSGVNLPAAQLQIAMGIPMH

HFA1 ELVGYVSAGTVEYLYSPKDDKFYFLELNPRLQVEHPTTEM**I**SGVNLPATQLQIAMGIPMH

:*************** .*.********************:*******:***********

Kw6157 RIKDIRLMYGVDPHTATEIDFDFQRRPTPKGHCTACRITSEDPNEGFKPSGGSLHELNFR

ACC1 RISDIRTLYGMNPHSASEIDFEFQRRPIPKGHCTACRITSEDPNDGFKPSGGTLHELNFR

HFA1 MISDIRKLYGLDPTGTSYIDFKNLKRPSPKGHCISCRITSEDPNEGFKPSTGKIHELNFR

*.*** :**::* :: ***. :** ***** :*********:***** *.:******

Kw6157 SSSNVWGYFSVSSSGGIHSFSDSQFGHIFAFGENRQASRKHMVVALKELSIRGDFRTTVE

ACC1 SSSNVWGYFSVGNNGNIHSFSDSQFGHIFAFGENRQASRKHMVVALKELSIRGDFRTTVE

HFA1 SSSNVWGYFSVGNNGAIHSFSDSQFGHIFAVGNDRQDAKQNMVLALKDFSIRGEFKTPIE

***********...* **************.*::** ::::**:***::****:*:*.:*

Kw6157 YLIKLLETEDFEGNSITTGWLDDLISQK

ACC1 YLIKLLETEDFEDNTITTGWLDDLITHK

HFA1 YLIELLETRDFESNNISTGWLDDLILKN

***:****.***.*.*:******** ::

**S5.5. Conclusions**

A major difference between ACC1 and HFA1 is localization. The significance of higher divergence in HFA1 is not fully clear, although it might be related to the mitochondrial environment.

_________________________________________________________________________

**Fig. S5B. Aminoterminal sequence and upstream translation of Q6CL34__Klula and**

***HFA1*.** The first methionines are shown in bold black and the putative signal peptide

cleavage site in *HFA1* [18] is shown in bold blue. Klula denotes for

*Kluyveromyces lactis*.

_________________________________________________________________________

Q6CL34_KLULA RLKKVLLKRVSINRIVRLLVSFFQKLSIIIIIVTLIKLTNLTLYRLFPVL

HFA1 ----------KGKTITHGQSWGARRIHSHFYITIFTITCIRIGQYKLALY

. : *.: ::: : *. : :.:

Q6CL34_KLULA ARHSRFIPLANKFTVHFSIFSPRLFHSTRNILRSK**M**SEENLSEVSISQSK

HFA1 LDPYRFYNITGSQIVRLKGQRPEYRKRIFAHSYRHSSRIGLNFPSRRRYS

** ::.. *::. *. : : *. .*. * : .

Q6CL34_KLULA QYEITEYSDRHSKLASHFIGLNTVDKADDSPLKEFVKSHGGHTVISKVLI

HFA1 NYVDRGNIHK**HTRL**PPQFIGLNTVESAQPSILRDFVDLRGGHTVISKILI

:* .:*::*..:*******:.*: * *::**. :********:**

Q6CL34_KLULA ANNGIAAVKEIRSVRKWAYETFGDERTVQFVAMATPEDLEANAEYIRMAD

HFA1 ANNGIAAVKE**M**RSIRKWAYETFNDEKIIQFVVMATPDDLHANSEYIRMAD

**********:**:********.**: :***.****:**.**:*******

**S6. Supplemental data for ribonucleotide reductase genes**

***RNR2* and *RNR4***

Class I ribonucleotide reductases (RNRs) catalyze the reduction of ribonucleotides to

deoxyribonucleotides. Eukaryotic RNRs are formed of two subunits: R1 subunit contains substrate and allosteric effector-binding sites, and the R2 subunit contains a catalytically essential diiron-tyrosyl radical cofactor. *RNR2* (*YJL026W* by systematic name) and *RNR4* (*YGR180C* by systematic name) are the small subunit genes. Crystal structures have been determined for yeast *RNR2* and *RNR4* in homodimeric and heterodimeric forms [22, 23]. The structure-function aspects of the differences between *RNR2* and *RNR4* in homodimers and heterodimers are reported by Sommerhalter et al. [22].

*RNR4* is about 50 amino acids shorter at the N-terminus than *RNR2*. The *RNR4* protein lacks 6 out of 16 residues conserved in most R2 proteins [23], including three residues involved in coordinating iron (Table S6A). The consequence is that *RNR4* cannot accommodate a diiron center. However, *RNR4* is required to activate *RNR2*, which includes stabilization of the diiron center. The only major difference between RNR2 in the homodimeric and in the heterodimeric form is that there is more disorder in the helix αB in the homodimer. The helix αB provides one of the ligands, Asp145, to the diiron center. The heterodimer is likely to be the functionally dominant form. There are indications that heterodimer is more stable than the homodimer [22]. The dimerization surface is largely conserved in *RNR4*, although some changes are found and some of them could in principle be involved in the higher stability of the heterodimer.

The reason for the higher disorder in the helix αB in *RNR2* homodimer lies not in the amino acid sequence of the helix αB itself, since this helix is highly conserved in *RNR2* and it is exactly the same in *K. waltii* (see Table S6). Rather, the higher disorder stems from weakened dimerization contact in the homodimer due to a mutation in the dimer interface [22].

In *RNR4*, the helix αB has mutated extensively (Table S6B), although we cannot say how much of the mutations are due to relaxed selection pressure and how much there are adaptive changes, if any. It is more likely that the αB helix of *RNR4* is highly mutated because its structure is no longer critical for the function of *RNR4*. For example, this region of *RNR4* has accumulated many amino acid residues that have a low helix propensity (Gly, Asn, Ser, Tyr, Thr).

_________________________________________________

**Table S6A. Conserved iron ligand-binding site in diiron**

**center of RNR proteins.** Sites for yeast *RNR2* and *RNR4*

are from [23].

_________________________________________________

Kw15007 D147 E178 H181 E241 E275 H278

*RNR2* D145 E176 H179 E239 E273 H276

*RNR4* D93 E124 Y127 E186 R220 Y223

_________________________________________________

**________________________________________________________________**

**Table S6B. Sequences of the helix αB region in RNR proteins.** Aspartate (Asp-145 in *RNR2*) that forms a ligand to diiron center is shown in blue.

Kw15007 ENERFFISRVLAFFAAS**D**GIVNENL

*RNR2* 128 ENERFFISRVLAFFAAS**D**GIVNENL 152

Q6CJY2_KLULA ENERFFISRILAFFAAS**D**GIVNENL

Q75F64_ASHGO ENERFFISRVLAFFAAS**D**GIVNENL

Q6FW29_CANGA ENERFFISRVLAFFAAS**D**GIVNENL

Q5A0L0_CANAL ENERYFISRVLAFFAAS**D**GIVGENL

*RNR4* 76 DDQKTYIGNLLALSISS**D**NLVNKYL 100

KLULA , Kluyveromyces lactis, strain NRRL Y-1140**;** ASHGO, Ashbya gossypii **;** CANGA,

Candida glabrata, strain CBS138**;** CANAL, Candida albicans, strain SC5314.

It thus appears that the yeast ribonucleotide reductase has evolved to function optimally with only one catalytically essential diiron-tyrosyl radical cofactor per *RNR2-RNR4* heterodimer [22]. In comparision, *K. waltii*, *S. kluyveri* and many other fungi have only one ribonucleotide reductase gene (*RNR2*) that presumably operates as a homodimer in each of these organisms.

We may try to understand how the evolution of yeast *RNR2* and *RNR4* genes occurred. When yeast had after the gene duplication two RNR genes (*RNR2* and *RNR4*), then there was at first a situation in which both homodimers and heterodimers functioned equally, because there was no difference in the proteins. Later, there could have appeared a mutation that first strengthened the interaction between the heterodimer, but it is not necessary. The easiest way to explain the evolution of *RNR2*/*RNR4* system is a degenerative model, in which redundant functions in *RNR2* and *RNR4* proteins became removed because of the lack of purifying selection. The purifying selection was lacking if a degenerative mutation happened in one gene and the other gene still provided the function. Still, it is not ruled out that also a new property was gained to improve the functionality of the heterodimer over homodimer. The lost functions are the ability of the *RNR2* homodimer to function efficiently and the maintenance of the catalytic diiron center in *RNR4*. The degenerative evolution is seen also in the disruption of the helix α5 by Pro-146 in *RNR4*. Table S6B shows how far the sequence divergence in *RNR4* has gone. The loss of essential properties in RNR4 has probably been accelerated due to higher evolution rate in *RNR4*. The probable reason for this is that *RNR4* does not need all its former structural and functional properties to be able to stabilize *RNR2*. It is open question if there has happened adaptive changes in addition to the degenerative changes; for example, whether the heterodimer was selected because it was more stable than the homodimer.

**S6.1. Cellular localization**

During the normal cell cycle, *RNR2* and *RNR4* are predominantly localized to the nucleus. Under genotoxic stress, *RNR2* and *RNR4* become redistributed to the cytoplasm in a checkpoint-dependent manner [24]. Huh et al. report both cytoplasmic and nuclear localization for RNR2 and RNR4 [6]. Cytoplasmic location is predicted by Yeast Protein Localization Server for *RNR2* and *RNR4*. There is also a weak nuclear prediction for both proteins.

**S6.2. Conclusions**

It is likely that a basically degenerative evolution has formed a novel specialized system, in which the functions of one gene are now divided into two genes. Thus the yeast ribonucleotide reducatase offers a good example of a quite recent functional divergence and subfunctionalization of duplicated genes. Neofunctionalization may also be behind the better functionality of the heterodimer.

**S7. Supplemental data for RNA triphosphatase genes *CET1* and *CTL1***

*CET1* (*YPL228W* by systematic name) protein is divalent cation dependent RNA triphosphatase, which catalyses the first step in mRNA cap formation. *CET1* cleaves the β-γ phosphoanhydride bond of 5’-triphosphate RNA to yield a diphosphate end that is then capped with GMP by RNA guanyltransferase (*CEG1*). *CET1* and *CEG1* form an enzyme complex.

In *CET1*, the first 230 amino acids form a domain that is not needed for catalysis. The catalytic domain is formed by the amino acids 275-549 [25]. *CTL1* (*YMR180C* by systematic name) contains only the catalytic domain region. *CTL1* has experienced a truncation of the aminoterminal part of the protein (~210 amino acids). *CTL1* is 21% identical to the corresponding part of *CET1* sequence. The alignment of *K. waltii* 24238 and *CET1* in the aminoterminal domain contains several gaps (Kw24238 is shorter), indicating that functional constraints in this region are not very strict (Fig. 7).

The biochemical and cellular role of *CTL1* has been studied by Rodriguez et al. [26]. *CTL1* is not essential for cell viability and has lost its ability to associate with the capping machinery. Catalytically essential glutamate and arginine residues are conserved in *CTL1* [26]. *CTL1* does not interact with *CEG1* protein. In the presence of magnesium, *CTL1* (like *CET1*) has triphosphatase activity. *CET1* and *CTL1* have also ATPase activity in the presence of manganese. In *CTL1* but not in *CET1*, manganese inhibits the triphosphatase activity. Since *CTL1* gene is transcribed, it is possible that it has a role, maybe in RNA degradation or in processing RNA other than mRNA [26].

**S7.1. Modeling**

Crystal structure has been determined for *CET1* (*1d8i*, *1d8h*; [25]). SWISS-MODEL could not model *CTL1*. Sequence alignment was used to analyze the key active site residues, which are reported by Bisaillon and Shuman [27].

**S7.2. Sequence features of *CTL1***

Out of 15 amino acid residues important to the catalytic activity [27] only one site (*CET1* site 469) shows a difference in *CTL1* (see Fig. S7). Arg469 in *CET1* interacts via water with γPO_4_. *CTL1* has histidine at this position, and in addition, an insertion of leucine before histidine (see Fig. S7).

Sites important for the homodimerization of *CET1* have been identified [28]. However, there is variation in these sites between *K. waltii* 24238, *CET1* and *CTL1* (Fig. S7), and thus, it is unclear how the variation reflects the properties of *CTL1*.

The reason for the loss of the ability of *CTL1* to bind *CEG1* is obvious. The *CEG1*-binding motif (WAQKW) identified in *CET1* [29] is completely missing in *CTL1* (Fig. S7). While *CET1* is 57% identical to *K. waltii* 24238 protein, the identity of *CTL1* with *K. waltii* protein is only 21%. *CTL1* apparently has a function that does not require all activities the gene originally had, and thus, many sequence properties have eroded away, including the aminoterminal domain and *CEG1*-binding site. The very high divergence rate also indicates highly relaxed functional constraints. Amazingly, the catalytically important residues are still practically untouched (Fig. S7), indicating the presence of a strong purifying selection in those residues. 14 out of 15 sites known to be catalytically important are kept as well as over 40 other sites. This indicates that *CTL1* has retained the original catalytic activity.

**Fig. S7. Sequence alignment of *K. waltii* 24238, *CET1* and *CTL1*.** The 15 catalytically important sites (from [27]) are shown in bold blue and the numbering of these sites according to *CET1* is shown above the sequence. Sites important for dimerization in *CET1* are shown in bold black. The *CEG1*-binding motif in *CET1* is shown in red. The alignment was created by Clustal X (1.83).

___________________________________________________________________________

Kw24238 MSNSK--PNVNRGLSLEDLVNHDDR------YNSKTSNKPNPLPS---AEVKKRLSFDDS

CET1 MSYTDNPPQTKRALSLDDLVNHDENEKVKLQKLSEAANGSRPFAENLESDINQTETGQAA 60

CTL1 ------------------------------------------------------------

Kw24238 ASDANTSMNSPQAPRYSKGSKKNSEGDEETDTDDDVGGSGDIVFETGDFKFDYDKQE---

CET1 PIDNYKESTGHGSHSQKPKSRKSSNDDEETDTDDEMGASGEINFDS-EMDFDYDKQHRNL 119

CTL1 ------------------------------------------------------------

Kw24238 ---------DGEKGKARSAK-----LEIDAQSEAKSKIKKETD-----------------

CET1 LSNGSPPMNDGSDANAKLEKPSDDSIHQNSKSDEEQRIPKQGNEGNIASNYITQVPLQKQ 179

CTL1 ------------------------------------------------------------

Kw24238 -------------------------VKDIFQERASSQSKRNAIKKDLNLLSEIAATAKPS

CET1 KQTEKKIAGNAVGSVVKKEEEANAAVDNIFEEKATLQSKKNNIKRDLEVLNEISASSKPS 239

CTL1 -------------------------------MSDQPETPSNSRNSHENVGAKKADANVAS

:: * : . :: : : : .*

Kw24238 RYHVAPIWAQKWKPTVKALQSIDTKDLNIDAS**FT**NIIPDD**D**LTKSVQDWVYATLVSIPPD

CET1 KYRNVPIWAQKWKPTIKALQSINVKDLKIDPS**FL**NIIPDD**D**LTKSVQDWVYATIYSIAPE 299

CTL1 KFRSLHIS--------ETTKPLTSTRALYKTT**RN**NSRGAT**E**FHKHVCKLAWKYLACIDKS

::: * :: :.: . ..: * :: * * . .: : .* .

305 307

Kw24238 QRQYI**E**M**E**MKYGLIVEGSDSNRVSPPVSSQ**TV**YTDMDAHLTPDVDERVFNEINRYVKGIS

CET1 LRSFI**E**L**E**MKFGVIIDAKGPDRVNPPVSSQ**CV**FTELDAHLTPNIDASLFKELSKYIRGIS 359

CTL1 SISHI**E**I**E**MKFGVITDKRTHRRMTP-HNKP**FI**VQNRNGRLVSNVPEQMFSSFQELLRSKS

..**:***:*:* : *:.* .. : : :.:*..:: :*..:.. ::. *

377 393 409

Kw24238 ELSEYTG--KFNIIESHTT**D**LLYRVG--VSTQRPRFL**R**MSRDVKTGRVG-QFIE**K**RHVSQ

CET1 EVTENTG--KFSIIESQTR**D**SVYRVG--LSTQRPRFL**R**MSTDIKTGRVG-QFIE**K**RHVAQ 414

CTL1 ENPSKCAPRVVKQVQKYTK**D**SIYNCNNASKVGKLTSW**R**CSEDLRNKELKLTYIK**K**VRVKD

* .. . .. ::. * * :*. . .. : * * *::. .: :*:* :* :

433 454 456 458 469 471

Kw24238 LLLYSPKDSYDVKISINL**E**LPVPDNDPPEKYKDNTPVNT**R**T**K**Q**R**ISYIHNDSCT-**R**M**D**I**T**

CET1 LLLYSPKDSYDVKISLNL**E**LPVPDNDPPEKYKSQSPISE**R**T**K**D**R**VSYIHNDSCT-**R**I**D**I**T** 473

CTL1 FLIRYPQSSLDAKISISL**E**VPEYETSAAFRN---GFILQ**R**T**K**S**R**STYTFNDKMPL**H**L**D**L**T**

:*: *:.* *.***:.**:* :.... : : ***.* :* .**. . :*:*

492 494 496

Kw24238 KVANHNQGVKQRHTESTH**E**I**E**L**E**VNTAALLSAFENITQNSKEYASILRTFLNNGTIIRRK

CET1 KVENHNQNSKSRQSETTH**E**V**E**L**E**INTPALLNAFDNITNDSKEYASLIRTFLNNGTIIRRK 533

CTL1 KVTTTRRNS---HQYTSH**E**V**E**V**E**MD-PIFKETIS--ANDREKFNEYMCSFLNASDLIRKA

** . .:. : ::**:*:*:: . : .::. ::: ::: . : :*** . :**:

Kw24238 LTSLSYEIFEGQKKV-

CET1 LSSLSYEIFEGSKKVM 549

CTL1 AERDNMLTT-------

**S7.3. Cellular localization**

While *CET1* is located in the nucleus [6, 30], *CTL1* is found both in nucleus and cytoplasm [26]. Nuclear location is well-predicted for *CET1* (Yeast Protein Localization Server), whereas there is no strong location signal in *CTL1*; only a weak prediction for mitochondrial and nuclear locations was observed.

**S7.4. Conclusions**

The divergence of *CTL1* from *CET1* at sequence and functional levels is striking in its extent. The sequence identity is close to proceeding beyond recognition. The high conservation in the active site is thus remarkable and clearly demonstrates that *CTL1* has a cellular function based on the catalytic activity of the protein family. New role in the cell is evident.

**S8. Supplemental data for GTP-binding protein genes *VPS21***

**and *YPT53***

*VPS21* (also *YPT51,* and *YOR089C* by systematic name) and *YPT53* (*YNL093W* by systematic name) belong to the *Ypt*/*Rab* family of membrane-associated GTPases and are required for transport during endocytosis and for correct sorting of vacuolar hydrolases [31] [32, 33]. The structure of these proteins is similar to *Ras*. *Ras* and *Rab* proteins alternate between an inactive GDP-bound and an active GTP-bound form. Crystal structure has been determined for *VPS21* in active GppNHp-bound conformation (*1ek0*) [33]. GppNHp is a slow-hydrolyzable GTP analogue.

The paralogous genes formed in the genomic duplication from the single gene are *VPS21* and *YPT53*; the identity between these two proteins is 64%. *YPT52* is another paralogous member in the gene family. While *VPS21* and *YPT53* are 78% and 57% identical with Kw2978, respectively, *YPT52* is 43% identical with Kw2978. The *K. waltii* gene corresponding to yeast *YPT52* is 2394.

Mutational analysis showed that *VPS21* is more essential and important than *YPT52* and *YPT53*, although *YPT52* and *YPT53* are also required for the transport in the endocytic pathway and for correct sorting of vacuolar hydrolases [32]. This study indicated that *YPT53* may have a specialized function. *YPT53* is expressed in lower amounts in cells than *VPS21* [32].

**S8.1. Modeling and sequence analysis**

1ek0 is the crystal structure for *VPS21*. *YPT53* was modeled by SWISS-MODEL for the amino acid region 10-180. The structural templates were 1ek0, 1tu4 and 1tu3.

The key residues in the catalytically important GTP-binding pocket [33] are conserved in Kw2978, *VPS21* and *YPT53* (Table S8). The conserved GTP-binding sequence motifs of *Ras*-like proteins are present also in *YPT53* [33, 34]. These motifs are GXXXXGK(S/T), DXXG, NKXD and (T/G)(C/S)A. The *Rab*-specific LAPMYYR motif is found in *VPS21* and *YPT53* [32].

There is more variation in the second nucleotide-binding loop, which is 52-NEH-54 in *VPS21*, 57-DGK-59 in *YPT53* and 52-GDH-54 in Kw2978. The second nucleotide-binding site is probably a nonspecific binding site [33]. Also, other variable loops may be important for effector binding.

In superimposition of *YPT53* model with *1ek0*, Ser21 of *YPT53* is in 2.75 Å distance from the phosphate oxygen O3G of GppNHp in *1ek0*. After the change of Ala16 to Ser (in *1ek0*) in Swiss-PdbViewer, Ser at position 16 forms a hydrogen bond to the oxygen O3G. Ser at this position is common in other GTP-binding proteins of the protein family except yeasts have Ala. Thus, the hydrogen bonding to GTP appears to differ here between *VPS21* and *YPT53*, but it is not likely to have a major effect in the ability to bind GTP.

The side chains of Asn35 in *VPS21* and Ser40, the corresponding site in *YPT53*, point away from GTP, and thus this difference is not likely to have significant functional consequences.

There are hydrogen bonds from Asp123 to guanine (N1 and N2) in *1ek0*. The corresponding Asp128 in *YPT53* was not modeled to the correct place probably due to insertion of three amino acids in the C-terminal region from Asp128. It is likely that Asp128 in *YPT53* forms a similar contact to GTP than Asp123 in *1ek0*.

In *Rab*, the loop region corresponding to the *VPS21* loop α3-β5 has been characterized as one of the major determinants for specific effector protein binding, which could be important for specific membrane association [33, 35]. The differences between *VPS21* and *YPT53* in the loop α3-β5 might affect the effector specificity [33]. This loop is 108-QASKDI-113 in *VPS21* and 116-KVGHDI-121 in YPT53. The site corresponding to the effector-binding site in *Rab* is shown for larger Ypt/Rab family in Fig. S8. YPT53 sequence in this site differs dramatically from other homologous proteins.

___________________________________________________________________

**Table S8. Residues in the GppNHp-binding pocket.**

Kw2978 A16 A17 G19 S21 E34 N35 K36 P38

*VPS21* (*1ek0*) A16 A17 G19 S21 E34 N35 K36 P38

*YPT53*  S21 A22 G24 S26 E39 S40 K41 P43

*YPT52*  S12 S13 G15 S17 E30 L31 R32 S34

Kw2978 T39 A64 G65 Q66 K121 D123 S153 K155

*VPS21* (*1ek0*) T39 A64 G65 Q66 K121 D123 S153 K155

*YPT53*  T44 A69 G70 Q71 K126 D128 S161 K163

*YPT52*  T35 A68 G69 Q70 K126 D128 S175 K177

**Fig. S8. Effector-binding site of *Ypt/Rab* family.** The site is shown in bold.

___________________________________________________________________________________________________

Kw2978 VYDVTKPQSFIKARHWVKELRE**QASKDI**VIALVGNKLDIVESGGE-----

YPT51_YEAST VYDVTKPQSFIKARHWVKELHE**QASKDI**IIALVGNKIDMLQEGGE-----

Q6CTC6_KLULA VYDVTKPQSFIKARHWVKELHE**QASKGI**VIALVGNKMDLLESEED-----

Q75CK3_ASHGO VYDITKPQSFIKARHWVKELHE**QASKGI**VIALVGNKLDLLENGEA-----

Q6FNW1_CANGA VYDVTKPQSFIKARHWVKELQE**QASKDI**IIALVGNKIDVLENGTE-----

Q59X89_CANAL VYDITKPASFIKARHWVKELHE**QANRDI**TIALVGNKLDLVEDDSAEDGET

Q6BYB0_DEBHA VYDITKPASFIKARHWVKELHE**QASKDI**TIALVGNKYDLAENDNENE-ES

Q6C9Z5_YARLI VYDITKPQSFIKARHWVSELKS**QASPGI**IIALVGNKRDLVDDDE------

Q7RWE8_NEUCR VYDLTKPTSLIKAKHWVAELQR**QASPGI**VIALVGNKLDLTSDSAGSAEAS

Q4IBA7_GIBZE VYDLTKPTSLIKAKHWVAELQR**QASPGI**VIALVGNKLDLTGDSSSVAGAD

Q5B3G5_EMENI VYDVTKPSSLTKAKHWVAELQR**QASPGI**VIALVGNKLDLTNDGGETPAET

Q4WXU6_ASPFU VYDVTKPSSLTKAKHWVAELQR**QASPGI**VIALVGNKLDLTSDDGEAAEQP

YPT53_YEAST VFDVTNEGSFYKAQNWVEELHE**KVGHDI**VIALVGNKMDLLNNDDENE---

Q5B6I8_EMENI VYDITQASSLDKAKSWVKELQR**QANENI**VIALAGNKLDLVTENPD-----

Q4WP50_ASPFU VYDITQASSLDKAKSWVKELQR**QANENI**VIALAGNKLDLVTEHPD-----

Q98932_CHICK VYDITNTDTFVRAKNWVKELQR**QASPNI**VIALAGNKADLAT---------

RAB5A_MOUSE VYDITNEESFARAKNWVKELQR**QASPNI**VIALSGNKADLAN---------

RAB5A_HUMAN VYDITNEESFARAKNWVKELQR**QASPNI**VIALSGNKADLAN---------

RAB5C_CANFA VYDITNTDTFARAKNWVKELQR**QASPNI**VIALAGNKADLAS---------

RAB5C_MOUSE VYDITNTDTFARAKNWVKELQR**QASPNI**VIALAGNKADLAS---------

RAB5C_HUMAN VYDITNTDTFARAKNWVKELQR**QASPNI**VIALAGNKADLAS---------

___________________________________________________________________________________________________

**S8.2. Cellular localization**

Huh et al. report both cytoplasmic and nuclear localization for *VPS21*, and no localization for *YPT53* [6]. Yeast Protein Localization Server predicts *VPS21* and *YPT53* to be ER related. This is in line with the cellular role of *VPS21* and *YPT53* that are membrane-associated GTPases functioning in transport during endocytosis and sorting of vacuolar hydrolases [32, 33].

**S8.3. Conclusions**

The amino acids Ser21 and Ser40 in the GTP-binding pocket of *YPT53* (Table S8) differ from the *VSP21* and Kw2978 proteins, but these amino acids are found also in some other members of the large *Rab*-type GTP-binding protein family (not shown). Thus, GTP binding is likely to function quite normally in *YPT53*. A loop determining the effector specificity in the protein family has a differing sequence in *YPT53,* which indicates some divergence in the overall function.

**S9. Supplemental data for *SEC14* and *SFH1***

*SEC14* (*YMR079W* by systematic name) is phosphatidylinositol/phosphatidylcholine transfer protein involved in lipid metabolism. *SEC14* protein has cytoplasmic and *SFH1* protein (*YKL091C* by systematic name; should not be mixed with [*SFH1/YLR321C*](http://db.yeastgenome.org/cgi-bin/seqTools?seqname=S000004313&flankr=&flankl=&rev1=)) nuclear localization [36]. There are altogether five *SEC14* homologues (*SFH1*-*SFH5*) in yeast. *SFH1* has the highest sequence identity with *SEC14*, whereas functionally it is the most dissimilar to *SEC14* in this group [36, 37]. While *SFH2* and *SFH4* can complement the *SEC14* growth defect, *SFH1* can do it only partly. The functional tests showed that unlike *SEC14* (and *SFH2* and *SFH4*), *SFH1* was not able to control phosphatidylcholine degradation [36]. Accordingly, *SFH1* is neither a phosphatidylinositol nor a phosphatidylcholine transfer protein *in vitro* [38]. When overexpressed it complements the *SEC14*-related functions only to a very limited degree, and another reason for the weak growth complementation of *SEC14* deficiency could be that *SFH1* is localized to the nucleus and *SEC14* is predominantly a cytosolic protein [39]. Otherwise *SFH1* conserves all recognized critical structural motifs of *SEC14* [40].

**S9.1. Modeling and sequence analysis**

Crystal structure is available for *SEC14* (*1aua*). *SFH1* is 64% identical to *SEC14*. *1aua* has two β-octylglucoside molecules in the putative phospholipid-binding pocket, since crystallization required this detergent [40]. The crystal structure represents a transitional apo-conformation (for review see [41]). SWISS-MODEL modeled the amino acid region 1-301 of *SFH1* by using *1aua*, *1olm* and *1o6u* as the structural templates.

*SEC14* residues Lys66, Glu207 and Lys239 were concluded to be critical for the β-octylglucoside binding hydrogen bonding network [40, 42]. Lys66 and Lys239 are involved in phosphatidylinositol transfer activity [42]. The sites corresponding to *SEC14* residues Lys66, Glu207 and Lys239 are the same in *SFH1*. The β-octylglucoside-binding pocket is largely conserved in the *SFH1* protein, and especially the extremely hydrophobic putative phospholipid-binding surface is very conserved (Table S9).

**________________________________________________________________________**

**Table S9. The extremely hydrophobic putative phospholipid-binding surface.** The Sec14 sites are from [40].

Kw7837 L103 L105 V116 L117 Y119 V120 F138 L140 F147 F151

*SEC14* M177 L179 V190 M191 Y193 V194 F212 I214 F221 F225

*SFH1* L179 L181 V192 L193 Y195 I196 F214 I216 F223 F227

Kw7837 F154 L158 I166 I168

*SEC14* F228 L232 I240 I242

*SFH1* V230 L234 I242 I244

**S9.2. Cellular localization**

*SFH1* is localized to nucleus and *SEC14* is predominantly a cytosolic protein [39]. Nuclear localization is predicted for *SFH1*, whereas *SEC14* is predicted to be cytoplasmic by Yeast Protein Localization Server. Huh et al. report both cytoplasmic and nuclear localization for *SEC14* and *SFH1* [6].

**S9.3. Conclusions**

Although the basic functionally important sites are conserved in the fast evolving *SFH1* gene, *SFH1* has been adapted to perform a specialized function in nucleus, whereas *SEC14* functions in the cytoplasm. After gene duplication, *SFH1* has evolved a nuclear localization signal not present in *SEC14*. *SFH1* has experienced some functional reduction observed in functional tests. However, since SFH1 has a conserved phospholipid-binding pocket, but the phospholipid transfer activity is lost, it is possible that SFH1 has a new role that involves binding of phospholipids.

**S10. Supplemental data for *SLT2* and *YKL161C***

In budding yeast, a linear MAP (Mitogen Activated Protein) kinase phosphorylation cascade ends up with the activation of the SLT2-MAP kinase. In the phosphorylated form, *SLT2* kinase activates by phosphorylation at least two known downstream targets involved in the expression of cell wall-related genes and activation of cell cycle-regulated genes at the G1 to S transition [43, 44]. Phosphorylation lip, a regulatory loop near the active site, has a key role in the activation of the kinase activity. MAP kinases are activated by dual phosphorylation on a conserved threonine and a conserved tyrosine residue in the phosphorylation lip [45-47].

**S10.1. Sequence analysis**

*SLT2* (*YHR030C* by systematic name) has 50% identity and *YKL161C* has 43-44% identity with MAP kinases, in which the crystal structures have been determined (1tvo, 1erk, 1lez, 1lew, etc.). The C-terminal region (~110 amino acids in SLT2) contains much variation in *SLT2*, *YKL161C* and the *K. waltii* 5576 protein, and it contains also a region of poly-glutamines in *SLT2* and the *K. waltii* 5576 protein, whereas the poly-glutamines are missing from *YKL161C*. *YKL161C* is at the C-terminus almost 50 amino acids shorter than *SLT2* and over 50 amino acids shorter than *K. waltii* 5576. The region (over 350 amino acids) before the C-terminal variable region contains no indels between *S. cerevisiae* and *K. waltii.* The *SLT2* model was created by SWISS-MODEL for the amino acid region 6-361. No model was obtained for *YKL161C*. The key amino acids in several functional sites were analyzed at sequence level.

Although the ATP-binding region of *YKL161C* contains some differences, it is mostly conserved (Fig. S10).

The major difference in *YKL161C* is in the sites shown to be important for kinase activity (Fig. S10). The phosphate anchor motif (GXGXXG) is missing one conserved glycine (GXGXXS in YKL161C).

The essential TXY motif in the phosphorylation lip [45, 46], in which both Thr and Tyr are phosphorylated, is KXY in YKL161C. In the whole lip region (when determined on the basis of *MAPK14* lip region) 14 out of 23 positions in *YKL161C* have a different amino acid than *K. waltii* 5576 and *SLT2*.

**S10.2. Cellular localization**

*SLT2* and *YKL161C* are predicted to be nuclear proteins by Yeast Protein Localization Server. Huh et al. report both cytoplasmic and nuclear localization for *SLT2*, but no localization for *YKL161C* [6].

**S10.3. Conclusions**

The sequence comparison indicates that *YKL161C* is not likely to function as a MAP kinase, but it may bind ATP and docking protein(s). It may still function as a kinase.

**Fig. S10.** **Sequence alignment and functional sites of selected MAP kinases.** Lys-53 and Asp-168 (in light pink) are essential for kinase activity of *MAPK14* [48]. Phosphorylation lip is shown in blue. Phosphate anchor motif GXGXXG is shown. The ATP-binding region (adapted from [45, 49]) is shown by blue A letters above the sequence alignment. Core positions in the CD region are shown by double blue lines [50]. Key residues in the docking site of *MAPK14* identified by peptide binders are shown in light green [51]. Asp-316, essential for the binding of *MAPK14* to MAP kinase phosphatase-1 [52], is shown by black dot.

**A**

Kw5576 -------------------------------MVENLERHTFRVFNQEFTVDKRFQLIKEI 29

SLT2 -------------------------------MADKIERHTFKVFNQDFSVDKRFQLIKEI

YKL161C -------------------------------MATDTERCIFRAFGQDFILNKHFHLTGKI

MAPK14_Q16539 ------------------------------MSQERPTFYRQELNKTIWEVPERYQNLSPV 30

MAPK7_Q13164 MAEPLKEEDGEDGSAEPPAREGRTRPHRCLCSAKNLALLKARSFDVTFDVGDEYEIIETI

ERK2_P28482 -----------------------------MAAAAAAGAGPEMVRGQVFDVGPRYTNLSYI 31

**A**

**GXGXXG A A A A A A**

Kw5576 GHGAYGIVCSARFIEAAEETNVAIKKVTNVFSKTLLCKRSLRELKLLRHFRGHKNITCLY 89

SLT2 GHGAYGIVCSARFAEAAEDTTVAIKKVTNVFSKTLLCKRSLRELKLLRHFRGHKNITCLY

YKL161C GRGSHSLICSSTYTESNEETHVAIRKIPNAFGNKLSCKRTLRELKLLRHLRGHPNIVWLF

MAPK14_Q16539 GSGAYGSVCAAFDTKTG--LRVAV**K**KLSRPFQSIIHAK**R**TY**R**ELRLLKHMK-HENVIGLL 87

MAPK7_Q13164 GNGAYGVVSSARRRLTG--QQVAIKKIPNAFDVVTNAKRTLRELKILKHFK-HDNIIAIK

ERK2_P28482 GEGAYGMVCSAYDNVNK--VRVAIKKIS-PFEHQTYCQRTLREIKILLRFR-HENIIGIN 87

**A** **A AA**

Kw5576 DMDIVFSPNNTFNGLYLYEELMECDIHQIIKSGQPLTDAHYQSFIYQLLCALKYIHSADV 149

SLT2 DMDIVFYPDGSINGLYLYEELMECDMHQIIKSGQPLTDAHYQSFTYQILCGLKYIHSADV

YKL161C DTDIVFYPNGALNGVYLYEELMECDLSQIIRSEQRLEDAHFQSFIYQILCALKYIHSANV

MAPK14_Q16539 DVFTPARSLEEFNDVYLVTHLMG**A**DLN**NI**VK-**CQ**K**L**TDD**H**VQFLIYQILRGLKYIHSADI 146

MAPK7_Q13164 DILRPTVPYGEFKSVYVVLDLMESDLHQIIHSSQPLTLEHVRYFLYQLLRGLKYMHSAQV

ERK2_P28482 DIIR-APTIEQMKDVYIV**QD**LMETDLYKLLK-TQHLSNDHICYFLYQILRGLKYIHSANV 145

**111,115,116,119,120,122,126**

**A**

Kw5576 LHRDLKPGNLLVNADCQLKVCDFGLARGYSENPVENNQFL**TEY**VATRWYRAPEIMLSYQG 209

SLT2 LHRDLKPGNLLVNADCQLKICDFGLARGYSENPVENSQFL**TEY**VATRWYRAPEIMLSYQG

YKL161C LHCDLKPKNLLVNSDCQLKICNFGLSCSYSENHKVNDGFI**KGY**ITSIWYKAPEILLNYQE

MAPK14_Q16539 IHRDLKPSNLA**V**N**E**D**C**ELKIL**D**FGLAR-------HTDDEM**TGY**VATRWYRAPEIMLNWMH 199

MAPK7_Q13164 IHRDLKPSNLLVNENCELKIGDFGMARGLCTSPAEHQYFM**TEY**VATRWYRAPELMLSLHE

ERK2_P28482 LHRDLKPSNL**L**LNTTCDLKI**C**DFGLAR-VADPDHDHTGFL**TEY**VATRWYRAPEIMLNSKG 204

**158,160,162 170-185**

Kw5576 YTKAIDIWSCGCILAELLGGKPIFKGKDYVDQLNRILQVLGTPPEETLERIGSKNVQDYI 269

SLT2 YTKAIDVWSAGCILAEFLGGKPIFKGKDYVNQLNQILQVLGTPPDETLRRIGSKNVQDYI

YKL161C CTKAVDIWSTGCILAELLGRKPMFEGKDYVDHLNHILQILGTPPEETLQEIASQKVYNYI

MAPK14_Q16539 YNQTVDIWSVGCIMAELLTGRTLFPGTDHIDQLKLILRLVGTPGAELLKKISSESARNYI 259

MAPK7_Q13164 YTQAIDLWSVGCIFGEMLARRQLFPGKNYVHQLQLIMMVLGTPSPAVIQAVGAERVRAYI

ERK2_P28482 YTKSIDIWSVGCILAEMLSNRPIFPGKHYLDQLNHILGILGSPSQEDLNCIINLKARNYL 264

●

=========

Kw5576 HQLGYIPKVPFVTLYPQANVQALDLLEKMLTFDPQKRITVEEALEHPYLSIWHDPTDEPV 329

SLT2 HQLGFIPKVPFVNLYPNANSQALDLLEQMLAFDPQKRITVDEALEHPYLSIWHDPADEPV

YKL161C FQFGNIPGRSFESILPGANPEALELLKKMLEFDPKKRITVEDALEHPYLSMWHDIDEEFS

MAPK14_Q16539 QSLTQMPKMNFANVFIGANPLAVDLLEKMLVLDSDKRITAAQALAHAYFAQYHDPDDEPV 319

MAPK7_Q13164 QSLPPRQPVPWETVYPGADRQALSLLGRMLRFEPSARISAAAALRHPFLAKYHDPDDEPD

ERK2_P28482 LSLPHKNKVPWNRLFPNADSKALDLLDKMLTFNPHKRIEVEQALAHPYLEQYYDPSDEPI 324

Kw5576 CTEKFDFGFESVNEMEDLKQMILDEVRDFRQCVRQPLIEEEQAKQQQQQEQQLQQQQQQQ 389

SLT2 CSEKFEFSFESVNDMEDLKQMVIQEVQDFRLFVRQPLLEEQRQLQLQQQQQQQQQQQQQQ

YKL161C CQKTFRFEFEHIESMAELGNEVIKEVFDFRKVVRKHPISGDSPSSSLSLEDAIPQEVVQV

MAPK14_Q16539 -ADPYDQSFESRDLLIDEWKSLTYDEVISFVPPPLDQEEMES------------------

MAPK7_Q13164 CAPPFDFAFDREALTRERIKEAIVAEIEDFHARREGIRQQIRFQPSLQPVASEPGCPDVE

ERK2_P28482 AEAPFKFDMELDDLPKEKLKELIFEETARFQPGYRS------------------------

Kw5576 QHVHQLQQEHQNQAFMAEQHVIPDSYEDGDFKQHALFSQPSAGSNDIHDQFIGIHSDNLP 449

SLT2 -----------------QQPSDVDNGNAAASEENYPKQMATSNSVAPQQESFGIHSQNLP

YKL161C HP-------------------SRKVLPSYSPEFSYVSQLPSLTTTQPYQNLMGISSNSFQ

MAPK14_Q16539 ------------------------------------------------------------

MAPK7_Q13164 MPSPWAPSGDCAMESPPPAPPPCPGPAPDTIDLTLQPPPPVSEPAPPKKDGAISDNTKAA

ERK2_P28482 ------------------------------------------------------------

Kw5576 DHDTDFPPRPQENLLMSPMGLDNEGGGNSVEPAGSLDDFLDLEKELEFGLDRKSA-----

SLT2 RHDADFPPRPQESMMEMRPATGN---TADIPPQNDNGTLLDLEKELEFGLDRKYF-----

YKL161C GVN---------------------------------------------------------

MAPK14_Q16539 ------------------------------------------------------------

MAPK7_Q13164 LKAALLKSLRSRLRDGPSAPLEAPEPRKPVTAQERQREREEKRRRRQERAKEREKRRQER...

ERK2_P28482 ------------------------------------------------------------

**S11. Supplemental data for GCS1 and SPS18**

ADP ribosylation factors (ARFs) are members of the Ras superfamily of GTP-binding proteins. ARFs have very low intrinsic GTPase activity; the hydrolysis of GTP to GDP is dependent on ARF-GAPs. *GCS1* (*YDL226C* by systematic name) is a yeast ARF-GAP protein that functions in the ER-Golgi vesicular transport system [53, 54]. *GCS1* mediates the resumption of cell proliferation from the starved, stationary-phase state [55]. *SPS18* (*YNL204C* by systematic name) is expressed during sporulation [56]. *SPS18* is only 32 % identical to *GCS1*. It is about 30 amino acids shorter at the C-terminus than GCS1 and over 40 amino acids shorter than *K. waltii* 4569.

**S11.1. Modeling**

*GCS1* has 33% identity with 1dcq, the crystal structure of the mouse ARF-GAP domain and ankyrin repeats of *PYK-2* associated protein β [57], and 31% identity with 2b0o, the crystal structure of *UPLC1* GAP domain. *SPS18* has 25% identity with 1dcq.

SwissModel created a model for the *GCS1* aminoterminal region 1-126 by using 2crw, 1dcq and 2b0o as the templates. The model for SPS18 was for the region 13-98 by using 2crw and 1dcq as the templates. Thus, the models were obtained for the zinc finger region and almost the whole putative ARF-binding region in GCS1 (missing three residues). The model of *SPS18* is missing seven of the C-terminal positions corresponding to the ARF-binding positions in *Rattus norvegicus* *ARFGAP1*.

**S11.2. Binding site**

The N-terminal region of ARF-GAPs contains a zinc finger motif, in which four cysteines coordinate a zinc molecule, and this motif is required for the catalytic activity. The cysteines in the zinc finger region are fully conserved in *SPS18* (Fig. S11), and in the model that was created by SWISS-MODEL automatic server, the four cysteines were located in correct positions. Mandiyan detected by site-directed mutagenesis that the residues Trp274, Ile285, Arg292, Leu306 and Asp307 on the protein surface are required for full catalytic activity [57]. The corresponding residues are identical or similar both in *GCS1* and *SPS18* when compared to 1dcq, *K. waltii* 4569 and other yeast and fungus genes (not shown). Because the differences in *SPS18* are formed of conservative changes, they are not likely to have a major effect on the catalytic activity.

The crystal structure of the rat *ARF1* bound to ARF-GAP showed that the binding surface of ARF-GAP to *ARF1* is in the N-terminal region [58]. The residues involved in the binding are shown in Fig. S11. These sites are quite conserved in *GCS1* and Kw4569, whereas *SPS18* differs considerably. Especially, *SPS18* has an opposite charge in three positions when compared to *GCS1* and Kw4569. This is significant since salt bridges are important in the binding between ARF1 and ARFGAP [58]. In GCS1 model, all other residues in the putative ARF-binding region were exposed on the same side of the protein forming a surface, except the side chain of Lys72 was partly buried and the side chain of Arg125 (second last residue in the model) was pointing away from this surface while being exposed. There are no clear motif in *GCS1* and *SPS18* corresponding to the last three residues in the *ARF1*-binding region of ARF-GAP (134-136).

**Fig. S11. Zinc finger region and *ARF1*-binding site of *Rattus norvegicu*s ARFGAP1.** Alignment of *ARFGAP1* with yeast proteins is shown for the aminoterminal regions. The four cysteines and conserved arginine in the zinc finger region are shown by a black dot below the alignment. The binding positions of *ARFGAP1* are from crystal structure [58]. The binding sites in *ARFGAP1* are shown in blue and their numbering is shown above the sequences (Fig. 3 in ref 18). The differences are shown in differing colors; in red when *SPS18* differs from all others.

54

ARFGAP1 ------MASPRTRKVLKEVRAQDENNVCFECGAFNPQWVSVTYGIWICLECSGRHRGLG**V** 54

Kw4569 -MSEEWKVNPDNRRRLLQLQKVGSNKKCVDCEAPNPQWASPKFGIFICLECAGLHRGLG**V** 59

GCS1 --MSDWKVDPDTRRRLLQLQKIGANKKCMDCGAPNPQWATPKFGAFICLECAGIHRGLG**V** 58

SPS18 MRLFENSKDMENRKRLLRAKKAAGNNNCFECKSVNPQFVSCSFGIFICVNCANLLRGMG**T** 60

**●** **●** **●** **●** **●**

55 58 60 66 68 7071 112

ARFGAP1 **H**LS**F**V**R**SVTMD**K**W**K**D**IE**LEKMKAGGNAKFREFLEAQDDYEPSWSLQDKYSSRAAALF**R**DK 114

Kw4569 **H**IS**F**V**R**SITMD**Q**F**K**P**EE**LERMEKGGNEPFTEYLTSHGIDLK-LPLKVKYDNPIASDY**K**DK 118

GCS1 **H**IS**F**V**R**SITMD**Q**F**K**P**EE**LLRMEKGGNEPLTEWFKSHNIDLS-LPQKVKYDNPVAEDY**K**EK 117

SPS18 **N**IF**C**V**K**SITMD**N**F**E**E**KD**VRRVEKSGNNRFGSFLSKNGILQNGIPLREKYDNLFAKSY**K**RR 120

116 120 122 134 136

ARFGAP1 V**A**TLA**E**G**K**EWSLESSPAQN**WTP**PQPKTLQFTAH 147

Kw4569 L**T**ASI**E**G**T**TWEEPDRSSFD**PAS**LTSSGHAAAAA 151

GCS1 L**T**CLC**E**D**R**VFEEREHLDFD**ASK**LSATSQTAASA 150

SPS18 L**A**NEV**R**S**N**DINRNMYLGFN**NFQ**QYTNGATSQIR 153

**S11.3. Cellular localization**

Huh et al. report cytoplasmic localization for *GCS1*, but no localization for *SPS18* [6]. *GCS1* is predicted to be cytoplasmic and *SPS18* to be nuclear protein.

**S11.4. Conclusions**

While *SPS18* has probably retained its basic catalytic activity, it is likely that *SPS18* has lost its ability to interact with the same ARF protein than *GCS1*. *SPS18* is likely to have a specialized function. It is not likely that *SPS18* is becoming a pseudogene, because the zinc finger motif is intact.

**S12. Supplemental data for *CDC19* and *PYK2***

Pyruvate kinase is the last enzyme in the glycolytic pathway of sugar catabolism. It catalyzes the irreversible conversion of phosphoenolpyruvate into pyruvate. *CDC19* (also *PYK1*, and *YAL038W* by systematic name) is pyruvate kinase [59, 60], which functions as a homotetramer in glycolysis. Nearly all eukaryotic pyruvate kinases are tightly regulated and are activated by fructose-1,6-bisphosphate (FBP). Transcription of *CDC19* is induced in the presence of glucose.

*PYK2* (*YOR347C* by systematic name) is pyruvate kinase that appears to have essential differences in its role in yeast when compared to *CDC19*. *PYK2* transcription is repressed by glucose. *PYK2* protein is active without fructose 1,6-bisphosphate [60, 61]. *PYK2* enzyme activity is very low in yeast [61]. *PYK2* is apparently used by the cells only under very specific conditions [61], and it may be active under low glycolytic flux. *PYK2* has been found to be expressed in anaerobic growth on xylose [62].

**S12.1. Modeling**

Crystal structures of *CDC19* (1a3w, 1a3x) have been determined in complex with the allosteric regulator fructose-1,6-biphosphate and the substrate analog phosphoglycolate

[63]. *PYK2* has 71% identity with CDC19. Amino acids region 9-502 of *PYK2* was modeled by SWISS-MODEL based on the template structures 1a3w, 1a3x and 1liuD.

**Table S12. Residues lining the fructose-1,6-biphosphate-binding pocket in pyruvate kinases.**

Kw6945 L402 S403 T404 T405 S407 T408 R426 W453 D456

CDC19 (1a3w) L401 S402 T403 S404 T406 T407 R425 W452 D455

PYK2 L403 S404 T405 T406 N408 T409 R427 W454 D457

Kw6945 V457 R460 Q484 G485 H492 S493

CDC19 V456 R459 Q483 G484 H491 S492

PYK2 V458 R461 Q485 G486 H493 S494

**S12.2. Comments on the FBP-binding site**

In the crystal structure 1a3w, there is no hydrogen bond from T406 to FBP (after torsion of T406, a hydrogen bond can be formed). T406, which is a lysine residue in the *E. coli* enzyme, has previously been implicated in FBP binding by chemical modification (see [63] and references therein).When T406N mutation is introduced (in Swiss-PdbViewer) into 1a3w, a hydrogen bond is formed to FBP. T406S forms hydrogen bond to FBP, and also S406T (back mutation) forms a hydrogen bond. The differences in two positions between *CDC19* and *PYK2* (Table S12A) are not likely to explain why FBP does not regulate the activity of *PYK2*. FBP may still be bound to *PYK2*, but the enzyme activity may not be dependent on this binding, or the dependence is only very small; less than two times activation was observed by Boles et al. [61].

**S12.3. Active site**

Phosphoglycolate bound to the active site of *CDC19* in 1a3w is a structural analog of phosphoenolypyruvate. The crystal structure 1a3w contains also Mn^2+^ and K^+^ ions in the active site. The active site of *PYK2* is conserved. Away from the active site, H308 in *PYK2* is in the place of *CDC19* site Y306. In 1a3x structure, Y306 forms a hydrogen bond to the potentially catalytic K337, whereas no hydrogen bond can be formed after the in silico mutation Y306H (in Swiss-PdbViewer).

**S12.4. Dimerization site**

In the protein dimerization region, A387 is in *PYK2* in a place, in which *CDC19* has S385. In *CDC19*, the mutation S385P modifies the enzyme regulation making the enzyme to require FBP for activity [64]. *K. waltii* 6945 has alanine at this position. S385 makes important hydrogen bonds at the dimer interface [63].

**S12.5. Cellular localization**

Huh et el report cytoplasmic localization for *CDC19* and *PYK2* [6]. According to Yeast Protein Localization Server, *CDC19* is predicted to be localized to cytoplasm and *PYK2* to nucleus. *CDC19* prediction is in line with its location in cytoplasm and functioning in glycolysis. *PYK2* functions also in metabolism, and thus, the prediction may not give correct localization. However, Saccharomyces Genome Database ([http://db.yeastgenome.org/cgi-bin/locus.pl?locus=PYK2#summaryParagraph](http://db.yeastgenome.org/cgi-bin/locus.pl?locus=PYK2#summaryParagraph)) reports that *PYK2* is both cytosolic and mitochondrial.

**S12.6. Conclusions**

There may not be any major differences between these duplicated genes in the catalytic activity. The observed functional differences between *CDC19* and *PYK2* could be caused by small changes at or near the active site and FBP-binding site. They could also be related to the finding that *CDC19* and *PYK2* have differing charge properties as reflected in the differing theoretical pI (7.66 in *CDC19* and 6.90 in *PYK2*).

**S13. Supplemental data for *ADH1* and *ADH5***

Alcohol dehydrogenase is required for the reduction of acetaldehyde to ethanol, which is the last step in the glycolytic pathway. Yeast has several alcohol dehydrogenase genes: *ADH1*, *ADH2*, *ADH3* and *ADH5* form a highly similar group of genes [65, 66]. Identity with *ADH1* is 93% for *ADH2*, 80% for *ADH3* and 77% for *ADH5*. *ADH1* (*YOL086C* by systematic name) and *ADH5* (*YBR145W* by systematic name) are the genes that are derived from the genome duplication.

*ADH1* accounts for the major part of alcohol dehydrogenase activity in growing baker’s yeast (for review see [66]). While *ADH1* and *ADH2* are expressed in cytoplasm, *ADH3* is a mitochondrial form. *ADH2* is repressed by glucose and is mainly involved in ethanol consumption, converting ethanol into acetaldehyde. Mutation tests indicate that *ADH5* protein is able to produce ethanol [67, 68]. *ADH5* expression is increased in *S. cerevisiae* mutant able to grow anaerobically on xylose [62].

The yeast alcohol dehydrogenases have catalytic domain and coenzyme-binding domain [66]. The domains are separated by a cleft, which contains a deep pocket accommodating the substrate and the nicotinamide moiety of the coenzyme. Zinc is a catalytic metal located in the active site.

**S13.1. Modeling**

*ADH1* shows 42% identity with the crystallized *Pseudomonas aeruginosa* alcohol dehydrogenase (1llu). *ADH5* shows 45% identity with 1llu. Also other alcohol dehydrogenases have been crystallized. Model of *ADH1* was created for the amino acids region 2-346 by SWISS-MODEL. 1llu was used as the template. Model of *ADH5* was created for the region 6-348. 1llu contains NAD liganded to the binding site.

**S13.2. NAD-binding pocket**

On the basis of *P. aeruginosa* alcohol dehydrogenase structure (1llu), the NAD-binding pocket was identified in yeast genes. The pocket is highly conserved in Kw23198, *ADH1* and *ADH5* (Table S13A). Some minor differences were observed. Ser49 in ADH5 is in a position in which 1llu has Thr46; OG1 atom of Thr46 is in 2.83 Å distance from NO2 atom of NAD, and according to alignment of 1llu and the model of *ADH5*, OG atom of Ser49 can in principle be in conformation with about 3 Å distance to NAD. Therefore, it is likely that Ser49 does not have any major effect on NAD binding of *ADH5*.

In the turn region corresponding to 178-GIGG-181 of 1llu, the Kw23198, *ADH1* and *ADH5* proteins have one amino acid longer sequence. The residues of the sequence 178-GIGG-181 of 1llu is lining NAD. SWISS-MODEL modeled this turn region differently from the sequence alignment; Table S13A shows the structural alignment (residues Ala180 and Gly181 in *ADH1* and Cys183 and Gly184 in Kw23198 and *ADH5*). In the *ADH1* loop (178-GAAGG-182), according to structure modeling, Ala180 is the inserted amino acid, whereas in Kw23198 and *ADH5* it is Gly184. Because all yeast genes have the same number of residues in this region and *ADH5* resembles more *K. waltii* protein than *ADH1*, this apparently means that *ADH5* may have retained the original structure in this loop.

Cys298 in *ADH5* (Table S13) differs from the corresponding position in *ADH1* (Tyr295) and *K. waltii* (Tyr298). The same site is Ile291 in 1llu, in which the side chain is pointing away from NAD, and thus the difference in this position in *ADH5* is not likely to have any major functional consequences.

**________________________________________________________________________**

**Table S13. NAD-binding pocket in alcohol dehydrogenases.** NAD binding information is from 1llu. Key amino acids of the zinc-binding site are shown by stars above the sites.

* * *

1llu C44 H45 T46 H49 W55 H67 W93 C154 T158 S177

Kw23198 C47 H48 T49 H52 W58 H70 W96 C157 T161 S180

*ADH1* C44 H45 T46 H49 W55 H67 W93 C154 T158 S177

*ADH5* C47 H48 S49 H52 W58 H70 W96 C157 T161 S180

1llu G178 I179 - G180 - G181 L182 D201 I202 K206

Kw23198 G181 A182 - C183 G184 G185 L186 D205 G206 K210

*ADH1* G178 A179 A180 G181 - G182 L183 D202 G203 K207

*ADH5* G181 A182 - C183 G184 G185 L186 D205 G206 K210

1llu A221 R222 T243 A244 V245 S246 A249 V266 G267 L268

Kw23198 F225 T226 V249 S250 V251 S252 A255 V272 G273 L274

*ADH1* F222 T223 V246 S247 V248 S249 A252 V269 G270 M271

*ADH5* F225 T226 V249 S250 V251 S252 A255 V272 G273 M274

1llu I291 V292 M329 G332 R337

Kw23198 Y298 V299 M336 G339 R344

*ADH1* Y295 V296 M333 G336 R341

*ADH5* C298 V299 M336 G339 R344

**Fig. S13. Substrate-binding pocket in alcohol dehydrogenases.** Residues of the substrate-binding pocket (including sites coordinating the catalytic zinc) in horse liver alcohol dehydrogenase (*1qv6*) are shown in blue and *P. aeruginosa* alcohol dehydrogenase (*1llu*) in light blue. *1qv6* has mutations H51Q and K228R that are changed back to original amino acid residues in the alignment. The differences in *ADH5* when compared to *ADH1* are shown in red. The sites lining the pocket in *P. aeruginosa* alcohol dehydrogenase are C44, T46, H49, W55, H67, W93, Y120, C154, L268, I291 and V292, and in horse liver alcohol dehydrogenase they are C46, S48, H51, L57, H67, F93, L116, F140, L141, C174, V294 and I318.

Kw23198 MSAPEIPKTQKAVIFYENGGPLEYKDIPVPKPSATELLINVKYSGVCHTDLHAWKGDWPL 60

ADH1 MS---IPETQKGVIFYESHGKLEYKDIPVPKPKANELLINVKYSGVCHTDLHAWHGDWPL 57

ADH5 MPSQVIPEKQKAIVFYETDGKLEYKDVTVPEPKPNEILVHVKYSGVCH**S**DLHAWHGDWPF 60

1LLU MT---LPQTMKAAVVHAYGAPLRIEEVKVPLPGPGQVLVKIEASGV**C**H**T**DL**H**AAEGD**W**PV 57

1QV6 -STAGKVIKCKAAVLWEEKKPFSIEEVEVAPPKAHEVRIKMVATGI**C**R**S**DD**H**VVSGT**L**-- 57

Kw23198 PTKLPLVGGHEGAGVVVAMGENVKGWKIGDYAGIKWLNGSCMSCESCELSNESNCPEADL 120

ADH1 PVKLPLVGGHEGAGVVVGMGENVKGWKIGDYAGIKWLNGSCMACEYCELGNESNCPHADL 117

ADH5 QLKFPLIGGHEGAGVVVKLGSNVKGWKVGDFAGIKWLNGTCMSCEYCEVGNESQCPYLDG 120

1LLU KPPLPFIPG**H**EGVGYVAAVGSGVTRVKEGDRVGIP**W**LYTACGCCEHCLTGWETLCESQQN 117

1QV6 VTPLPVIAG**H**EAAGIVESIGEGVTTVRPGDKVIPL**F**TP-QCGKCRVCKHPEGNFCLKND**L** 116

Kw23198 ---------------------SGYTHDGSFQQYATADAVQAAKIPQGTDLAEVAPVLCAG 159

ADH1 ---------------------SGYTHDGSFQQYATADAVQAAHIPQGTDLAQVAPILCAG 156

ADH5 ---------------------TG**F**THDGTFQEYATADAVQAAHIPPNVNLAEVAPILCAG 159

1LLU ---------------------TG**Y**SVNGGYAEYVLADPNYVGILPKNVEFAEIAPIL**C**AG 156

1QV6 SMPRGTMQDGTSRFTCRGKPIHH**FL**GTSTFSQYTVVDEISVAKIDAASPLEKVCLIG**C**GF 176

Kw23198 ITVY-KALKSANLSAGDWVAISGACGGLGSLCIQYATAMG-YRVLGIDGGAEKAELFKQL 217

ADH1 ITVY-KALKSANLMAGHWVAISGAAGGLGSLAVQYAKAMG-YRVLGIDGGEGKEELFRSI 214

ADH5 ITVY-KALKRANVIPGQWVTISGACGGLGSLAIQYALAMG-YRVIGIDGGNAKRKLFEQL 217

1LLU VTVY-KGLKQTNARPGQWVAISG-IGGLGHVAVQYARAMG-LHVAAIDIDDAKLELARKL 213

1QV6 STGYGSAVKVAKVTQGSTCAVFG-LGGVGLSVIMGCKAAGAARIIGVDINKDRFAKAKEV 235

Kw23198 GGEVFIDFT-TCKDVEGEIIKATNGGAHGVINVSVSEAAIESSTRYVRAN-GTVVLVGLP 275

ADH1 GGEVFIDFT-KEKDIVGAVLKATDGGAHGVINVSVSEAAIEASTRYVRAN-GTTVLVGMP 272

ADH5 GGEIFIDFT-EEKDIVGAIIKATNGGSHGVINVSVSEAAIEASTRYCRPN-GTVVLVGMP 275

1LLU GASLTVNAR-QEDPVE--AIQRDIGGAHGVLVTAVSNSAFGQAIGMARRG-GTIALVG**L**P 269

1QV6 GATECVNPQDYKKPIQEVLTEMSNGGVDFSFEVIGRLDTMVTALSCCQEAYGVSVIVG**V**P 295

Kw23198 GGAKCRSDVFSHVVKSISIVGSYVG----NRADTREALDFFSRGLVKSP--IKVVGLSTL 329

ADH1 AGAKCCSDVFNQVVKSISIVGSYVG----NRADTREALDFFARGLVKSP--IKVVGLSTL 326

ADH5 AHAYCNSDVFNQVVKSISIVGS**C**VG----NRADTREALDFFARGLIKSP--IHLAGLSDV 329

1LLU PGD-FPTPIFDVVLKGLHIAGS**IV**G----TRADLQEALDFAGEGLVKAT--IHPGKLDDI 322

1QV6 PDSQNLSMNPMLLLSGRTWKGA**I**FGGFKSKDSVPKLVADFMAKKFALDPLITHVLPFEKI 355

Kw23198 PEVFEKMEKGQIVGRYVVDTSK

ADH1 PEIYEKMEKGQIVGRYVVDTSK

ADH5 PEIFAKMEKGEIVGRYVVETSK

1LLU NQILDQMRAGQIEGRIVLEM--

1QV6 NEGFDLLRSGESIRTILTF---

**S13.3. Substrate-binding pocket**

*P. aeruginosa* and horse liver alcohol dehydrogenases were used to analyse the substrate-binding pocket in yeasts (Fig. S13). There are some differences between these enzymes in the substrate pocket. Yeast *ADH1* and *ADH5* have higher similarity with *P. aeruginosa* than horse liver enzyme.

The sequence of *ADH5* in the potential substrate-binding pocket is quite conserved. There are two conserved differences between *ADH5* and *ADH1* in the substrate-binding pocket. The only bigger difference in ADH5 is the presence of Cys in position 298. The corresponding site has Tyr or Ile in the other enzymes in Fig. S13. However, these differences may not have any major effect.

The Zn-binding site (Cys-44, His-67 and Cys-154 in 1llu) near to NAD binding and the substrate-binding sites is fully conserved in the fast evolving gene, *ADH5* (Table S13A).

**S13.4. Cellular localization**

Predicted (Yeast Protein Localization Server) localization for *ADH1* is cytoplasm and for *ADH5* nucleus. Organelle Database reports cytosolic localization for *ADH1* (<http://organelledb.lsi.umich.edu/gene.php?sys_name=YOL086C>). Huh et al. report both cytoplasmic and nuclear localization for *ADH5*[6]*.*

**S13.4. Conclusions**

*ADH5* is functional and the sequence and the active site analyses indicate that the basic biochemical function is likely to be conserved, although some differences could exist either in regulation or activity.

**S14. Supplemental data for Glycyl-tRNA synthase genes *GRS1* and *GRS2***

*GRS1* (*YBR121C* by other name) and *GRS2* (*YPR081C* by other name) are 59% identical. Both have less than 30% identity with crystal structures of tRNA-synthetases, and adequate structural models were not obtained for them by automatic modeling.

Functional studies were reported by Turner et al. [69]:

*● GRS1* encodes both mitochondrial and cytoplasmic functions. *GRS2* is expressed only in very low amounts.

*●* A stable and active form of *GRS1* was isolated, whereas no stable form of *GRS2* was obtained.

*●* *GRS2* contains a long deletion at a charge-rich region that is a prominent distinguishing feature between *GRS1* and *GRS2* (see also Fig. S14). The charge-rich region is located within an active site subdomain that is predicted to contact the acceptor stem of the tRNA substrate. Functional consequence could be enhanced affinity or altered specificity for tRNA.

*●* *GRS2* protein cannot substitute for *GRS1* protein.

P552F mutation in *GRS1* affected the 3’-end formation and increased the readthrough of terminator [70].

**S14.1. Sequence features**

*GRS2* has Thr at the position corresponding to Pro-552 of *GRS1*, whereas other yeast proteins have Pro at the same position (see Fig S14). Although it has been suspected that *GRS2* is experiencing pseudogenization, it is noteworthy that *GRS2* has several absolutely conserved sequence regions throughout the protein (Fig. S14). This suggests protection by selection.

**S14.2. Cellular localization**

Huh et al. report cytoplasmic localization for *GRS1* and *GRS2* [6]. Yeast Protein Localization Server predicted cytoplasmic localization for *GRS1*. Predicted localization for *GRS2* is nuclear. Saccharomyces Genome Database reports cytoplasmic and mitochondrial localization for *GRS1*(<http://db.yeastgenome.org/cgi-bin/locus.pl?locus=GRS1>) and cytoplasmic location for *GRS2* (<http://db.yeastgenome.org/cgi-bin/locus.pl?locus=GRS2>).

**Fig. S14. Alignment of *GRS1*, *GRS2* and K. waltii 3922 protein with other corresponding yeast proteins.** The Pro-552 of *GRS1* is shown in bold and Thr at the same position in *GRS2* is shown in red. SYG_yeast is *GRS1* and SYG2_yeast is *GRS2*.

K. waltii 3922 MSVEEITQARRTVEFSRENLESVLKRRFFFAPSFELYGGVSGLYDYGPPG

Q6FTM3_CANGA MTVEDVKQARQAVEFSREKLESVLRGRFFYAPAFDLYGGVSGLYDYGPPG

Q6CVW3_KLULA MSVEEVQQAKKAVEFSRESLESVLKRRFFYAPAFELYGGVSGLYDYGPPG

Q75BD7_ASHGO MASEDVQLARKAVEFNRENLESVLKRRFFFAPAFELYGGVSGLYDYGPPG

SYG_YEAST MSVEDIKKARAAVPFNREQLESVLRGRFFYAPAFDLYGGVSGLYDYGPPG

Q6BQ74_DEBHA -----MSTSRTPIPFSRESLEQVLKRRFFFAPAFEIYGGVSGLYDYGPPG

Q5A2A5_CANAL -----MSASRTNIPFSRDSLEQTLKRRFFFAPSFEIYGGVAGLFDFGPPG

Q6C5W5_YARLI -----MSTRPADQELNRETLDAVLKRRFFYAPAFEIYDGVSGLYDYGPPG

SYG_SCHPO -----MTEVSKAAAFDRTQFEELMKKRFFFSPSFQIYGGISGLYDYGPPG

SYG2_YEAST --------MPLMSNSERDKLESTLRRRFFYTPSFEIYGGVSGLFDLGPPG

.* :: :: ***::*:*::*.*::**:* ****

K. waltii 3922 CAFQANVIDVWRKHFILEEDMLEVDCSMLTPYEVLKTSGHVDKFSDWMCR

Q6FTM3_CANGA CSFQANVVDQWRKHFILEEDMLEVDCTMLTPYEVLKTSGHVDKFSDWMCR

Q6CVW3_KLULA CSFQANIVDVWRKHFVLEEDMLEVDCTMLTPYEVLKTSGHVDKFSDWMCK

Q75BD7_ASHGO CAFQANIVDVWRKHFILEEDMLEVDCTMLTPYEVLKTSGHVDKFSDWMCQ

SYG_YEAST CAFQNNIIDAWRKHFILEEDMLEVDCTMLTPYEVLKTSGHVDKFSDWMCR

Q6BQ74_DEBHA CALQANIMDTWRKHFILEEDMLEVDCTMLTPHEVLKTSGHVDKFADWMCR

Q5A2A5_CANAL CAFQNNVIDAWRKHFILEEDMLEVEATMLTPHDVLKTSGHVDRFSDWMCK

Q6C5W5_YARLI CALQTRIIDTWRDHFVLEDDMLEVDTTMLTPHEVLKTSGHVDKFADWMCR

SYG_SCHPO SALQSNLVDIWRKHFVIEESMLEVDCSMLTPHEVLKTSGHVDKFADWMCK

SYG2_YEAST CQLQNNLIRLWREHFIMEENMLQVDGPMLTPYDVLKTSGHVDKFTDWMCR

. :* .:: **.**::*:.**:*: .****::*********:*:****:

K. waltii 3922 DLKTGEIFRADHLVEEVLEARLKGDQEARGLTKDANASAQDDADKKKRKK

Q6FTM3_CANGA DLKTGEIFRADHLVEEVLEARLKGDQEARGLVKDANAEAEEDADKKKRKK

Q6CVW3_KLULA DPKTGEIFRADHLVEEVLEARLKGDKEARGLATDANAEAEADAEKKKRKK

Q75BD7_ASHGO DPKSGEIFRADHLVEEVLEARLKGDKAARGISAAP---EEEDADKKKRKK

SYG_YEAST DLKTGEIFRADHLVEEVLEARLKGDQEARGLVEDANAAAKDDAEKKKRKK

Q6BQ74_DEBHA DLKTGEIFRADHLVEEVLEARLKGDKAARGVAINEGEE--EDADKKKRKK

Q5A2A5_CANAL DLKTGEIFRADHLVEEVLESRLKGDKLARGVKIVE--E--EDEDKKKRKK

Q6C5W5_YARLI DLASGEIFRADHLVEEVLEARLKGDKEARG--IK--EDVVEDESAKKRKK

SYG_SCHPO DPATGEIFRADHLVEEVLEARLKGDKEARGQNSN--DQPEESDDKKKRKK

SYG2_YEAST NPKTGEYYRADHLIEQTLKKRLLDKDVN----------------------

: :** :*****:*:.*: ** ...

K. waltii 3922 KVKEIKAVKLDDNVVKEYEEVLAKIDGYSGQELGELMVKYNIGNPVTGET

Q6FTM3_CANGA KVKQIKAVKLEDDVVKEYQHILAQIDGYSGPELGEMMKKYNIGNPVTGEP

Q6CVW3_KLULA KVKEIKAIKLDDAVVQEYEQILAKIDGYSGAELGELMVKYDIGNPVSGDK

Q75BD7_ASHGO KVKQIKAEKLDDSVIQEYESVLAKIDGYSGEELGELMVKFNIGNPVTGET

SYG_YEAST KVKQIKAVKLDDDVVKEYEEILAKIDGYSGPELGELMEKYDIGNPVTGET

Q6BQ74_DEBHA KVKEIKSIKLDDEVVKEYENVLAQIDGYSGSQLGELMTKYKINNPATDGP

Q5A2A5_CANAL KVKEIKNVKLEDEVVKEYESILAQIDGFSGPQLGELIVKYDITNPSTGGK

Q6C5W5_YARLI KVKEIVAIKLDDNVKEEYETILAKIDGFSGPELGEIMDKYKIVNPVTGGP

SYG_SCHPO KVKEIRATRLDDKTVEEYEFILAQIDNYDGDQLGELMKKYDIRNPATNGE

SYG2_YEAST -----------PQDMKNMEKILTTIDGFSGPELNLVMQEYNINDPVTNDV

:: : :*: **.:.* :*. :: ::.* :* :.

K. waltii 3922 LEPPKAFNLMFETAIGPSGQLKGYLRPETAQGQFLNFNKLLEFNNHKTPF

Q6FTM3_CANGA LEPPMAFNLMFETAIGPSGQLKGYLRPETAQGQFLNFNKLLEFNNGKTPF

Q6CVW3_KLULA LEPPRAFNLMFETAIGPSGQYKGYLRPETAQGQFLNFNKLLEFNNGKTPF

Q75BD7_ASHGO LEPPKAFNLMFETAIGPSGQLKGYLRPETAQGQFLNFNKLLEFNNGKTPF

SYG_YEAST LESPRAFNLMFETAIGPSGQLKGYLRPETAQGQFLNFNKLLEFNNSKTPF

Q6BQ74_DEBHA LELPIEFNLMFETAIGPSGQLKGFLRPETAQGQFLNFSKLLDCNNEKMPF

Q5A2A5_CANAL LEPPVEFNLMFDTAIGPSGNLKGYLRPETAQGQFLNFNKLLEFNNDKMPF

Q6C5W5_YARLI LEKPMEFNLMFETAIGPSGKLKGFLRPETAQGQFLNFNKLLDCNNTKMPF

SYG_SCHPO LETPRQFNLMFETQIGPSGGLKGYLRPETAQGQFLNFSRLLEFNNGKVPF

SYG2_YEAST LDALTSFNLMFETKIGASGQLKAFLRPETAQGQFLNFNKLLEINQGKIPF

*: *****:* **.** *.:*************.:**: *: * **

K. waltii 3922 ASASIGKSFRNEISPRSGLLRVREFLMAEIEHFVDPLNKTHPRFNDVKDI

Q6FTM3_CANGA ASASIGKSFRNEISPRAGLLRVREFLMAEIEHFVDPLDKSHPKFHEVKDI

Q6CVW3_KLULA ASASIGKSFRNEISPRSGLLRVREFLMAEIEHFVDPNDKSHKRFQDIKDI

Q75BD7_ASHGO ASASIGKSFRNEISPRSGLLRVREFLMAEIEHFVDPENKNHPRFDEVKNL

SYG_YEAST ASASIGKSFRNEISPRAGLLRVREFLMAEIEHFVDPLDKSHPKFNEIKDI

Q6BQ74_DEBHA ASASIGKSFRNEISPRAGLLRVREFLMAEIEHYVDPDNKSHSRFDEIKDL

Q5A2A5_CANAL ASASIGKSFRNEIAPRAGLLRVREFLMAEIEHYVDPESKSHPKFEDVKDI

Q6C5W5_YARLI ASASIGKSFRNEISPRSGLLRVREFTMAEIEHFVDPLDKDHHRFDEVKDV

SYG_SCHPO ASAMVGKAFRNEISPRSGLLRVREFLMAEVEHFVDPKNKEHDRFDEVSHM

SYG2_YEAST ASASIGKSFRNEISPRSGLLRVREFLMAEIEHFVDPLNKSHAKFNEVLNE

*** :**:*****:**:******** ***:**:*** .* * :*.:: .

K. waltii 3922 KLKFLPREVQQSG-STEPVESTIGDAVATKMVDNETLGYFIARIYTFLIT

Q6FTM3_CANGA KLSFLPRNIQQSG-STEPLVTTIGEAVASKMVDNETLGYFIARIYLFLIK

Q6CVW3_KLULA KLKFLPREVQQSG-STVPLEKTVGEAVATKLVDNETLGYFIARIYQFLIK

Q75BD7_ASHGO KLKFLPKGVQEAG-RTEPIESTVADAVASGMIDNQTLGYFIARIYQFLTK

SYG_YEAST KLSFLPRDVQEAG-STEPIVKTVGEAVASRMVDNETLGYFIARIYQFLMK

Q6BQ74_DEBHA KLKFLPKGVQESG-SNELTEKSLGEAVSSGMVDNETLGYFLARIYSFLIK

Q5A2A5_CANAL KLKFLPKNVQESG-STELIEESIGKAVSSGMVDNETLGYFIARIYLFLVK

Q6C5W5_YARLI KLRFLAKDVQSAG-KTDIQEMTIGQAVETGLVDNKTLGYFLARIYLFLIK

SYG_SCHPO PLRLLPRGVQLEG-KTDILEMPIGDAVKKGIVDNTTLGYFMARISLFLEK

SYG2_YEAST EIPLLSRRLQESGEVQLPVKMTIGEAVNSGMVENETLGYFMARVHQFLLN

: :*.: :* * .:..** . :::* *****:**: ** .

K. waltii 3922 IGVDPTKLRFRQHMANEMAHYAADCWDAELHTSYGWIECVGCADRSAYDL

Q6FTM3_CANGA IGVDDTKLRFRQHMANEMAHYAADCWDAELKTSFGWIECVGCADRSAYDL

Q6CVW3_KLULA IGVDPERLRFRQHMANEMAHYAADCWDAELQTSYGWIECVGCADRSAYDL

Q75BD7_ASHGO IGVDEEKLRFRQHMSNEMAHYATDCWDAELKTSYGWIECVGCADRSAYDL

SYG_YEAST IGVDESKLRFRQHMANEMAHYAADCWDGELKTSYGWIECVGCADRSAYDL

Q6BQ74_DEBHA IGVDPSRLRFRQHMSNEMAHYAADCWDAELHTSYGWIECVGCADRSAYDL

Q5A2A5_CANAL IGVDTNRLRFRQHMSNEMAHYASDCWDAELETSYGWIECVGCADRSAYDL

Q6C5W5_YARLI IGVNPDRLRFRQHMSNEMAHYATDCWDAELHTSYGWIECVGCADRSAYDL

SYG_SCHPO IGIDMNRVRFRQHMSNEMAHYACDCWDAEIQCSYGWIECVGCADRSAYDL

SYG2_YEAST IGINKDKFRFRQHLKNEMAHYATDCWDGEILTSYGWIECVGCADRAAFDL

**:: :.*****: ******* ****.*: *:***********:*:**

K. waltii 3922 TVHANKTKEKLVVRQKLEEPVQVTKWEIELTKKLFGPKFRKDAPKVEAFL

Q6FTM3_CANGA TVHANKTKEKLVVRQKLETPVEVTKYEIDLTKKLFGPKFRKDAPKVEAYL

Q6CVW3_KLULA TVHSNKTKEKLVVREALETPIEVTKWEATLVKKLFGPKFRKDAPKVEARL

Q75BD7_ASHGO TVHANKTKTALVVREKLDVPRQVTQWEIELTKKLFGPKFRKDAPKVENYL

SYG_YEAST TVHSKKTKEKLVVRQKLDNPIEVTKWEIDLTKKLFGPKFRKDAPKVESHL

Q6BQ74_DEBHA SVHSARTNEKLVVRQPLPEPVLVEKYEVNIAKKKFGPKFRKDAGTVENWL

Q5A2A5_CANAL SVHSARTGEKLVARQTLAEPRTVENFEIEIAKKKFGPKFRKDAGTVEKWL

Q6C5W5_YARLI SVHEARTKVKLQVQQKLDAPLVEDKFVCEYDKKKFGPLLKKAAKPVEEWF

SYG_SCHPO SVHSKATKTPLVVQEALPEPVVVEQFEVEVNRKKFGPRFKRDAKAVEEAM

SYG2_YEAST TVHSKKTGRSLTVKQKLDTPKERTEWVVEVNKKFFGSKFKQKAKLIESVL

:** * * .:: * * :: :* **. ::: * :* :

K. waltii 3922 LGLSQEELESKAKDLKDAGKISFEVEGMDG-QIELDDKFLSIEQVTRTEH

Q6FTM3_CANGA TELSQEELEKKAEELKTNGKIVFTVKGIEG-EIELDDKFVVIEKRTKVEH

Q6CVW3_KLULA LAFSQEELESYSAQLKKDGKITLKVEGMEG-DVEVDDKMVSIEKVTNTEH

Q75BD7_ASHGO LNLSQDELASKAEQLSSDGKIVFQVEGIEG-DIELDSKFISIEHKTKTEH

SYG_YEAST LNMSQDDLASKAELLKANGKFTIKVDGVDG-EVELDDKLVKIEQRTKVEH

Q6BQ74_DEBHA LARTQCELEDLCKELNENNKIVFKIDSIPN-SIELDTEFVKIEKVKRTEH

Q5A2A5_CANAL TSRTQCELEELGKELSEKGKIVVQIKGVEG-DVELDGDLIKIDKVKRTEH

Q6C5W5_YARLI ESRTQCELEDLAKALEAGKIVLPEIEGVEVAGTELDKSHIKIEKKTITTH

SYG_SCHPO ISWPESEKVEKSAQLVAEGKIIVNVNGVEHT---VESDLVTIEKRKHTEH

SYG2_YEAST SKFSQDELIRRHEELEKNGEFTCQVN---GQIVKLDSSLVTIKMKTTLQH

.: : * . :. :: . : *. . *

K. waltii 3922 VREFVPNVIEPSFGIGRIIYAVFEHAFWSRPEDTA--RAVLSFPPLVAPT

Q6FTM3_CANGA VREFVPNVIEPSFGIGRIIYSIFEHSFWSRPEDTA--RAVLSFPPLVAPT

Q6CVW3_KLULA IREFVPNVIEPSFGIGRIIYSIFEHSFWSRPEDTA--RAVLSFPPLVAPT

Q75BD7_ASHGO VREYVPNVIEPSFGIGRIIYAIFEHSFWSRPEDAA--RSVLSFPPLVAPT

SYG_YEAST VREYVPSVIEPSFGIGRIIYSVFEHSFWNRPEDNA--RSVLSFPPLVAPT

Q6BQ74_DEBHA IREFTPNVIEPSFGIGRILYSIFEHQFWARPEDKD--RTVLSLPPLVAPT

Q5A2A5_CANAL VREFVPNVIEPSFGIGRILYSIFEHQFWCRPDDAD--RGVLSLPPIVAPT

Q6C5W5_YARLI VRDYTPNVIEPSFGIGRILYSLIEHCFWTRPEDASGAKGVLSFPPRIAPT

SYG_SCHPO IRTYTPNVIEPSFGLGRILYVLMEHAYWTRPEDVN--RGVLSFPASIAPI

SYG2_YEAST IREYIPNVIEPSFGLGRIIYCIFDHCFQVRVDSES--RGFFSFPLQIAPI

:* : *.*******:***:* :::* : * :. : .:*:* :**

552

K. waltii 3922 KVLLVPLSNHPDLSSVAQEVSKVFRKEKIPFKVDDSGVSIGKRYSRNDEL

Q6FTM3_CANGA KVLLVPLSNHKDLAPVTAQVSKILRKEQIAFRVDDSGVSIGKRYARNDEL

Q6CVW3_KLULA KVLLVPLLNNPELSKITAQVSQILRKEQIPFKVDESGVSIGKRYARNDEL

Q75BD7_ASHGO KVLLVPLSNNADLAEVVTEVSRVLRKEQIPFKVDDSGVSIGKRYARNDEL

SYG_YEAST KVLLV**P**LSNHKDLVPVHHEVAKILRKSQIPFKIDDSGVSIGKRYARNDEL

Q6BQ74_DEBHA KVLLVPLSSNAELQPIVKKISAFLRKEQVPFKVDDSSASIGKRYARNDEL

Q5A2A5_CANAL KVLLVPLSNNSELQPIVKKVSQALRKEKIPFKVDDSSASIGKRYARNDEL

Q6C5W5_YARLI KVLVVPLSSQKELAPFTQEVSKKLRQARISAKVDDSSASIGKRYARNDEM

SYG_SCHPO KALIVPLSRNAEFAPFVKKLSAKLRNLGISNKIDDSNANIGRRYARNDEL

SYG2_YEAST KVFVT**T**ISNNDGFPAILKRISQALRKREIYFKIDDSNTSIGKKYARNDEL

*.::..: : : . .:: :*: : ::*:*...**::*:****:

K. waltii 3922 GTPFGVTIDFESAKDGTVTLRERDSTKQVRGSVKDVVKAIRDITYN--GV

Q6FTM3_CANGA GTPFGITIDFDSVKDGSVTLRERDSTKQVRGSVEAVIKAVREITYN--GA

Q6CVW3_KLULA GTPFGVTIDFDSVTDGSITLRERDSTKQVRGSVADVIKAIREITYQ--GV

Q75BD7_ASHGO GTPFGITIDFESIKDGSVTLRERDSTRQVRGSVTDIIRAIRDITYN--GV

SYG_YEAST GTPFGVTIDFESAKDHSVTLRERDSTKQVRGSVENVIKAIRDITYN--GA

Q6BQ74_DEBHA GTPFGITIDFDSVKDESVTLRDRDSTKQVRGSLEDIVEAIKDIAYN--NV

Q5A2A5_CANAL GTPFGITIDFDSVKDDSVTLRERDSTKQVRGSIQEIVEAIKDITYN--DG

Q6C5W5_YARLI GTPFGITVDFDTVKDNSVTLRERDSTRQVRGSIDAVIAAINVMTAD--DV

SYG_SCHPO GTPFGLTVDFETLQNETITLRERDSTKQVRGSQDEVIAALVSMVEG--KS

SYG2_YEAST GTPFGITIDFETIKDQTVTLRERNSMRQVRGTITDVISTIDKMLHNPDES

*****:*:**:: : ::***:*:* :****: :: :: :

K. waltii 3922 TWDEGTQSLKPFVSQSE------

Q6FTM3_CANGA SWEEGTKDLAPFVSQSDAE----

Q6CVW3_KLULA SWEEGTKDLAPFNSQAESE----

Q75BD7_ASHGO TWEEGTKSLTPFVSQSE------

SYG_YEAST SWEEGTKDLTPFIAQAEAEAETD

Q6BQ74_DEBHA SWTDGTSKLTPFDSQSEA-----

Q5A2A5_CANAL TWEEGTAKLKPFEGQSA------

Q6C5W5_YARLI AWEEATKDLTPFDSTDKE-----

SYG_SCHPO SFEDALAKFGEFKSTQE------

SYG2_YEAST DWDKSTFGLSPVKI---------

: .. : .

**S14.3. Conclusions**

The role of *GRS2* is unclear. It is still possible that *GRS2* is not experiencing pseudogenization, since the dN/dS ratio of 0.329 (when compared to *K. waltii* 3922) and the presence of conserved sequence regions that may indicate a protection by selection.

**S15. Supplemental data for *ERV14* and *ERV15***

**S15.1. Function and cellular localization**

*ERV14* protein (*YGL054C* by systematic name) is an integral membrane protein that functions as a cargo receptor, which cycles between the endoplasmic reticulum and Golgi. *ERV14* protein is localized to COPII-coated vesicles. It is involved in vesicle formation and incorporation of specific secretory cargo [71, 72]. Huh et al. report that ERV14 is localized to endoplasmic reticulum and vacuoles [6].

The functional information for the ohnolog *ERV15* (*YBR210W* by systematic name) is scarce. *ERV15* is 61.5 % identical to *ERV14*. *ERV15* protein cannot substitute for *ERV14* protein as a cargo receptor for transmembrane secretory protein Axl2p in yeast budding [72]. Unlike *ERV14*, *ERV15* does not affect the localization of yeast cis-Golgi protein *Rud3p* [73]. However, it was observed recently that overexpression of *ERV15* largely suppressed the sporulation defect in erv14-deletion cells. Although deletion of *ERV15* alone had no phenotype, erv14-erv15 double mutant displayed a complete block of prospore membrane formation [74]. Thus it is likely that *ERV15* has retained partially the function of the ancestral gene having lost the function in budding while retaining the function in sporulation.

*ERV14* and *ERV15* have three predicted transmembrane domains, which are amino acids 8-36, 45-69 and 103-126 in *ERV14* and 4-32, 46-72 and 100-128 in *ERV15* (<http://db.yeastgenome.org/cgi-bin/seqTools>). The aminoterminus of *ERV14* protein is located in the cytoplasm and carboxyterminus is located in the ER lumen [72]. Residues 97–101 on the cytoplasmic side of *ERV14* are critical for the recruitment of *ERV14* protein into *COPII* vesicles and for association with subunits of the *COPII* coat.

**S15.2. Sequence analysis**

Alignment of yeast and Aspergillus *ERV14*-like proteins (Fig. S15) shows that the site important for *COPII* interaction (position 97-101) differs at one position in *ERV15* from other yeasts, which generally have Lys at this position (position 100 in yeast *ERV14* numbering; except *S. pombe* has Gln). However, Aspergillus proteins also have Lys, and thus, it is not clear what is the functional role of this mutation in *ERV15*. It might be possible that the interaction contact at positions 97-101 of *ERV14*-like proteins in yeasts is different than in Aspergilli, and if this is the case, then Erv15 might have problems or differing mode in the interaction with *COPII*. Close to this site, *ERV15* has two unique cysteines that could form a disulphide bridge and thus change the local structure in the *COPII*-binding region. These cysteines are not found in the protein family shown in Table S15.

A significant difference in theoretical pI between *ERV14* (pI 6.93) and *ERV15* (pI 8.04) also reveals that the gene duplicates could be functionally diverging from each others.

**S15.3. Conclusions**

There are sequence features that appear to reflect functional divergence although their relationship to experimentally observed differences is not yet clear.

**Fig S15. Alignment of *ERV14* with similar yeast and Aspergillus proteins.** The postulated cytoplasmic loops of *ERV14* are shown in blue and loops located in ER lumen are shown in green. The site (97-101) in *ERV14* critical for *COPII* interaction is shown in bold. This data is from Powers and Barlowe, [72](See Fig 7). The differing positions in several proteins in the motif at 97-101 are shown in red. The two cysteines in *ERV15* close to the 97-101 region are shown by light blue.

Yeasts

ERV14_YEAST -------MGAWLFILAVVVNCINLFGQVHFTILYADLEADYINPIELCSK

K. waltii 1862 -------MAVWLFVLAVVLNCVNLFAQVHFTILYADLEADYINPIELCSK

Q6CUE6_KLULA -------MGVWLFIFAVIANCVNLFAQVHFTILYADLEADYINPIELCSK

Q6FR72_CANGA -------MGSYLFILAVVVNCINLFGQVHFTILYADLEADYINPIELCSK

Q75EC5_ASHGO -------MGAWLFVFAFVMNAVSMFLQVHFTIMYADLEADYVNPIELCSK

Q5ADQ4_CANAL --------------------------------MYSDLECDYINPIELCNK

Q9P6K6_SCHPO MSFVSWGSLNYLAYTFYRLNGANMLLQIFCVIMFSDLEMDYINPIDLCNK

ERV15_YEAST ----MSGTGLSLFVTGLILNCLNSICQIYFTILYGDLEADYINSIELCKR

Aspergilli

Q0CRI0_ASPTE -----MSGEAWLYLLAVLINAVNLFLQVFFTIMYSDLECDYINPIDLCNR

Q2UPN6_ASPOR -----MSGEAWLYLLAVLINAVNLFLQVFFTIMYSDLECDYINPIDLCNR

A1CT18_ASPCL -----MSGEAWLYLLAVLINAVNLFLQVFFTIMYSDLECDYINPIDLCNR

Q4WN80_ASPFU -----MSGEAWLYLLAVLINAVNLFLQVFFTIMYSDLECDYINPIDLCNR

Q5B2N5_EMENI -----MSGEAWLYLLAVLINAVNLFLQVFFTIMYSDLECDYINPIDLCNR

ERV14_YEAST VNKLITPEAALHGALSLLFLLNGYWFVFLLNLPVLAYNLNKIYNKVQLLD

K. waltii 1862 VNKLITPEALLHGVISLMFLLSGYWFVFLINLPLFAFNVNKHYKKLQLLD

Q6CUE6_KLULA VNKLILPEAALHGFISLLFLLNGYWFVFLLNLGILAYNGNKFYKKQQLLD

Q6FR72_CANGA VNKLIVPEAALHAVVSLLMLLNGYWFVFLLNLPVLAYNANKFYNKIQLLD

Q75EC5_ASHGO VNRLITPEAGVHAFISLLFLLNGYWFVFLLNLPVLFYNAKKIYHKMQLLD

Q5ADQ4_CANAL LNPWFIPEAGLHGFITVLFLINGYWFCFLLNLPLFAYNANKFYNKNHLLD

Q9P6K6_SCHPO LNDLVMPEIISHTLVTLLLLLGKKWLLFLANLPLLVFHANQVIHKTHILD

ERV15_YEAST VNRLSVPEAILQAFISALFLFNGYWFVFLLNVPVLAYNASKVYKKTHLLD

Q0CRI0_ASPTE LNAYIVPEAAVHAFLTLLFLINGYWLAIILNLPLLAFNAKKIYDNQHLLD

Q2UPN6_ASPOR LNAYIIPEAAVHAFLTFLFVINGYWLAILLNLPLLAFNAKKIYDNAHLLD

A1CT18_ASPCL LNAYIIPEAAVHAFLTTLFLINGYWLALILNLPLLAFNAKKIFENQHLLD

Q4WN80_ASPFU LNAYIIPEAAVHAFLTILFLINGYWLALILNLPLLAFNAKKILDNQHLLD

Q5B2N5_EMENI LNAYIIPEAGVHAFLTFLFVINGYWLAIALNLPLLAFNAKKIYDNQHLLD

97 101

ERV14_YEAST ATE**IFRTL**GKHKRESFLKLGFHLLMFFFYLYRMIMALIAESGDDF-

K. waltii 1862 ATEIFRTLGKHKKESFLKLGFYLLMFFFYLYRMIMALIAESD----

Q6CUE6_KLULA ATEIFRTLGKHKRESFIKLAFYLFLFFFYLYRMIMSLIAASE----

Q6FR72_CANGA ATEIFRTLGKHKRESFLKLGFYLLMFFFYLYRMIMALIADSED---

Q75EC5_ASHGO ATEIFRTLSKHKRESFLKLGFYLLLFFFYLYRMIMALIAEDN----

Q5ADQ4_CANAL ATEIFRTLSKHKKESFLKLGFHLLLFFFYLYRMIMALVNDEQ----

Q9P6K6_SCHPO ATEIFRQLGRHKRDNFIKVTFYLIMFFTLLYCMVMSLIQEE-----

ERV15_YEAST ATDIFRKLGR**C**KIE**C**FLKLGFYLLIFFFYFYRMVTALLENDANLIS

Q0CRI0_ASPTE ATEIFRKLNVHKKESFIKLGFHLLMFFFYLYSMIVALIRDESH---

Q2UPN6_ASPOR ATEIFRKLNVHKKESFIKLGFHLLMFFFYLYSMIVALIRDESH---

A1CT18_ASPCL ATEIFRKLNVHKKESFIKLGFHLLMFFFYLYSMIVALIRDDSN---

Q4WN80_ASPFU ATEIFRKLNVHKKESFIKLGFHLLMFFFYLYSMIVALIRDESH---

Q5B2N5_EMENI ATEIFRKLNVHKKESFIKLGFHLLMFFFYLYSMIVALIRDESH---

**S16. Supplemental data for *FEN1* and *ELO1***

**S16.1. Function and cellular localization**

De novo fatty acid synthesis uses acetyl-CoA as primer and fatty acid elongation uses longer-chain acyl-CoAs as primers. At least three different yeast elongases have been detected in yeast ([75] for review see [76]). Of these, *FEN1* and *ELO1* form a duplicated pair. They are 59% identical. *ELO1* is over 30 amino acids shorter at the C-terminus when compared to *FEN1* and almost 30 amino acids shorter when compared to *K. waltii* 13644.

*FEN1* (also *ELO2*, *GNS1* and *VBM2,* and *YCR034W* by systematic names) is involved in sphingolipid biosynthesis and acts on fatty acids of up to 24 carbons in length. *ELO1* (*YJL196C* by systematic name) is a medium-chain acyl elongase, and catalyzes carboxy-terminal elongation of unsaturated C12-C16 fatty acyl-CoAs to C16-C18 fatty acids. Elongase III synthesizes 20-26-carbon fatty acids from C18-CoA primers [75]. *FEN1* and *ELO1* proteins are localized to endoplasmic reticulum [6, 76].

**S16.2. Sequence analysis**

*FEN1* has seven and *ELO1* has only five predicted transmembrane domains (<http://db.yeastgenome.org/cgi-bin/seqTools>), although it is not fully ruled out that there could not be seven in *ELO1* (see Fig. S16). There is no structural information available. *ELO1* has close to 20 such sequence differences to *FEN1* and *K. waltii* 13644 that change charge properties (Fig. S16): *ELO1* contains over two times more such sites than *FEN1* and *K. waltii* 13644 altogether (shown in Fig. S16). These sites are located mostly outside the predicted transmembrane domains, but some are located also in the transmembrane domains. It could be that mutations changing local charge properties affect the interactions of hydrophobic fatty acids. Overally, the pI of *ELO1* (10.2) is not much different from *FEN1* (10.35). A long C-terminal deletion (~30 amino acids; affects C-terminal charges) might also affect the functional properties of *ELO1,* as well as the differences in the positioning of the predicted transmembrane domains (see Fig. S16).

**Fig. S16.** **The predicted transmembrane domains in *FEN1* and *ELO1***. Predicted transmembrane domains (obtained from SGD) are shown in bold. The differences changing strongly the local charge properties in *FEN1*, *ELO1* and Kw13644 are shown in red (one differing from the two others).

Kw13644 MLSIVQAQVATILNKYPCLAEFYPTLDRPFFNISLWENFDRAVANATKGHFIPSEFQFTP

FEN1 MNSLVTQYAAPLFERYPQLHDYLPTLERPFFNISLWEHFDDVVTRVTNGRFVPSEFQFIA 60

ELO1 MVS---DWKNFCLEK---ASRFRPTIDRPFFNIYLWDYFNRAVGWATAGRFQPKDFEFTV

:* ::: : **::****** **: *: .* .* *:* *.:*:*

Kw13644 GELPLSELPQVVAAITTYYVVVFGGRWLLQKSQPLKLNFLFQLHNLFLTSLSLTLLVLMV

FEN1 GELPLST**LPPVLYAITAYYVIIFGGRFLLS**KSKPFK**LNGLFQLHNLVLTSLSLTLLLLM**V 120

ELO1 GKQPLSEPR**PVLLFIAMYYVVIFGGRSLV**KSCKPLKLRFIS**QVHNLMLTSVSFLWLILMV**

*: *** *: *: ***::**** *:...:*:**. : *:***.***:*: *:***

Kw13644 EQLVPLIARNGLYFAICNLGAWTQPMVTLYYMNYITKYIEFIDTLFLVLKHKNLRFLHTY

FEN1 EQ**LVPIIVQHGLYFAICNIGAW**TQPL**VTLYYMNYIVKFIEFIDTFFLVL**KHKKLTFLHTY 180

ELO1 **EQML**PIVYRHGLYFAVCNVESWTQPMETLYYLNYMTKFVEFADTVLMVLKHRKLTFLHTY

**::*:: ::*****:**: :****: ****:**:.*::** **.::****::* *****

Kw13644 HHGATALLCYTQLVGTTAISWVPISLNLGVHVVMYWYYFLAARGIRVWWKEWVTRFQIIQ

FEN1 HHGATALLCYTQLMGT**TSISWVPISLNLGVHVVMYWYYF**LAARGIRVWWKE**WVTRFQIIQ** 240

ELO1 HHGATALLCYNQLVGY**TAVTWVPVTLNLAVHVLMYWYYF**LSASGIRVWWKAWVTRLQI**VQ**

**********.**:* *:::***::***.***:*******:* ******* ****:**:*

Kw13644 FILDIGFIYFAVYQKVSHLYFP--ELPHCGDCVGSTTATFSGCAIISSYLFLFVAFYIEV

FEN1 **FVLDIGFIYFAVYQ**KAVHLYFP--ILPHCGDCVGS**TTATFAGCAIISSYLVLFISFYI**NV 298

ELO1  **FMLDLIVVYYVLYQKIVAAYF**KNACTPQCEDCLGS**MTAIAAGAAILTSYLFLFISFYI**EV

*:**: .:*:.:*** ** *:* **:** ** :*.**::***.**::***:*

Kw13644 YRRRGTKKSRIVKRVRGGVAAKVNEYVNVDVAHTSTPSPSP----ARK-

FEN1 YKRKGTKTSRVVKRAHGGVAAKVNEYVNVDLKNVPTPSPSPKPQHRRKR 347

ELO1 YKRGSASGKKKINKNN---------------------------------

*:* .:. .: ::: .

**S16.3. Conclusions**

Since the fast evolving ohnolog *ELO1* elongates shorter fatty acids than the slow evolving *FEN1*, one could expect that *ELO1* has accumulated a mutation(s) that prevent the binding of long fatty acids.

**References**

1. Geisler M, Wilczynska M, Karpinski S, Kleczkowski LA: **Toward a blueprint for UDP-glucose pyrophosphorylase structure/function properties: homology-modeling analyses**. *Plant Mol Biol* 2004, **56**(5):783-794.

2. Katsube T, Kazuta Y, Tanizawa K, Fukui T: **Expression in Escherichia coli of UDP-glucose pyrophosphorylase cDNA from potato tuber and functional assessment of the five lysyl residues located at the substrate-binding site**. *Biochemistry* 1991, **30**(35):8546-8551.

3. Kellis M, Birren BW, Lander ES: **Proof and evolutionary analysis of ancient genome duplication in the yeast Saccharomyces cerevisiae**. *Nature* 2004, **428**(6983):617-624.

4. Valencia-Burton M, Oki M, Johnson J, Seier TA, Kamakaka R, Haber JE: **Different mating-type-regulated genes affect the DNA repair defects of Saccharomyces RAD51, RAD52 and RAD55 mutants**. *Genetics* 2006, **174**(1):41-55.

5. Lee J, Godon C, Lagniel G, Spector D, Garin J, Labarre J, Toledano MB: **Yap1 and Skn7 control two specialized oxidative stress response regulons in yeast**. *J Biol Chem* 1999, **274**(23):16040-16046.

6. Huh WK, Falvo JV, Gerke LC, Carroll AS, Howson RW, Weissman JS, O'Shea EK: **Global analysis of protein localization in budding yeast**. *Nature* 2003, **425**(6959):686-691.

7. Grandori R, Carey J: **Six new candidate members of the alpha/beta twisted open-sheet family detected by sequence similarity to flavodoxin**. *Protein Sci* 1994, **3**(12):2185-2193.

8. Ho Y, Gruhler A, Heilbut A, Bader GD, Moore L, Adams SL, Millar A, Taylor P, Bennett K, Boutilier K *et al*: **Systematic identification of protein complexes in Saccharomyces cerevisiae by mass spectrometry**. *Nature* 2002, **415**(6868):180-183.

9. Shero JH, Hieter P: **A suppressor of a centromere DNA mutation encodes a putative protein kinase (MCK1)**. *Genes Dev* 1991, **5**(4):549-560.

10. Lim MY, Dailey D, Martin GS, Thorner J: **Yeast MCK1 protein kinase autophosphorylates at tyrosine and serine but phosphorylates exogenous substrates at serine and threonine**. *J Biol Chem* 1993, **268**(28):21155-21164.

11. Neigeborn L, Mitchell AP: **The yeast MCK1 gene encodes a protein kinase homolog that activates early meiotic gene expression**. *Genes Dev* 1991, **5**(4):533-548.

12. Kassir Y, Rubin-Bejerano I, Mandel-Gutfreund Y: **The Saccharomyces cerevisiae GSK-3 beta homologs**. *Curr Drug Targets* 2006, **7**(11):1455-1465.

13. Brazill DT, Thorner J, Martin GS: **Mck1, a member of the glycogen synthase kinase 3 family of protein kinases, is a negative regulator of pyruvate kinase in the yeast Saccharomyces cerevisiae**. *J Bacteriol* 1997, **179**(13):4415-4418.

14. Rayner TF, Gray JV, Thorner JW: **Direct and novel regulation of cAMP-dependent protein kinase by Mck1p, a yeast glycogen synthase kinase-3**. *J Biol Chem* 2002, **277**(19):16814-16822.

15. Bax B, Carter PS, Lewis C, Guy AR, Bridges A, Tanner R, Pettman G, Mannix C, Culbert AA, Brown MJ *et al*: **The structure of phosphorylated GSK-3beta complexed with a peptide, FRATtide, that inhibits beta-catenin phosphorylation**. *Structure* 2001, **9**(12):1143-1152.

16. Jiang W, Koltin Y: **Two-hybrid interaction of a human UBC9 homolog with centromere proteins of Saccharomyces cerevisiae**. *Mol Gen Genet* 1996, **251**(2):153-160.

17. Frame S, Cohen P, Biondi RM: **A common phosphate binding site explains the unique substrate specificity of GSK3 and its inactivation by phosphorylation**. *Mol Cell* 2001, **7**(6):1321-1327.

18. Hoja U, Marthol S, Hofmann J, Stegner S, Schulz R, Meier S, Greiner E, Schweizer E: **HFA1 encoding an organelle-specific acetyl-CoA carboxylase controls mitochondrial fatty acid synthesis in Saccharomyces cerevisiae**. *J Biol Chem* 2004, **279**(21):21779-21786.

19. Zhang H, Yang Z, Shen Y, Tong L: **Crystal structure of the carboxyltransferase domain of acetyl-coenzyme A carboxylase**. *Science* 2003, **299**(5615):2064-2067.

20. Shen Y, Volrath SL, Weatherly SC, Elich TD, Tong L: **A mechanism for the potent inhibition of eukaryotic acetyl-coenzyme A carboxylase by soraphen A, a macrocyclic polyketide natural product**. *Mol Cell* 2004, **16**(6):881-891.

21. Jitrapakdee S, Wallace JC: **The biotin enzyme family: conserved structural motifs and domain rearrangements**. *Curr Protein Pept Sci* 2003, **4**(3):217-229.

22. Sommerhalter M, Voegtli WC, Perlstein DL, Ge J, Stubbe J, Rosenzweig AC: **Structures of the yeast ribonucleotide reductase Rnr2 and Rnr4 homodimers**. *Biochemistry* 2004, **43**(24):7736-7742.

23. Voegtli WC, Ge J, Perlstein DL, Stubbe J, Rosenzweig AC: **Structure of the yeast ribonucleotide reductase Y2Y4 heterodimer**. *Proc Natl Acad Sci U S A* 2001, **98**(18):10073-10078.

24. Yao R, Zhang Z, An X, Bucci B, Perlstein DL, Stubbe J, Huang M: **Subcellular localization of yeast ribonucleotide reductase regulated by the DNA replication and damage checkpoint pathways**. *Proc Natl Acad Sci U S A* 2003, **100**(11):6628-6633.

25. Lima CD, Wang LK, Shuman S: **Structure and mechanism of yeast RNA triphosphatase: an essential component of the mRNA capping apparatus**. *Cell* 1999, **99**(5):533-543.

26. Rodriguez CR, Takagi T, Cho EJ, Buratowski S: **A Saccharomyces cerevisiae RNA 5'-triphosphatase related to mRNA capping enzyme**. *Nucleic Acids Res* 1999, **27**(10):2181-2188.

27. Bisaillon M, Shuman S: **Structure-function analysis of the active site tunnel of yeast RNA triphosphatase**. *J Biol Chem* 2001, **276**(20):17261-17266.

28. Lehman K, Ho CK, Shuman S: **Importance of homodimerization for the in vivo function of yeast RNA triphosphatase**. *J Biol Chem* 2001, **276**(18):14996-15002.

29. Ho CK, Lehman K, Shuman S: **An essential surface motif (WAQKW) of yeast RNA triphosphatase mediates formation of the mRNA capping enzyme complex with RNA guanylyltransferase**. *Nucleic Acids Res* 1999, **27**(24):4671-4678.

30. Itoh N, Yamada H, Kaziro Y, Mizumoto K: **Messenger RNA guanylyltransferase from Saccharomyces cerevisiae. Large scale purification, subunit functions, and subcellular localization**. *J Biol Chem* 1987, **262**(5):1989-1995.

31. Horazdovsky BF, Busch GR, Emr SD: **VPS21 encodes a rab5-like GTP binding protein that is required for the sorting of yeast vacuolar proteins**. *Embo J* 1994, **13**(6):1297-1309.

32. Singer-Kruger B, Stenmark H, Dusterhoft A, Philippsen P, Yoo JS, Gallwitz D, Zerial M: **Role of three rab5-like GTPases, Ypt51p, Ypt52p, and Ypt53p, in the endocytic and vacuolar protein sorting pathways of yeast**. *J Cell Biol* 1994, **125**(2):283-298.

33. Esters H, Alexandrov K, Constantinescu AT, Goody RS, Scheidig AJ: **High-resolution crystal structure of S. cerevisiae Ypt51(DeltaC15)-GppNHp, a small GTP-binding protein involved in regulation of endocytosis**. *J Mol Biol* 2000, **298**(1):111-121.

34. Sprang SR: **G protein mechanisms: insights from structural analysis**. *Annu Rev Biochem* 1997, **66**:639-678.

35. Ostermeier C, Brunger AT: **Structural basis of Rab effector specificity: crystal structure of the small G protein Rab3A complexed with the effector domain of rabphilin-3A**. *Cell* 1999, **96**(3):363-374.

36. Schnabl M, Oskolkova OV, Holic R, Brezna B, Pichler H, Zagorsek M, Kohlwein SD, Paltauf F, Daum G, Griac P: **Subcellular localization of yeast Sec14 homologues and their involvement in regulation of phospholipid turnover**. *Eur J Biochem* 2003, **270**(15):3133-3145.

37. Mousley CJ, Tyeryar KR, Ryan MM, Bankaitis VA: **Sec14p-like proteins regulate phosphoinositide homoeostasis and intracellular protein and lipid trafficking in yeast**. *Biochem Soc Trans* 2006, **34**(Pt 3):346-350.

38. Li X, Routt SM, Xie Z, Cui X, Fang M, Kearns MA, Bard M, Kirsch DR, Bankaitis VA: **Identification of a novel family of nonclassic yeast phosphatidylinositol transfer proteins whose function modulates phospholipase D activity and Sec14p-independent cell growth**. *Mol Biol Cell* 2000, **11**(6):1989-2005.

39. Griac P, Holic R, Tahotna D: **Phosphatidylinositol-transfer protein and its homologues in yeast**. *Biochem Soc Trans* 2006, **34**(Pt 3):377-380.

40. Sha B, Phillips SE, Bankaitis VA, Luo M: **Crystal structure of the Saccharomyces cerevisiae phosphatidylinositol-transfer protein**. *Nature* 1998, **391**(6666):506-510.

41. Bankaitis VA, Phillips S, Yanagisawa L, Li X, Routt S, Xie Z: **Phosphatidylinositol transfer protein function in the yeast Saccharomyces cerevisiae**. *Adv Enzyme Regul* 2005, **45**:155-170.

42. Phillips SE, Sha B, Topalof L, Xie Z, Alb JG, Klenchin VA, Swigart P, Cockcroft S, Martin TF, Luo M *et al*: **Yeast Sec14p deficient in phosphatidylinositol transfer activity is functional in vivo**. *Mol Cell* 1999, **4**(2):187-197.

43. Martin-Yken H, Dagkessamanskaia A, Basmaji F, Lagorce A, Francois J: **The interaction of Slt2 MAP kinase with Knr4 is necessary for signalling through the cell wall integrity pathway in Saccharomyces cerevisiae**. *Mol Microbiol* 2003, **49**(1):23-35.

44. Schwartz MA, Madhani HD: **Principles of MAP kinase signaling specificity in Saccharomyces cerevisiae**. *Annu Rev Genet* 2004, **38**:725-748.

45. Wang Z, Harkins PC, Ulevitch RJ, Han J, Cobb MH, Goldsmith EJ: **The structure of mitogen-activated protein kinase p38 at 2.1-A resolution**. *Proc Natl Acad Sci U S A* 1997, **94**(6):2327-2332.

46. Wilson KP, Fitzgibbon MJ, Caron PR, Griffith JP, Chen W, McCaffrey PG, Chambers SP, Su MS: **Crystal structure of p38 mitogen-activated protein kinase**. *J Biol Chem* 1996, **271**(44):27696-27700.

47. Anderson NG, Maller JL, Tonks NK, Sturgill TW: **Requirement for integration of signals from two distinct phosphorylation pathways for activation of MAP kinase**. *Nature* 1990, **343**(6259):651-653.

48. Kumar S, McLaughlin MM, McDonnell PC, Lee JC, Livi GP, Young PR: **Human mitogen-activated protein kinase CSBP1, but not CSBP2, complements a hog1 deletion in yeast**. *J Biol Chem* 1995, **270**(49):29043-29046.

49. Gum RJ, McLaughlin MM, Kumar S, Wang Z, Bower MJ, Lee JC, Adams JL, Livi GP, Goldsmith EJ, Young PR: **Acquisition of sensitivity of stress-activated protein kinases to the p38 inhibitor, SB 203580, by alteration of one or more amino acids within the ATP binding pocket**. *J Biol Chem* 1998, **273**(25):15605-15610.

50. Tanoue T, Nishida E: **Docking interactions in the mitogen-activated protein kinase cascades**. *Pharmacol Ther* 2002, **93**(2-3):193-202.

51. Chang CI, Xu BE, Akella R, Cobb MH, Goldsmith EJ: **Crystal structures of MAP kinase p38 complexed to the docking sites on its nuclear substrate MEF2A and activator MKK3b**. *Mol Cell* 2002, **9**(6):1241-1249.

52. Hutter D, Chen P, Barnes J, Liu Y: **Catalytic activation of mitogen-activated protein (MAP) kinase phosphatase-1 by binding to p38 MAP kinase: critical role of the p38 C-terminal domain in its negative regulation**. *Biochem J* 2000, **352 Pt 1**:155-163.

53. Poon PP, Cassel D, Spang A, Rotman M, Pick E, Singer RA, Johnston GC: **Retrograde transport from the yeast Golgi is mediated by two ARF GAP proteins with overlapping function**. *Embo J* 1999, **18**(3):555-564.

54. Poon PP, Wang X, Rotman M, Huber I, Cukierman E, Cassel D, Singer RA, Johnston GC: **Saccharomyces cerevisiae Gcs1 is an ADP-ribosylation factor GTPase-activating protein**. *Proc Natl Acad Sci U S A* 1996, **93**(19):10074-10077.

55. Wang X, Hoekstra MF, DeMaggio AJ, Dhillon N, Vancura A, Kuret J, Johnston GC, Singer RA: **Prenylated isoforms of yeast casein kinase I, including the novel Yck3p, suppress the gcs1 blockage of cell proliferation from stationary phase**. *Mol Cell Biol* 1996, **16**(10):5375-5385.

56. Coe JG, Murray LE, Dawes IW: **Identification of a sporulation-specific promoter regulating divergent transcription of two novel sporulation genes in Saccharomyces cerevisiae**. *Mol Gen Genet* 1994, **244**(6):661-672.

57. Mandiyan V, Andreev J, Schlessinger J, Hubbard SR: **Crystal structure of the ARF-GAP domain and ankyrin repeats of PYK2-associated protein beta**. *Embo J* 1999, **18**(24):6890-6898.

58. Goldberg J: **Structural and functional analysis of the ARF1-ARFGAP complex reveals a role for coatomer in GTP hydrolysis**. *Cell* 1999, **96**(6):893-902.

59. Pearce AK, Crimmins K, Groussac E, Hewlins MJ, Dickinson JR, Francois J, Booth IR, Brown AJ: **Pyruvate kinase (Pyk1) levels influence both the rate and direction of carbon flux in yeast under fermentative conditions**. *Microbiology* 2001, **147**(Pt 2):391-401.

60. Portela P, Howell S, Moreno S, Rossi S: **In vivo and in vitro phosphorylation of two isoforms of yeast pyruvate kinase by protein kinase A**. *J Biol Chem* 2002, **277**(34):30477-30487.

61. Boles E, Schulte F, Miosga T, Freidel K, Schluter E, Zimmermann FK, Hollenberg CP, Heinisch JJ: **Characterization of a glucose-repressed pyruvate kinase (Pyk2p) in Saccharomyces cerevisiae that is catalytically insensitive to fructose-1,6-bisphosphate**. *J Bacteriol* 1997, **179**(9):2987-2993.

62. Sonderegger M, Jeppsson M, Hahn-Hagerdal B, Sauer U: **Molecular basis for anaerobic growth of Saccharomyces cerevisiae on xylose, investigated by global gene expression and metabolic flux analysis**. *Appl Environ Microbiol* 2004, **70**(4):2307-2317.

63. Jurica MS, Mesecar A, Heath PJ, Shi W, Nowak T, Stoddard BL: **The allosteric regulation of pyruvate kinase by fructose-1,6-bisphosphate**. *Structure* 1998, **6**(2):195-210.

64. Collins RA, McNally T, Fothergill-Gilmore LA, Muirhead H: **A subunit interface mutant of yeast pyruvate kinase requires the allosteric activator fructose 1,6-bisphosphate for activity**. *Biochem J* 1995, **310 ( Pt 1)**:117-123.

65. Feldmann H, Aigle M, Aljinovic G, Andre B, Baclet MC, Barthe C, Baur A, Becam AM, Biteau N, Boles E *et al*: **Complete DNA sequence of yeast chromosome II**. *Embo J* 1994, **13**(24):5795-5809.

66. Leskovac V, Trivic S, Pericin D: **The three zinc-containing alcohol dehydrogenases from baker's yeast, Saccharomyces cerevisiae**. *FEMS Yeast Res* 2002, **2**(4):481-494.

67. Dickinson JR, Salgado LE, Hewlins MJ: **The catabolism of amino acids to long chain and complex alcohols in Saccharomyces cerevisiae**. *J Biol Chem* 2003, **278**(10):8028-8034.

68. Smith MG, Des Etages SG, Snyder M: **Microbial synergy via an ethanol-triggered pathway**. *Mol Cell Biol* 2004, **24**(9):3874-3884.

69. Turner RJ, Lovato M, Schimmel P: **One of two genes encoding glycyl-tRNA synthetase in Saccharomyces cerevisiae provides mitochondrial and cytoplasmic functions**. *J Biol Chem* 2000, **275**(36):27681-27688.

70. Magrath C, Hyman LE: **A mutation in GRS1, a glycyl-tRNA synthetase, affects 3'-end formation in Saccharomyces cerevisiae**. *Genetics* 1999, **152**(1):129-141.

71. Otte S, Belden WJ, Heidtman M, Liu J, Jensen ON, Barlowe C: **Erv41p and Erv46p: new components of COPII vesicles involved in transport between the ER and Golgi complex**. *J Cell Biol* 2001, **152**(3):503-518.

72. Powers J, Barlowe C: **Transport of axl2p depends on erv14p, an ER-vesicle protein related to the Drosophila cornichon gene product**. *J Cell Biol* 1998, **142**(5):1209-1222.

73. Gillingham AK, Tong AH, Boone C, Munro S: **The GTPase Arf1p and the ER to Golgi cargo receptor Erv14p cooperate to recruit the golgin Rud3p to the cis-Golgi**. *J Cell Biol* 2004, **167**(2):281-292.

74. Nakanishi H, Suda Y, Neiman AM: **Erv14 family cargo receptors are necessary for ER exit during sporulation in Saccharomyces cerevisiae**. *J Cell Sci* 2007, **120**(Pt 5):908-916.

75. Rossler H, Rieck C, Delong T, Hoja U, Schweizer E: **Functional differentiation and selective inactivation of multiple Saccharomyces cerevisiae genes involved in very-long-chain fatty acid synthesis**. *Mol Genet Genomics* 2003, **269**(2):290-298.

76. Tehlivets O, Scheuringer K, Kohlwein SD: **Fatty acid synthesis and elongation in yeast**. *Biochim Biophys Acta* 2007, **1771**(3):255-270.
